# Molecular insight into 5′ RNA capping with Np_*n*_Ns by bacterial RNA polymerase

**DOI:** 10.1038/s41589-025-02134-5

**Published:** 2026-01-09

**Authors:** Valentina M. Serianni, Jana Škerlová, Anna Knopp Dubánková, Anton Škríba, Hana Šváchová, Tereza Vučková, Anatolij Filimoněnko, Milan Fábry, Pavlína Řezáčová, Tomáš Kouba, Hana Cahova

**Affiliations:** 1 Institute of Organic Chemistry and Biochemistry of the CAS, Flemingovo náměstí 2, Prague, Czechia; 2 Charles University, Faculty of Science, Department of Cell Biology, Viničná 7, Prague, Czechia

**Keywords:** RNA, Transcription, Structural biology

## Abstract

RNA capped with dinucleoside polyphosphates has been discovered in bacteria and eukaryotes only recently. The likely mechanism of this specific capping involves direct incorporation of dinucleoside polyphosphates by RNA polymerase as noncanonical initiating nucleotides. However, how these compounds bind into the active site of RNA polymerase during transcription initiation is unknown. Here, we explored transcription initiation in vitro, using a series of DNA templates in combination with dinucleoside polyphosphates and model RNA polymerase from *Thermus* *thermophilus*. We observed that the transcription start site can vary on the basis of the compatibility of the specific template and dinucleoside polyphosphate. Cryo-electron microscopy structures of transcription initiation complexes with dinucleoside polyphosphates revealed that both nucleobase moieties can pair with the DNA template. The first encoded nucleotide pairs in a canonical Watson–Crick manner, whereas the second nucleobase pairs noncanonically in a reverse Watson–Crick manner. Our work provides a structural explanation of how dinucleoside polyphosphates initiate RNA transcription.

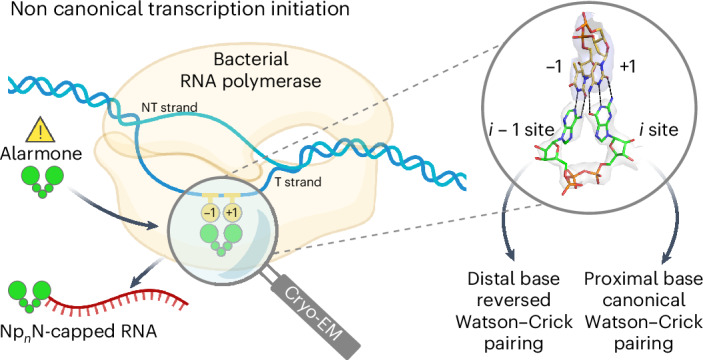

## Abstract (web summary)

Using cryo-electron microscopy technologies, Serianni and Škerlová et al. reveal how Np_*n*_Ns initiate bacterial transcription as noncanonical RNA caps by showing one nucleobase pairing with the template in canonical mode while the other pairs in reverse Watson–Crick mode.

## Main

RNA modifications have important roles in the life of RNA, including its stabilization, localization and specific recognition of its interacting partners. More than 170 RNA modifications are known^1^ in all types of organisms. Their roles are understood and well described in the most abundant types of RNA such as transfer RNA and ribosomal RNA. However, their function in other types of RNA, like mRNA or long noncoding RNA, is still under investigation. Some of the best-described RNA modifications are canonical 5′ m^7^G caps, which are known to have an important role in eukaryotic mRNAs. The discovery of 5′ noncanonical capping of RNA, namely the capping of RNA with nicotinamide adenine dinucleotide (NAD)^2–5^ and coenzyme A (CoA)^6^, triggered a search for other alternative RNA caps. Lately, flavin adenine dinucleotide (FAD)^7,8^, sugar conjugate^9^ and dinucleoside polyphosphate (Np_*n*_Ns)^10–12^ RNA caps have been discovered in a variety of organisms^13^.

The most widely accepted hypothesis concerning the biosynthesis of noncanonically capped RNA is that RNA polymerases (RNAPs) directly use these molecules as noncanonical initiating nucleotides (NCINs). This is analogous to noncanonical transcription initiation by 5′-end hydroxyl dinucleotide primers^14,15^, which are used instead of regular nucleoside triphosphates (NTPs). The ability of bacterial or bacteriophage RNAP to initiate transcription with NAD, CoA or FAD cofactor molecules was first described in vitro^16,17^. Later on, cofactor incorporation by bacterial and eukaryotic RNAP II was confirmed in vivo, as well^18^. X-ray structures of *Thermus* *thermophilus* (*Tt*) RNAP visualized these cofactors as NCINs^18^.

Various types of Np_*n*_Ns serving as RNA caps have been detected in *Escherichia* *coli* by using liquid chromatography–mass spectrometry (LC–MS) analysis^11^. We and others have demonstrated that Np_*n*_Ns can be incorporated into RNA directly by bacteriophage and bacterial RNAPs as NCINs^11,19^ in a similar manner as cofactors.

**Fig. 1:**
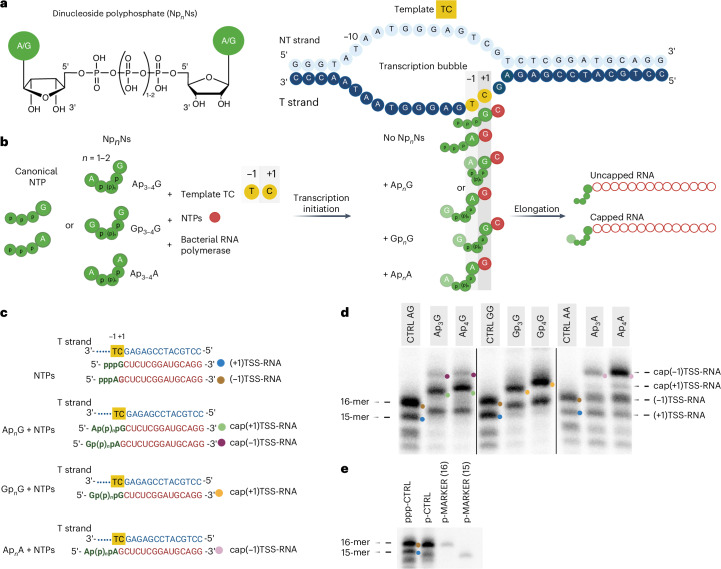
IVT reactions with Np_*n*_Ns and template TC. **a**, Chemical structure of Np_*n*_Ns. **b**, Schemes of IVT experiments where NTPs or Np_*n*_Ns bind to the template TC (with a premelted bubble from position −11 to +2) during transcription initiation with different base-pairing combinations between Np_*n*_Ns and the −1 and +1 template strand positions. T, template; NT, nontemplate. **c**, Sequences of color-coded RNA products with and without the addition of Np_*n*_Ns. Uncapped 15-mer referred to as (+1)TSS-RNA is indicated by a blue dot, uncapped 16-mer referred to as (−1)TSS-RNA is indicated by a brown dot, capped 15-mers referred to as cap(+1)TSS-RNA are indicated by green and yellow dots and capped 16-mers referred to as cap(−1)TSS-RNA are indicated by purple and pink dots. **d**, PAGE analysis with acryloylaminophenyl boronic acid (APB) showing RNA products obtained from IVT reactions with the template TC and NTPs or their combination with various Np_*n*_Ns radiolabeled with α-[^32^P]CTP. Each control (CTRL AG, CTRL GG, CTRL AA) was designed according to Supplementary Table 2; it shows the migration of the uncapped RNA 15-mer (blue dots) and 16-mer (brown dots) and serves as a molecular weight marker (**e**). The capped products are marked with colored dots according to **c**. The IVT was performed in ten independent experiments; one representative gel is shown. Nonadjacent lanes from the same experiment were rearranged and are separated by delineating black lines. Each lane originates from the same gel. **e**, PAGE (without APB) analysis showing the migration patterns of a 16-mer and 15-mer monophosphate marker along with the triphosphate RNA (ppp-CTRL) and the monophosphate RNA (p-CTRL) generated using template TC and regular NTPs. The markers have the same sequence as the RNA transcript expected from the template TC (Extended Data Table 1), starting at position −1 (p-MARKER(16)) and +1 (p-MARKER(15)). This experiment was performed in triplicate; one representative gel is shown. Created in BioRender. Serianni, V. (2025) https://BioRender.com/p388ku2. Source Data

**Extended Data Fig. 1:**
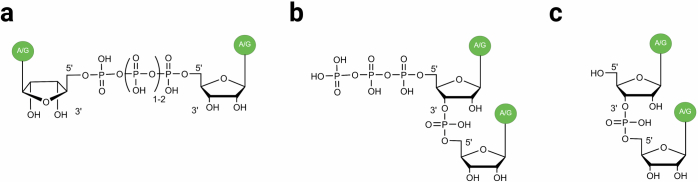
Distinct phosphodiester linkages in Np_*n*_Ns and canonical RNA. Chemical structures of Np_*n*_Ns (**a**), pppNpN nascent RNA dinucleotide (**b**) and NpN 5′-end hydroxyl dinucleotide primer (**c**). Created in BioRender. Serianni, V. (2025) https://BioRender.com/qasouxb.

In our previous study, we focused on the direct incorporation of Np_*n*_Ns by RNAP during the initiation phase of transcription^20^. We observed that the addition of Np_*n*_Ns, such as Ap_3_G, into an in vitro transcription (IVT) reaction with T7 RNAP led to a several times greater production of 2-mer or 3-mer RNAs. It is important to note that because Np_*n*_Ns are linked 5′ to 5′ (Fig. 1a and Extended Data Fig. 1), they have two free 3′ hydroxyl groups and can be incorporated into a nascent RNA chain in two alternative orientations. Molecular dynamics simulations predicted that both nucleosides from the Ap_3_G NCIN pair with the template DNA strand. Whereas G paired in the RNAP *i* site with the template transcription start site (TSS) +1C in a Watson–Crick (WC) manner, A paired in the *i* − 1 site with the template −1T in a noncanonical manner. In comparison, the 5′-end hydroxyl dinucleotide primers, where the direction of incorporation of the dinucleotides is governed by the canonical 5′-to-3′ linkage, pair in the regular WC manner in both *i* and *i* − 1 sites^15^.

To further investigate the details of the initiation phase of transcription with NCINs in bacteria, we focused on *Tt* RNAP as a robust structural model of bacterial transcription and on purine-containing Np_*n*_Ns, which are the most studied examples of Np_*n*_Ns in bacteria^21–23^. Moreover, *Tt* σ^A^ belongs to the same family of sigma factors^24^ as housekeeping *E*. *coli* σ^70^, which has been reported to initiate transcription primarily with ATP or GTP^25^. We designed experiments where various Np_*n*_Ns (Ap_3-4_A, Ap_3-4_G and Gp_3-4_G) were probed as NCINs by IVT assays with templates having combinations of T and/or C at positions −1 and +1 of the template strand with respect to the TSS. Following on these experiments, we visualized, using cryogenic electron microscopy (cryo-EM), a set of *Tt* RNAP transcription initiation complexes with various Ap_*n*_Ns bound as NCINs to templates in the RNAP active site (AS). The structural analysis confirmed that both nucleosides of Ap_*n*_Ns can base pair with the template DNA strand and provided a structural basis for understanding their role as NCINs.

## Results

### Two possible ways of transcription initiation with Np_*n*_Ns

To explore the role of Np_*n*_Ns in the transcription initiation process, we designed a series of templates with artificial transcription bubbles for use in an IVT assay using the *Tt* RNAP-σ^A^ holoenzyme^26^. The templates were designed on the basis of previous work^27,28^ (Fig. 1, Extended Data Table 1, Supplementary Table 1 and Supplementary Fig. 1), having combinations of C and/or T at positions −1 and +1 of the template strand with respect to the TSS. We then performed radioactively labeled IVT reactions using these templates with four regular NTPs in combination with Np_*n*_Ns (Ap_3-4_A, Ap_3-4_G and Gp_3-4_G; Fig. 1b,c). We prepared control experiments where the concentration of Np_*n*_Ns was replaced by the two respective NTPs (ATP or GTP). Each control contained the same total final concentration of initiating nucleotides (Supplementary Table 2).

The control reactions using the template TC and regular NTPs resulted in the production of the expected 15-mer product ((+1)TSS-RNA), initiating at the putative +1C TSS (Fig. 1c,d). We also observed formation of a 1-nt-longer product potentially starting at the −1T TSS ((−1)TSS-RNA) and other shorter aberrant products (Fig. 1c,d), which might be abortive products, as previously reported for IVTs using *E*. *coli* RNAP^29,30^. When we used the template TC and added Np_*n*_Ns into the IVT mixture, we observed the formation of capped RNA of two different lengths, depending on the type of Np_*n*_N (Fig. 1c,d). The addition of Ap_3-4_G and Gp_3-4_G in the reaction mixture led to the formation of a dominant RNA product migrating slower in PAGE than the control RNA without Np_*n*_Ns (15-mer). The size of this RNA corresponds to a capped 15-mer transcription product starting with the G nucleoside of Np_*n*_G at position +1C of the template (cap(+1)TSS-RNA). This potentially allows the A nucleoside of Ap_3-4_G to interact with the −1T base of the template (Fig. 1b). In experiments with added Ap_3-4_G, we also observed traces of a capped 16-mer RNA product (cap(−1)TSS-RNA). This suggests that Ap_3-4_G can initiate transcription with the A moiety at the template −1T position. However, this would require a shift of the template strand by one nucleotide with respect to the RNAP AS for the −1 template position to serve as the TSS. Variability in TSS selection has been already described and involves DNA scrunching and antiscrunching^31–33^. Lastly, when we added Ap_3-4_A to the IVT reactions, we observed the formation of the capped 16-mer transcription product (cap(−1)TSS-RNA; Fig. 1d). This suggests that initiation by Ap_3-4_A leads to the production of capped RNA encoded from the −1T position rather than from the putative +1C position of the DNA template.

**Extended Data Fig. 2:**
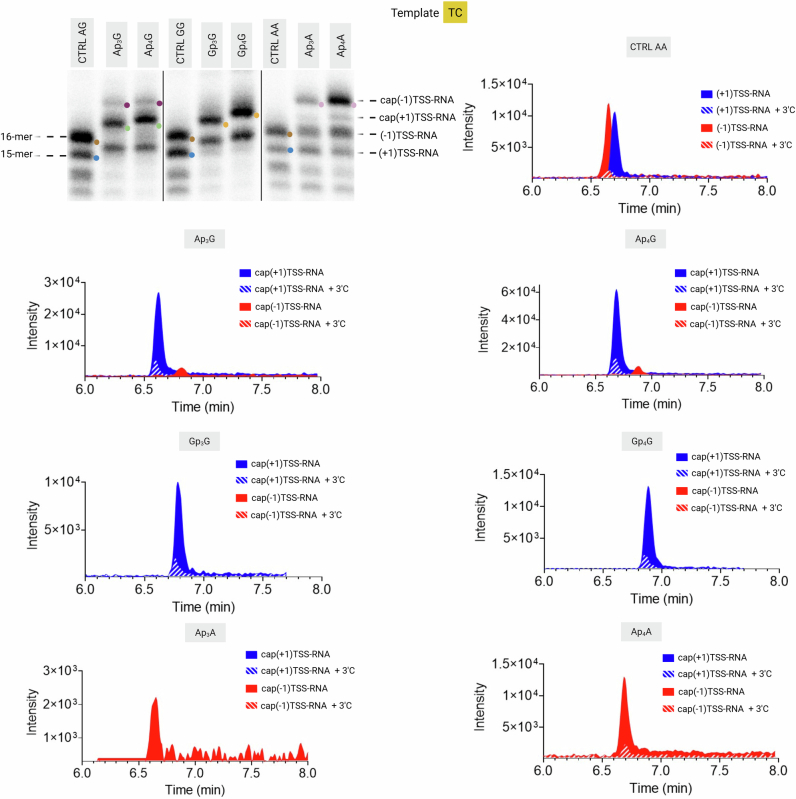
LC-MS analysis of IVT products with template TC. Extracted ion chromatograms of IVT products with and without addition of Np_*n*_Ns. IVT products encoded from the +1 TSS are marked blue, IVT products encoded from the +1 TSS with an extra C added at the 3′ end are marked dashed blue. IVT products encoded from the −1 TSS are marked red, IVT products encoded from the −1 TSS with an extra C added at the 3′ end are marked dashed red. The sequences of the formed products with exact masses are shown in Supplementary Table 3. The PAGE gel from Fig. 1d is reproduced here for clarity (see Fig. 1c-e legend). Created in BioRender. Serianni, V. (2025) https://BioRender.com/55wowug.

To confirm the results of the IVT PAGE experiments and, thus, verify that we observed Np_*n*_N-capped RNA products initiating at the +1 and −1 template positions as the TSSs, we established a very sensitive LC–MS method capable of detecting the full-length products of IVT with *Tt* RNAP. We were able to identify all expected IVT products, encoded from both the +1 TSS and the −1 TSS (Fig. 1c), in the reaction mixtures containing Np_*n*_Ns or in the control mixtures (Supplementary Table 3 and Extended Data Fig. 2). Surprisingly, we also detected unexpected products with an extra C added at the 3′ end (Supplementary Table 3, Extended Data Fig. 2 and Supplementary Fig. 2), indicating that, for example, the IVT PAGE band corresponding to a 16-mer (Fig. 1d) contains, in addition to the (−1)TSS-RNA, a (+1)TSS-RNA with an extra 3′ C. Similar nontemplated addition of C at the 3′ end of RNA has been already reported for mitochondrial RNAP of the protist *Physarum* *polycephalum*^34,35^.

**Extended Data Fig. 3:**
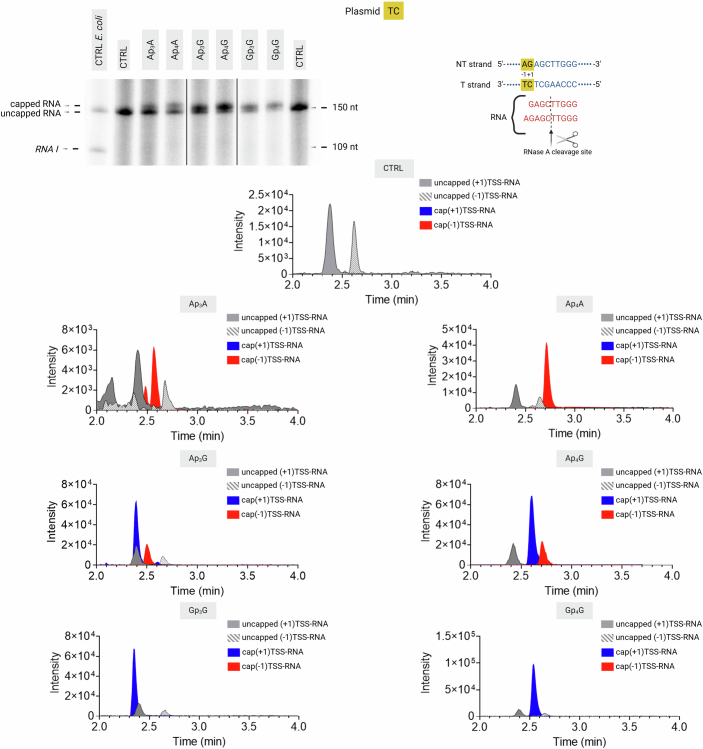
PAGE analysis and LC-MS analysis of IVT products generated from plasmid TC. PAGE analysis (with APB) shows RNA products obtained from IVT reactions using the TC plasmid and NTPs or their combination with Np_*n*_Ns radiolabeled with α-[^32^P]CTP. The IVT reaction was performed in five independent experiments. The controls (CTRL *E. coli* and CTRL) show the migration of the uncapped RNA (150 nt) produced by *E. coli* RNAP and *Tt* RNAP, respectively, and serve as molecular weight markers. Moreover, the formation of the known RNAI encoded in the plasmid (109 nt) and produced by *E. coli* RNAP was observed. Non-radiolabeled RNA was used for digestion with RNase A (cleaving after pyrimidines) and further analyzed by LC-MS. Extracted ion chromatograms show RNA fragments generated from IVT with or without Np_*n*_Ns. Uncapped RNA (CTRL) is indicated in gray: solid gray lines represent transcripts initiating at the +1 TSS (defined as uncapped(+1)TSS-RNA), while dashed gray lines represent transcripts initiating at the −1 TSS (defined as uncapped(−1)TSS-RNA). Capped IVT products encoded from the +1 TSS (cap(+1)TSS-RNA) are shown in blue and products encoded from the −1 TSS (cap(−1)TSS-RNA) are shown in red. The sequences and exact masses of the corresponding products are listed in Supplementary Table 4. Created in BioRender. Serianni, V. (2025) https://BioRender.com/pbscak2. Source Data

To understand whether capped RNA is also formed by *Tt* RNAP in the context of native-like DNA promoter, we developed another transcription assay using a circular supercoiled plasmid (plasmid TC)^36^ Supplementary Table 1) containing a fully complementary promoter with the −35 and −10 elements used in this study and with a T at the −1 position and a C at the +1 position, resembling the TC template. Using this assay, we observed the formation of all Np_*n*_N-capped RNAs through PAGE and LC–MS analysis combined with RNase A treatment (Extended Data Fig. 3 and Supplementary Table 4).

**Extended Data Fig. 4:**
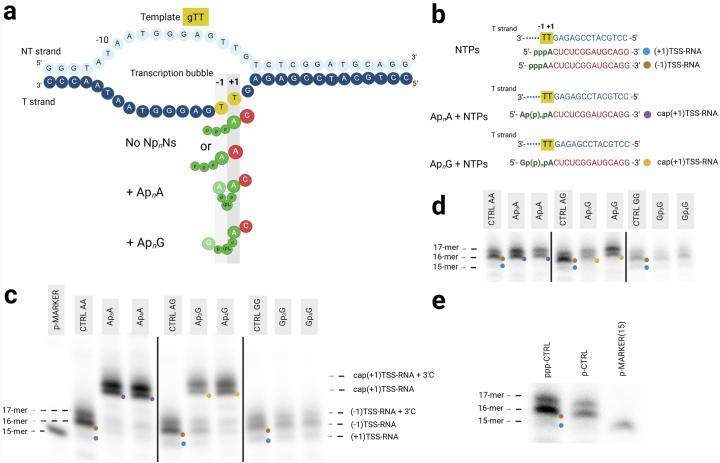
IVT reactions with Np_*n*_Ns and the template gTT. **a**, Schemes of IVT experiments showing NTPs or Np_*n*_Ns binding to the template gTT (with a pre-melted bubble from position −11 to +2) during transcription initiation with different base pairing at the −1 and +1 positions. **b**, Sequences of color-coded RNA products with and without the addition of Np_*n*_Ns. Uncapped 15-mer referred to as (+1)TSS-RNA is indicated by a blue dot, uncapped 16-mer referred to as (−1)TSS-RNA is indicated by a brown dot, capped 15-mers referred to as cap(+1)TSS-RNA are indicated by violet and yellow dots. **c**, PAGE (with APB) showing RNA products obtained from IVT reactions with the template gTT and NTPs or their combination with Np_*n*_Ns radiolabeled with α-[^32^P]CTP. Each control (CTRL AG, CTRL GG, CTRL AA), designed according to Supplementary Table 2, shows the migration of the uncapped RNA 15-mer and 16-mer and serves as a molecular weight marker (panel **e**). The capped products are marked with colored dots according to panel **b**. We observed also the formation of uncapped 17-mer with an extra C added at the 3′ end. The label ‘+ 3′C’ indicates RNA transcripts with an extra 3′ C, as determined by LC-MS (see Extended Data Figure 5). The IVT was performed in ten independent experiments; one representative gel is shown. **d**, PAGE (without APB) was used to analyze RNA products obtained from IVT reactions. The reactions were performed as in panel **c** and it shows that, without APB, there is no difference in migration between capped and uncapped RNA. The controls/markers are analogous to panel **c**. **e**, PAGE (without APB) shows the migration of 15-mer monophosphate marker (p-MARKER(15)) along with the monophosphate RNA (p-CTRL) and triphosphate RNA (ppp-CTRL) generated using template gTT and regular NTPs. The experiment was performed in triplicate. Created in BioRender. Serianni, V. (2025) https://BioRender.com/fggd152. Source Data

**Extended Data Fig. 5:**
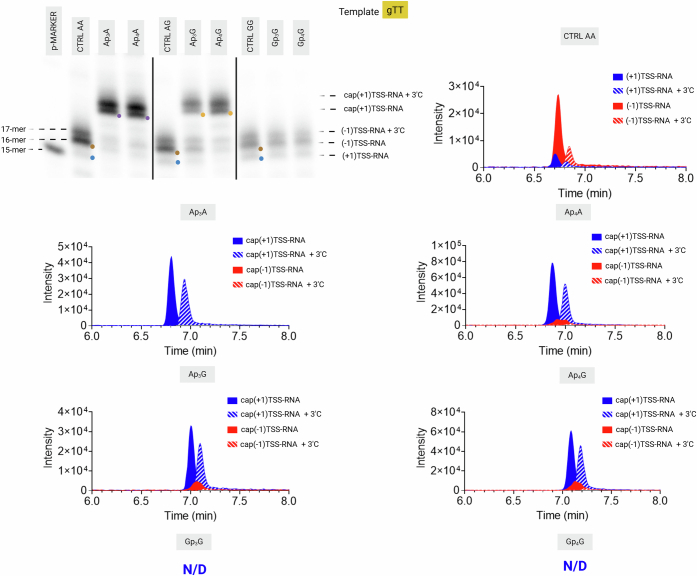
LC-MS analysis of IVT products with template gTT. The PAGE gel from Extended Data Fig. 4c is reproduced here for clarity. Extracted ion chromatograms of IVT products with and without addition of Np_*n*_Ns. IVT products encoded from the +1 TSS are marked blue, IVT products encoded from the +1 TSS with an extra C added at the 3′ end are marked dashed blue. IVT products encoded from the −1 TSS are marked red, IVT products encoded from the −1 TSS with an extra C added at the 3′ end are marked dashed red. The sequences of the formed products with exact masses are shown in Supplementary Table 5. We confirmed the 16-mer product encoded from the −1 TSS and we detected a 17-mer RNA product corresponding with its mass also to the (−1)TSS-RNA but with an extra C added at the 3′ end. Only trace amounts of the 15-mer RNA encoded from the +1 TSS were detected by PAGE and LC-MS. The PAGE analysis of IVT reactions with Ap_3-4_A and the template gTT showed the formation of two capped products. LC-MS analysis confirmed the production of capped RNA encoded from the +1 TSS, but contrary to the control IVT, we did not detect capped RNA encoded from the −1 TSS. The second observed product has the mass of cap(+1)TSS-RNA with the 3′ extra C. These observations indicate that Ap_3-4_A initiate transcription only from the +1 TSS of the gTT template. The experiment with Ap_3-4_G led to the formation of similar products as in the case of Ap_3-4_A. As expected, Gp_3-4_G cannot base pair with the gTT template and did not produce capped RNA. N/D = not detected. Created in BioRender. Serianni, V. (2025) https://BioRender.com/jj7ixi3. Source Data

**Extended Data Fig. 6:**
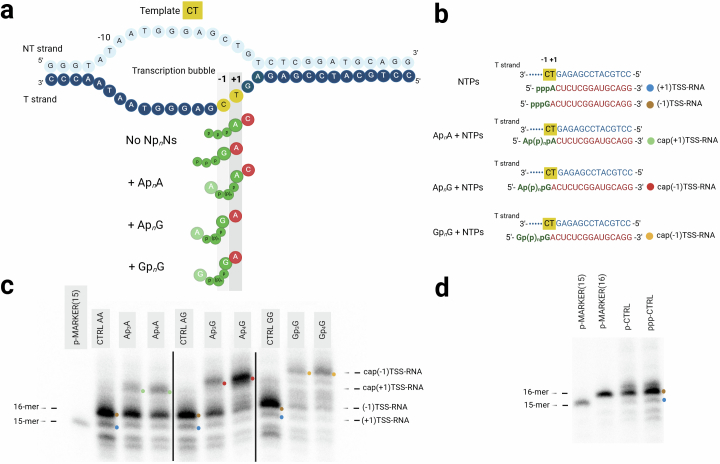
IVT with Np_*n*_Ns and template CT. **a**, Schemes of IVT experiments showing NTPs or Np_*n*_Ns binding to the template CT (with a pre-melted bubble from position −11 to +2) during transcription initiation with different base pairing at the −1 and +1 positions. **b**, Sequences of color-coded RNA products with and without the addition of Np_*n*_Ns. Uncapped 15-mer referred to as (+1)TSS-RNA is indicated by a blue dot, uncapped 16-mer referred to as (−1)TSS-RNA is indicated by a brown dot, capped 15-mer referred to as cap(+1)TSS-RNA is indicated by a green dot, and capped 16-mers referred to as cap(−1)TSS-RNA are indicated by red and yellow dots. **c**, PAGE (with APB) showing RNA products from IVT using the template CT with NTPs or various Np_*n*_Ns, radiolabeled with α-[^32^P]CTP. Each control (CTRL AG, CTRL GG, CTRL AA, Supplementary Table 2) shows the migration of the uncapped RNA 15-mer and 16-mer and serves as size marker (panel **d**). IVT reaction (Extended Data Table 1) without Np_*n*_Ns led to the production of mainly 16-mer and also 15-mer uncapped products. The RNA products are marked according to panel **b**. We observed formation of capped 15-mer RNA encoded from the +1 T position when Ap_3-4_A were added to IVT. When Ap_3-4_G and Gp_3-4_G were added, we observed mainly capped 16-mer RNA encoded from the −1 C position of the template. This indicates that the transcription was initiated by the G nucleoside of Gp_*n*_Ns bound at the −1 C position of the template. The IVT was performed in ten independent experiments; one representative gel is shown. **d**, PAGE (without APB) shows migration of 15-mer (p-MARKER(15)) and 16-mer (p-MARKER(16)) monophosphate marker along with the monophosphate RNA (p-CTRL) and triphosphate RNA (ppp-CTRL) generated using template CT and regular NTPs. The experiment was performed in triplicate. Created in BioRender. Serianni, V. (2025) https://BioRender.com/opxr6fh. Source Data

**Extended Data Fig. 7:**
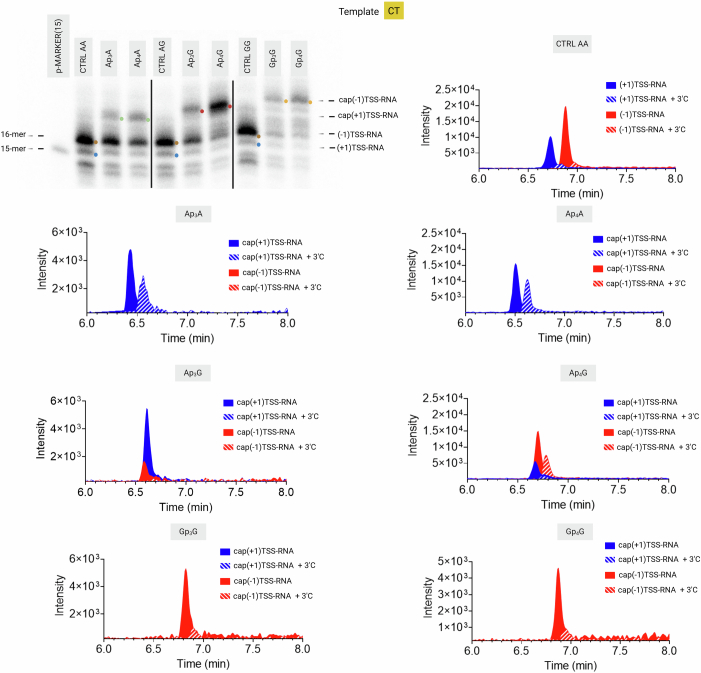
LC-MS analysis of IVT products with template CT. The PAGE gel from Extended Data Fig. 6c is reproduced here for clarity. Extracted ion chromatograms of IVT products with and without addition of Np_*n*_Ns. IVT products encoded from the +1 TSS are marked blue, IVT products encoded from the +1 TSS with an extra C added at the 3′ end are marked dashed blue. IVT products encoded from the −1 TSS are marked red, IVT products encoded from the −1 TSS with an extra C added at the 3′ end are marked dashed red. The sequences of the formed products with exact masses are shown in Supplementary Table 6. Majority of our observations from the PAGE analysis were also confirmed by the LC-MS analysis. The only exception was the reaction with Ap_3_G, which led to the formation of capped RNA encoded from the +1 TSS as the main product. We also observed the formation of products with an extra C added at the 3′ end. Created in BioRender. Serianni, V. (2025) https://BioRender.com/xnjca7i. Source Data

Furthermore, we performed the same IVT and LC–MS experiments as for template TC also on additional templates containing combinations of C and/or T at positions −1 and +1 of the template strand (templates gTT and CT) (Extended Data Table 1, Extended Data Figs. 4–7 and Supplementary Tables 5 and 6). Indeed, we confirmed that the *Tt* RNAP holoenzyme in combination with the DNA templates used in this study allow the Np_*n*_Ns to initiate transcription from both the +1 and −1 template positions as TSSs. Additionally, when using NAD as NCIN in the IVT, we confirmed that it has a much lower incorporation efficiency^19^ than NTPs and Np_*n*_Ns (Supplementary Fig. 3).

### Structural insights into transcription initiation by Np_*n*_Ns

During transcription initiation, RNAP catalyzes the synthesis of the first phosphodiester bond between the initiating nucleotide bound in the *i* site (+1 with respect to the canonical TSS of the template) and the extending nucleotide bound in the *i* + 1 site (+2 with respect to the canonical TSS of the template) of the catalytic site. Previous structural studies defined NCINs to be able to react de novo in the catalytic site of bacterial RNAP^18^. However, the binding of NCINs into the *i* site and the *i* − 1 site (−1 with respect to the canonical TSS of the template) to react with an extending nucleotide was never captured. This is because the NCINs were allowed to react with the extending nucleotide and the AS was visualized in a posttranslocated state. Here, we aimed to visualize the state just before the very first step of the initial nucleotidyl transfer, where NCINs align to react with the first extending nucleotide (Supplementary Fig. 4), but before the reaction occurs—in the precatalytic state. For this purpose, we reconstituted an artificial *Tt* holoenzyme complex with an opened transcription bubble with defined mixtures of substrates and nonhydrolyzable analogs.

**Extended Data Fig. 8:**
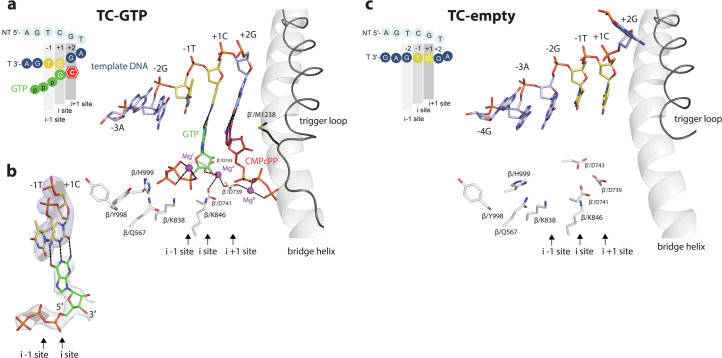
GTP bound in de novo transcription initiation complex; anti-scrunched template strand in the TC-empty complex. **a**, AS structure of de novo transcription initiation, where GTP binds canonically in the *i* site and CMPcPP is bound in a pre-insertion position in the *i* + 1 site, consistent with the trigger loop being only partially closed towards the AS. The DNA template strand is marked with respect to the +1 TSS site. The aspartate triad of the AS coordinates Mg^A^ and partially also Mg^B^. Conserved residues are positioning the GTP phosphates, which themselves coordinate an additional Mg^C^ ion. **b**, Cryo-EM density for the GTP in the *i* site (transparent gray), base pairing with the template +1 C (transparent blue). **c**, When the AS is unoccupied, the template strand is antiscrunched by one nucleotide in respect to when the AS is occupied by GTP and CMPcPP (panel **a**). The template −2 G base is in line with the *i* − 1 site, the template −1 T base is in line with the *i* site and the template +1 C base is in line with the *i* + 1 site. The +2 G base is shifted over the bridge helix towards the downstream DNA duplex.

We first visualized, using cryo-EM, the canonical de novo transcription initiation complex with template TC together with GTP and the nonhydrolyzable CTP analog cytidine-5′-[(α,β)-methyleno]triphosphate (CMPcPP). The cryo-EM analysis (Supplementary Figs. 5 and 6 and Supplementary Tables 7 and 8) revealed two relevant structures, one with an unoccupied AS, hereafter referred to as TC-empty, and one with the AS occupied by GTP and CMPcPP, hereafter referred to as TC-GTP. The TC-GTP structure (Extended Data Fig. 8a,b, Supplementary Figs. 4a and 7a and Supplementary Table 7) is similar to crystallographic structures of analogous complexes (PDB 4Q4Z (ref. ^37^) and PDB 4OIO (ref. ^38^)). The initiating GTP is bound in the *i* site and the extending CMPcPP in the *i* + 1 site, both canonically base pairing with the TSS +1C and +2G base, respectively. Mg^A^ is coordinated by the catalytic aspartate triad, whereas Mg^B^ is only partially coordinated by β′/D739. Notably, there is interpretable density for Mg^2+^ (hereafter Mg^C^), which is coordinated by phosphates of the GTP in a tridentate manner. The GTP α and γ phosphates canonically interact with conserved residues^37^ β/K838 and K846, and β/Q567 and H999, respectively. Additionally, residue β/Y998 adopts two alternative conformations, one pointing away and one pointing toward the γ phosphate.

In the TC-empty structure (Supplementary Fig. 4a and Extended Data Fig. 8c), we observed that the template strand is antiscrunched^31,33,39^ by one nucleotide, placing the template −2G base in line with the *i* − 1 site, the template −1T base in line with the *i* site and the template +1C base in line with the *i* + 1 site, while the +2G base is shifted over the bridge helix toward the downstream DNA duplex (Extended Data Fig. 8c and Supplementary Fig. 8a–c). The nontemplate strand is not shifted in register, which is highlighted by the +2G base of the nontemplate strand bound in the core recognition element (CRE)-specific binding pocket of RNAP^27^ (Supplementary Fig. 8a–c). As the AS is unoccupied, the antiscrunching of the template by one nucleotide and the concomitant TSS shift happens without any stabilization by nucleotides pairing with the antiscrunched template. This template oscillation^32^ then allows the use of both +1 and −1 TSS in the IVT reactions in our in vitro system. Altogether, the TC-GTP and TC-empty structures set a basis for structural comparison of complexes with various bound Np_*n*_Ns visualized in this study.

### Distal base binds template in a reverse WC (rWC) manner

**Fig. 2:**
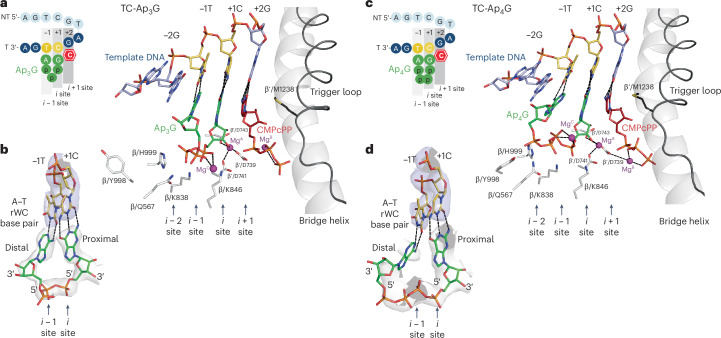
Ap_3_G and Ap_4_G bound in de novo transcription initiation complexes. **a**, AS structure of de novo transcription initiation, where Ap_3_G guanine binds canonically in the *i* site and adenine binds noncanonically in the *i* *−* 1 site. CMPcPP is bound in a preinsertion position in the *i* + 1 site, stabilized by partially closed trigger loop. The DNA template strand is marked with respect to the putative +1 TSS site. The aspartate triad of the AS coordinates Mg^A^. Conserved residues β/K838 and K846 reach toward the Ap_3_G α phosphate, β/H999 reaches toward the Ap_3_G β phosphate, β/Q567 reaches toward the Ap_3_G γ phosphate and β/Y998 points away from Ap_3_G. The distal adenine base pair with −1T is sandwiched between the *i* site base pair and −2G. Color coding as in Fig. 1. **b**, Cryo-EM density for Ap_3_G (gray) and template (blue). The proximal guanine base in the *i* site WC base pairs with template +1C. The distal adenine base in the *i* *−* 1 site rWC base pairs with template −1T. **c**, AS structure of de novo transcription initiation, where Ap_4_G guanine binds canonically in the *i* site and adenine binds noncanonically in the *i* *−* 1 site. The depiction is analogous to **a**. β′/D739 coordinates both Mg^A^ and Mg^B^. Conserved residues β/K838 and K846 reach toward the Ap_4_G α phosphate, whereas conserved residues β/Q567 and β/H999 reach toward the Ap_4_G γ and δ phosphates; β/Y998 points toward the Ap_4_G δ phosphate. **d**, Cryo-EM density for Ap_4_G (gray) and template (blue). The proximal guanine base in the *i* site WC base pairs with template +1C. The cryo-EM density for the distal part of Ap_4_G is less well defined. The adenine base in the *i* *−* 1 site rWC base pairs with template −1T.

**Extended Data Fig. 9:**
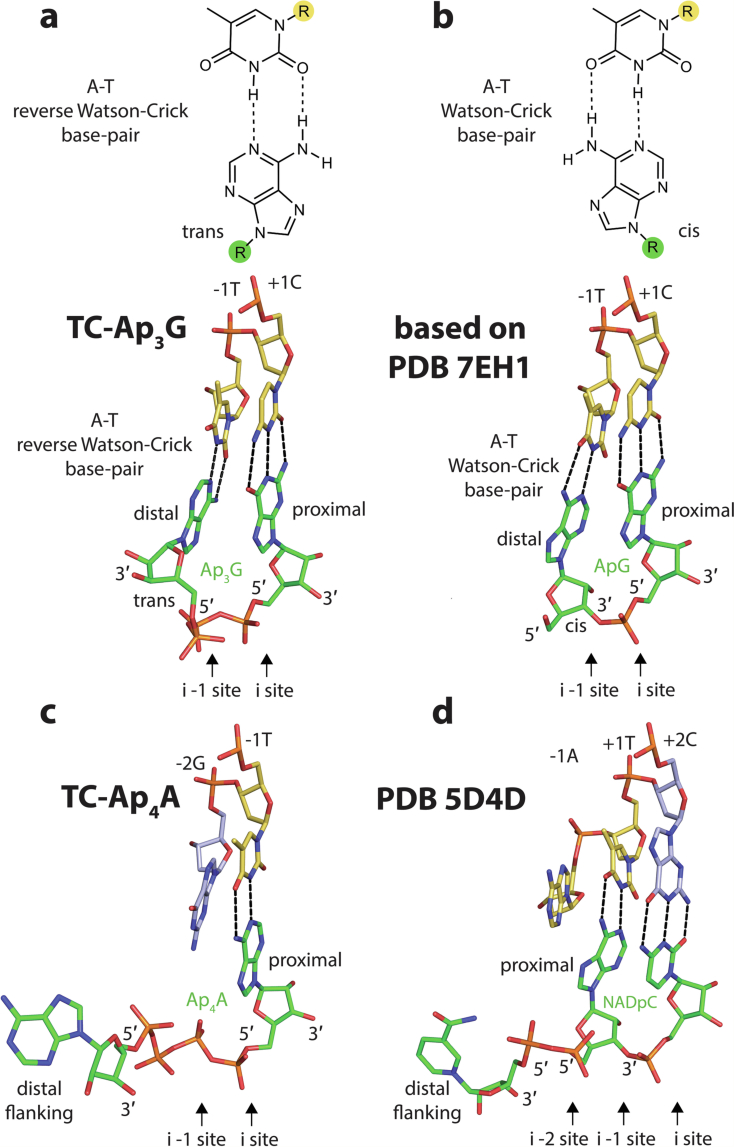
Comparison of WC and rWC base pairing, and NAD binding to RNAP AS. **a**, In TC-Ap_3_G, the glycosidic bond of the distal Ap_3_G adenosine and the glycosidic bond of the template −1 T are in mutual trans orientation, which results in their reverse WC base pairing. **b**, In a regular RNA/DNA duplex, here represented by a 5′-end hydroxyl dinucleotide primer/DNA complex (PDB 7EH1), the glycosidic bond of the distal adenosine in ApG (featuring regular 5′ to 3′ monophosphate linker) and the glycosidic bond of the template −1 T are in mutual cis orientation, which favors canonical WC base pairing. The original CC template and GpG dinucleotide in PDB ID 7EH1 were mutated to TC and ApG for the sake of direct comparison of A-T base pairing in the *i* − 1 site. **c**, In TC-Ap_4_A, the distal Ap_4_A adenosine flanks due to purine-purine base-pairing incompatibility at the *i* − 1 site. **d**, NAD as NCIN visualized after incorporation into nascent product and translocation (PDB 5D4D). The proximal adenosine of NAD base pairs in the *i* − 1 site, while the distal nicotinamide moiety does not base pair with the template strand adenine purine base in line with the *i* − 2 site. A direct comparation of NAD and Np_4_N binding at the *i* and *i* − 1 site of the AS during the very initial step of transcription awaits the determination of a structure with NAD bound in the respective sites.

Next, we visualized the same template TC holoenzyme complex with Ap_3_G and CMPcPP, hereafter referred to as TC-Ap_3_G (Fig. 2a,b, Supplementary Figs. 4b and 7b and Supplementary Table 7). Like in the TC-GTP structure, the CMPcPP in the *i* + 1 site base pairs with the +2G base in a preinsertion position. The guanosine of Ap_3_G in the *i* site base pairs canonically with the +1C TSS (hereafter, we call the nucleoside of Np_*n*_N in the *i* site proximal; Fig. 2b). The guanosine is, thus, aligned for the first nucleotidyl transfer reaction. The α phosphate (counted from the proximal nucleoside) is positioned by β/K838 and β/K846 in a similar way to GTP in the TC-GTP structure. The β phosphate (instead of the γ phosphate in TC-GTP) is positioned by β/H999 and the γ phosphate is positioned by β/Q567; β/Y998 points away from Ap_3_G. There is an interpretable density for Mg^C^ coordinated by the α and γ phosphates of Ap_3_G. The torsion angles between the γ phosphate and the adjacent ribose moiety direct the adenosine into the *i* − 1 position to base pair with the template −1T (hereafter, we call the nucleoside of Np_*n*_N in the *i* − 1 site distal). The glycosidic bond of the distal adenosine is in the *trans* orientation and the adenine base pairs with the −1T base in an rWC manner (Extended Data Fig. 9a). This is in contrast to the *cis* orientation in regular RNA–DNA^27^ or 5′-end hydroxyl dinucleotide primer–DNA duplexes^15^ (Extended Data Fig. 9b). The cryo-EM density (Fig. 2b and Supplementary Fig. 7b) does not support other potential noncanonical base-pairing options such as the Hoogsteen mode. The base pair in the *i* − 1 site is further stabilized by stacking interactions with the base pair in the *i* site from one side and from the other with the template purine base −2G in line with the *i* − 2 site, which overlaps with both the template −1T and the distal adenine base of Ap_3_G. Such stacking stabilization by a purine template base in line with the *i* − 2 site was previously observed for nascent dinucleotide RNA^27^ and 5′-end hydroxyl dinucleotide primers^15^.

In summary, the proximal guanosine in the *i* site binds canonically in the RNAP AS to enable the initial nucleotidyl transfer. The 5′-to-5′ triphosphate linker towards the distal adenosine does not allow for the regular *cis* orientation of the ribose that would enable canonical base pairing with the template; instead, the ribose adopts a *trans* conformation and the distal adenosine base pairs in a rWC manner.

### Ap_4_G tetraphosphate linker allows distal base rWC pairing

In the next step, we aimed to structurally visualize the effect of the tetraphosphate linker of Ap_4_G on binding to the RNAP AS. We reconstituted the template TC holoenzyme complex with Ap_4_G and CMPcPP and obtained a reconstruction hereafter referred to as TC-Ap_4_G (Fig. 2c,d, Supplementary Figs. 4c and 7c and Supplementary Table 7). The CMPcPP and the proximal guanosine of Ap_4_G are canonically bound, aligned for the first nucleotidyl transfer reaction. The α phosphate is canonically positioned by β/K838 and β/K846 and the γ and δ phosphates are in close proximity to β/H999 and β/Q567. Additionally, in contrast to TC-Ap_3_G, residue β/Y998 points toward the δ phosphate group of the Ap_4_G tetraphosphate linker, although a weak map for the alternative conformation pointing away from Ap_4_G is also present. However, the cryo-EM density for the δ phosphate, the distal ribose and the adenine base is less well defined (Fig. 2d and Supplementary Fig. 7c). Nevertheless, the distal ribose clearly adopts the *trans* orientation relative to the template −1T and the distal adenine base pairs in the rWC manner with the −1T base of the template, stabilized further by stacking with the −2G base (Fig. 2c).

Taken together, the results confirm that the proximal moiety of Ap_4_G is aligned for the transcription initiation reaction but the effect of the tetraphosphate linker on the binding of the distal part of the Ap_4_G could not be resolved in detail in the TC-Ap_4_G structure. We, therefore, set out to visualize the tetraphosphate linker and the distal nucleoside binding using Ap_4_A.

### Distal base of Ap_4_A does not require base pairing

**Fig. 3:**
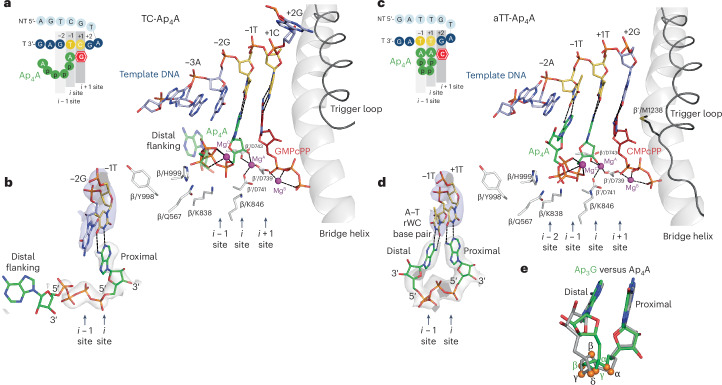
Ap_4_A bound in de novo transcription initiation complexes. **a**, AS structure of de novo transcription initiation, where Ap_4_A proximal adenine binds canonically in the *i* site. GMPcPP is bound in a preinsertion position in the *i* + 1 site. The DNA template strand is antiscrunched by one nucleotide relative to the AS and to the nontemplate strand. The aspartate triad of the AS coordinates Mg^A^ and β′/D739 coordinates Mg^B^. Conserved residues β/K838 and K846 reach toward the Ap_4_A α phosphate and β/H999 and Q567 reach towards the Ap_4_A γ phosphate; β/Y998 points away from Ap_4_A. The distal adenine does not base pair with the −2G base of the template in the *i* *−* 1 site and flanks. Color coding as in Fig. 1. **b**, Cryo-EM density for Ap_4_A (gray) and template (blue). The proximal adenine base in the *i* site WC base pairs with template −1T. The distal adenine base flanks. **c**, AS structure of de novo transcription initiation, where Ap_4_A proximal adenine binds canonically in the *i* site and the distal adenine binds noncanonically in the *i* *−* 1 site. The depiction is analogous to **a**. Conserved residues β/K838 and K846 reach toward the Ap_4_A α phosphate, H999 reaches toward the Ap_4_A γ phosphate and β/Q567 reaches toward both γ and δ phosphates of the Ap_4_A; β/Y998 points away from Ap_4_A. **d**, Cryo-EM density for Ap_4_A (gray) and template (blue). The proximal adenine base in the *i* site WC base pairs with template +1T. The distal adenine base in the *i* *−* 1 site rWC base pairs with template −1T. **e**, Comparison of triphosphate and tetraphosphate linkers in TC-Ap_3_G (green) and aTT-Ap_4_A (gray) structures. Phosphorus atoms are highlighted as orange spheres. Nonbridging oxygen atoms of phosphates are omitted for clarity.

The transcription reaction initiating with Ap_4_A on the TC template yielded a capped product (Fig. 1c,d) starting from the −1T template position as the TSS, instead of the putative +1C TSS position, confirmed by LC–MS (Extended Data Fig. 2 and Supplementary Table 3). Reconstitution of the TC template holoenzyme complex with Ap_4_A and GMPcPP yielded a reconstruction hereafter referred to as TC-Ap_4_A (Fig. 3a,b, Supplementary Figs. 4d and 7d and Supplementary Table 7). In this structure, indeed, the template strand is antiscrunched^31–33,39^ by one nucleotide, like in the TC-empty structure (Extended Data Fig. 8c), placing the template −2G base in line with the *i* − 1 site, the template −1T base in line with the *i* site and the template +1C base in line with the *i* + 1 site (Supplementary Fig. 8d,e). The GMPcPP and the proximal adenosine of Ap_4_A are canonically paired in the *i* + 1 site with the +1C base and in the *i* site with the −1T base, respectively (Fig. 3a and Supplementary Fig. 7d). The distal adenosine of Ap_4_A does not base pair with the bulky −2G purine residue in the *i* − 1 site and is, therefore, flanking into the void of the AS cavity toward σ^A^ region 3.2. There is only a blurred density for the distal ribose (Fig. 3b) and no defined density for the distal base, suggesting that there is no contact with σ^A^ region 3.2. The base pair in the *i* site is stabilized by a stacking interaction with the −2G base in line with the *i* − 1 site. This type of stacking has already been reported to occur during stabilization of the first initiation NTP (iNTP) binding in the initiation complex^37^. Similarly, the interaction of the Ap_4_A proximal base is sufficient to retain it in the AS, even without additional stabilization from pairing of the distal nucleoside base of Ap_4_A at the *i* − 1 site. The tetraphosphate linker is stabilized by the canonical interaction of α and γ phosphates with conserved residues β/K838 and K846, and β/H999 and Q567, respectively. The mutual orientation of the phosphates is also stabilized by the coordination of Mg^C^, engaging all four phosphates.

In summary, the inherent capability of the TC template strand to antiscrunch in the AS by one nucleotide enabled the Ap_4_A to canonically bind in the *i* site. The purine incompatibility at the *i* − 1 site, however, forces the distal base of Ap_4_A to flank. Nevertheless, such an Ap_4_A initiation complex is stable enough for the transcription reaction to proceed.

### Ap_4_A can base pair using both proximal and distal nucleosides

To visualize the pairing of Ap_4_A nucleosides in both *i* and *i* − 1 sites, we attempted to reconstruct the holoenzyme complex with Ap_4_A, GMPcPP and a modified template tTC (Extended Data Table 1) that would place the T base in line with the *i* − 1 site after template antiscrunching. However, even though the template was positioned as expected, we could only visualize blurred contours of Ap_4_A and GMPcPP in the AS. We, therefore, created the aTT template (Extended Data Table 1 and Supplementary Fig. 4e), which featured a transcription bubble shortened by one base to prevent antiscrunching and was still compatible with IVT reactions (Supplementary Fig. 9). We expected that this template would position the +1T and −1T bases in line with the *i* and *i* − 1 sites, respectively, to achieve full base pairing with Ap_4_A; the +2G base would be in line with the *i* + 1 site to base pair with CMPcPP. The resulting structure, hereafter referred to as aTT-Ap_4_A (Fig. 3c,d, Supplementary Fig. 7e and Supplementary Table 7), indeed visualized CMPcPP and the proximal adenosine of Ap_4_A canonically bound in the *i* + 1 and *i* sites, respectively (Fig. 3c). The cryo-EM density was clearly defined for the tetraphosphate linker and for the distal adenosine base pairing with the −1T base of the template in the *i* − 1 site (Fig. 3d and Supplementary Fig. 7e). The α phosphate is canonically positioned by β/K838 and β/K846. Residue β/H999 reaches toward the γ phosphate, which itself interacts with β/Q567. The cryo-EM map for residue β/Y998 indicates two alternative conformations, pointing away and toward Ap_4_A; however, the conformation pointing away from Ap_4_A prevails. Concomitantly, the δ phosphate of Ap_4_A in aTT-Ap_4_A adopts a different position in comparison to Ap_4_G in TC-Ap_4_G. The β and α phosphates together coordinate Mg^C^. The relatively loosely defined distal ribose adopts a *trans* orientation relative to the −1T template base, with the distal adenine forming an rWC pair with −1T, as supported by the cryo-EM map (Fig. 3d and Supplementary Fig. 7e). The distal adenine base is also stabilized in the base pair with −1T by stacking interactions with the −2A purine base of the template in line with the *i* − 2 site. The distal adenine binds similarly to the −1T template base in both TC-Ap_3_G and aTT-Ap_4_A (Fig. 3e; root mean square deviation of the distal base = 0.421 Å). Intriguingly, it appears that the binding conformations of the two bases in Np_*n*_Ns are constant and that it is the conformation of the triphosphate or tetraphosphate linker that needs to adjust in between proximal and distal nucleosides.

In all our structures, when base pairing, the distal adenosine in the *i* − 1 site features ribose in the *trans* orientation relative to the template in line with the *i* − 1 site, which forces the distal adenosine to rWC base pair with the template base in line with the *i* − 1 site. We, therefore, conclude that the rWC mode is the preferred binding mode of the distal adenosine, induced by the spatial constrains of the 5′-to-5′ triphosphate or tetraphosphate linker between the ribose of the proximal nucleoside and that of the distal nucleoside.

## Discussion

In this article, we describe the molecular details of 5′ RNA capping with Np_*n*_Ns by bacterial RNAP. We confirm previous observations^11,19^ that Np_*n*_Ns are used as NCINs in IVT reactions using bacterial RNAP (in our case, *Tt* RNAP). As anticipated, Np_*n*_Ns readily initiate with the proximal base at the putative +1 TSS of a DNA promoter when canonical base pairing is available. In our in vitro system, using artificial promoters with preformed transcription bubbles (Fig. 1b and Extended Data Table 1), the template strand TSS can oscillate between the +1 and −1 positions (Supplementary Fig. 4a, Extended Data Fig. 8 and Supplementary Fig. 8). When the putative +1 TSS is not compatible with canonical base pairing with the proximal base of Np_*n*_Ns, the −1 position, where canonical base pairing is possible, is readily used as the TSS instead. This leads to the formation of capped RNA longer by one nucleotide. We observe this TSS shift, for example, with Ap_3-4_A initiating at the −1T position of the TC template (Fig. 1d) as confirmed by the detection of the cap(−1)TSS-RNA product in our LC–MS analyses (Extended Data Fig. 2 and Supplementary Table 3). Furthermore, we observed the TSS shift for other combinations of Np_*n*_Ns and templates (Extended Data Figs. 4–7 and Supplementary Tables 5 and 6), demonstrating that this is a general behavior of this particular set of artificial DNA promoters with preformed transcription bubbles. As only limited information^19^ is available on natural RNA sequences capped by Np_*n*_Ns, it is difficult to study this behavior in the context of native DNA promoters. To assess Np_*n*_N capping under more native-like conditions, we developed an IVT assay using a supercoiled plasmid with a fully complementary DNA promoter. PAGE and LC–MS analyses combined with RNase A treatment confirmed that *Tt* RNAP generates all Np_*n*_N-capped RNAs from this standard template, initiating transcription from both +1 and −1 positions as TSSs (Extended Data Fig. 3 and Supplementary Table 4).

Next, to characterize Np_*n*_Ns as NCINs, we visualized the initial binding of Np_*n*_Ns into the RNAP AS just before the first nucleotidyl transfer reaction. In a series of cryo-EM structures, we captured the RNAP AS, where NCINs align to react with the first extending nucleotide. The structures reveal how Np_*n*_Ns bind to the *i* and *i* − 1 sites of the RNAP AS and how they base pair with the promoter template strand. Expectedly, the proximal G or A of either Ap_*n*_G or Ap_*n*_A base pairs with the template in line with the *i* site in a canonical WC manner to align with the RNAP AS to react with the extending nucleotide. The distal nucleoside either base pairs in line with the *i* − 1 site or flanks into the AS cavity. Importantly, the base pairing of the distal base is not canonical because of the presence of the 5′-to-5′ triphosphate or tetraphosphate linker between the two nucleosides, instead of a 5′-to-3′ single phosphate in a regular RNA product^15^ (Extended Data Figs. 1 and 9). The orientation of the distal and proximal ribose of the Np_*n*_Ns resulting from the 5′-to-5′ linkage does not allow the canonical *cis* conformation of the distal ribose aligned with the *i* − 1 site but instead dictates a *trans* conformation. The *trans* conformation is then compatible with the rWC base pairing of the distal nucleoside. The rWC-pairing distal bases in both TC-Ap_3_G and aTT-Ap_4_A adopt similar binding poses (Fig. 3e), likely facilitated by conformational adjustment of the flexible triphosphate or tetraphosphate linker between the proximal and distal nucleosides. In addition, we also conclude that, when there is a bulky purine template base in line with the *i* − 1 site, such as the −2G base in the antiscrunched template TC, the distal purine nucleoside of Np_4_N does not form any noncanonical purine–purine base pair because of steric hindrance and is forced to flank. In other words, two bulky purines cannot be accommodated opposite each other.

One of the best known noncanonical RNA caps is NAD, which has also been detected in various bacterial RNAs^3,18,40,41^. However, the incorporation efficiency of NAD as an NCIN is only about one seventh of that of ATP^19^. Nevertheless, we wanted to compare the incorporation efficiency of NAD by *Tt* RNAP with Np_*n*_Ns. Expectedly, our efforts to use NAD as NCIN produced only traces of NAD-RNA (Supplementary Fig. 3) and these experiments confirmed that NAD is indeed a much less potent NCIN than NTPs and Np_*n*_Ns. Our attempts to visualize NAD binding to the *i* and *i* − 1 sites of the RNAP AS by cryo-EM have failed. Therefore, we can only compare Np_*n*_N binding at the *i* and *i* − 1 sites to NAD binding at the *i* − 1 and *i* − 2 sites, as visualized in the RNAP complex with NAD-pC trinucleotide (PDB 5D4D; Extended Data Fig. 9d).

The *Tt* RNAP is a well-established representative of the RNAP AS, conserved across all cellular RNAPs^37,42^; therefore, it can be used as a model to study interactions of Np_*n*_Ns with the RNAP itself. However, apart from the conserved residues β/K838, K846, H999 and Q567, which contact the phosphate groups of both the canonical iNTPs and Np_*n*_Ns as NCINs, we only identified β/Y998 as a potential additional interacting residue (Fig. 2c and Extended Data Fig. 8a). The alternative conformation of β/Y998 pointing toward the NCIN might potentially interact with the δ phosphate group in the case of the tetraphosphate linker, as observed in the TC-Ap_4_G structure. However, given the low occupancy of this β/Y998 conformer in the structures with Ap_4_A, it does not seem to be critical for tetraphosphate linker binding into the RNAP AS. Surprisingly, we observed an Mg^C^ cation coordinated by the triphosphate or tetraphosphate of iNTPs or Np_*n*_Ns. Mg^C^ does not specifically interact with the RNAP protein and we propose that it serves as a countercharge to the phosphate groups.

The only determinant of how Np_*n*_Ns bind in the RNAP AS seems to be the DNA promoter sequence at the +1 and −1 template positions aligned in the *i* and *i* − 1 sites, respectively. A strict requirement is canonical WC compatibility in the *i* site. In our in vitro system, we observe that the template strand can be antiscrunched, thereby shifting the TSS by one position (Extended Data Fig. 8c and Supplementary Figs. 4a and 8). Intriguingly, in the case of the TC-Ap_4_A structure (Supplementary Fig. 4d), the base identity of Ap_4_A favored the antiscrunched TSS template strand position to canonically bind in the *i* site. As Np_*n*_Ns have two 3′ hydroxyl groups available for initiation on both termini of the molecule, Ap_*n*_Gs use both termini in our IVT reactions. The selection of the proximal base (A or G) in the *i* site follows the base identity requirements in the TSS.

The incorporation of Np_*n*_Ns as NCINs increases the efficiency of transcription initiation in comparison to regular iNTPs^19,20^. Furthermore, template pyrimidine bases at the *i* − 1 site increase the efficiency of Np_*n*_Ns noncanonical transcription initiation, one of the explanations being potential WC base pairing^19^. Our structure analysis revealed rWC base pairing of the T pyrimidine base at the *i* − 1 site. Nevertheless, transcription initiation with template TC and Ap_4_A is efficient (Fig. 1d) despite the lack of base pairing at the *i* − 1 site with the bulky purine −2G (Fig. 3a,b). On the other hand, −2G stabilizes the proximal base pair by a stacking interaction. Our structural data on the very first step of transcription initiation, thus, do not provide any clear mechanistic explanation of the observed^19^ increase in transcription initiation efficiency caused by pyrimidine bases aligned with the *i* − 1 site. Future structural studies of subsequent transcription steps might provide a deeper understanding.

In summary, we present molecular details of how Np_*n*_Ns bind into the AS of RNAP at the very beginning of transcription, which demonstrate how Np_*n*_Ns function as versatile and efficient NCIN. Given the conservation of the Np_*n*_N-binding region in the RNAP AS, we presume that the here-described modes of Np_*n*_Ns binding will be found to be universal for other cellular RNAPs, including eukaryotic polymerase II.

## Methods

Unless mentioned otherwise in the text, the chemicals used were purchased from Merck chemicals. If available, the chemicals were of molecular biology grade. Oligonucleotides were purchased from Generi Biotech. DNA scaffolds were prepared using two oligonucleotides (template strand and nontemplate strand) and annealed in a total volume of 240 μl containing 33 μM each oligonucleotide, 10 mM Tris pH 7.8, 50 mM NaCl and 1 mM EDTA. Samples were heated to 90 °C for 5 min, after which the temperature gradually decreased to 20 °C in 2 h. All templates are listed in Supplementary Table 1.

### *Tt* RNAP holoenzyme expression and purification

As previously described^26^, purification of the RNAP holoenzyme poses difficulties caused by its unstable σ^A^ subunit that is susceptible to proteolysis. To prevent sample heterogeneity, we isolated the native RNAP core from *T. thermophilus* and complexed it with a recombinantly expressed σ^A^ subunit to obtain a homogeneous preparation of the RNAP holoenzyme.

For isolation of the RNAP core, *Tt* HB8 (DSM579, German Collection of Microorganisms and Cell Cultures) cells were grown at 75 °C in a medium containing 4 g l^−1^ yeast extract, 8 g l^−1^ proteose peptone no. 3 and 2 g l^−1^ NaCl at pH 7.0. Cells were harvested after 20 h of cultivation, resuspended in lysis buffer (20 mM Tris-HCl pH 8.7, 50 mM NaCl, 10 mM EDTA, 10 mM β-mercaptoethanol and 0.1 mM PMSF), disrupted using an EmulsiFlex-C3 cell disrupter (Avestin) and centrifuged at 20,000*g*. The resulting cell lysate was applied to a Q-Sepharose high-performance column (Cytiva) equilibrated in lysis buffer. The column was washed with lysis buffer and eluted with a seven-step gradient of sodium chloride (0.15–1 M). Fractions containing the RNAP core were collected and concentrated using Amicon Ultra centrifugal units (30-kDa molecular weight cutoff (MWCO); Merck Life Sciences). This step is crucial because it facilitates the removal of most of the DNA from the sample, allowing it to efficiently bind to the Mono-Q column in the next step. Each sample was then dialyzed into buffer A (20 mM Tris-HCl pH 8.7, 50 mM NaCl, 5 mM β-mercaptoethanol and 1 mM EDTA) and loaded onto a Mono-Q 5/50 GL column (Cytiva) equilibrated in buffer A. The column was washed with buffer A and eluted with a linear gradient of sodium chloride (0.05–1 M). Each sample was subsequently concentrated and loaded onto a Superdex 200 10/300 GL column (Cytiva) with a running buffer containing 25 mM Tris-HCl pH 8.7, 200 mM NaCl and 5% glycerol. Fractions containing the RNAP core were concentrated to 1 mg ml^−1^ and stored at −80 °C.

The σ^A^ subunit synthetic gene was subcloned into the pMCSG7 vector (T7 promoter-driven, originally designed for ligation-independent cloning^43^). The vector was first modified by a sequence coding for the N-terminal His_6_ tag, followed by a sequence coding for the tobacco etch virus protease cleavage site with additional amino acid residues (SNAAS). The σ^A^ subunit coding sequence was cloned as an NHeI–EcoRI insert into this modified vector. The protein was overexpressed in the *E*. *coli* strain BL21 (DE3) (New England Biolabs (NEB)) at 37 °C in LB medium supplemented with 0.8% glycerol and 100 μg ml^−1^ ampicillin. Subsequently, σ^A^ expression was induced at an optical density at 600 nm of 0.6 by addition of ETG to the final concentration of 1 mM and cells were further cultivated for 3 h before they were harvested by centrifugation. Cells were resuspended in ten volumes of lysis buffer (50 mM Tris-HCl pH 7.9, 200 mM NaCl, 10 mM β-mercaptoethanol and 5% glycerol) containing protease inhibitors (cOmplete EDTA-free, Roche) and lysed using an EmulsiFlex-C3 cell disrupter (Avestin). The cell lysate was clarified by centrifugation at 20,000*g* and loaded onto a 5-ml HisTrap Ni-NTA column equilibrated in lysis buffer. The column was washed with lysis buffer supplemented with 10 mM imidazole and the protein was eluted in three steps with lysis buffer containing 50, 250 and 500 mM imidazole. Fractions containing the σ^A^ subunit were collected and concentrated using Amicon Ultra centrifugal units (30-kDa MWCO; Merck Life Sciences). The last purification step involved gel filtration on a Superdex 200 10/300 GL column (Cytiva) in a running buffer containing 50 mM Tris-HCl pH 7.9, 200 mM NaCl, 10 mM β-mercaptoethanol and 5% glycerol. The purified σ^A^ protein was concentrated to 13 mg ml^−1^ and stored at −80 °C. The purification yield was 7.8 mg of protein from 1 l of bacterial culture with the purity assessed by silver-stained SDS–PAGE to be greater than 95%.

To prepare the holoenzyme complex, the σ^A^ subunit was transferred into a buffer containing 20 mM Tris-HCl pH 8.7, 100 mM NaCl and 5% β-mercaptoethanol, mixed with the RNAP core in a 4:1 molar ratio and incubated overnight at 4 °C. The mixture was then applied to a Superdex 200 10/300 GL column (Cytiva) equilibrated in 20 mM Tris-HCl pH 8.7, 100 mM NaCl and 1% glycerol. In this step, the excess of the σ^A^ subunit was separated from the RNAP holoenzyme, as verified by SDS–PAGE analysis (Supplementary Fig. 10). Fractions containing the RNAP holoenzyme were pooled, concentrated to a final concentration of 1 mg ml^−1^ using Amicon Ultra centrifugal units (30-kDa MWCO, Merck Life Sciences) and stored at −80 °C.

### IVT with *Tt* RNAP

IVT was performed in a reaction volume of 25 μl containing 2 μM template DNA (Supplementary Table 1), 0.6 mM ATP, 0.6 mM GTP, 0.6 mM UTP, 0.4 mM CTP and 0.2 μl of α-[^32^P]CTP (activity: 9.25 MBq in 25 μl), 1.6 mM Np_*n*_Ns (Ap_3_A, Ap_4_A, Ap_3_G, Ap_4_G, Gp_3_G and Gp_4_G; Jena Bioscience) or NAD in different concentrations (0.6 mM, 1.6 mM, 3.2 mM and 6.4 mM), 50 mM Tris-HCl pH 7.9, 100 mM KCl, 10 mM MgCl_2_, 1 mM DTT, 5 μg ml^−1^ BSA, 5% glycerol and 96 nM *Tt* RNAP. In negative controls, Np_*n*_Ns were replaced with appropriate regular NTPs ATP and/or GTP (Supplementary Table 2). The concentration of ATP was 2.2 mM in the negative controls for the samples containing Ap_*n*_A as starting nucleotides. The concentration of GTP was 2.2 mM in the negative controls for the samples containing Gp_*n*_G as starting nucleotides. The concentration of ATP and GTP was 1.4 mM each in the negative controls for the samples containing Ap_*n*_G as starting nucleotides (Supplementary Table 2). The IVT mixture was incubated for 2 h at 65 °C.

### DNAse treatment of in vitro transcripts

To obtain pure RNA, the DNA template was removed by DNase I digestion. A total of 25 μl of the transcription mixture was mixed with 3 μl of 10× reaction buffer for DNase I (10 mM Tris-HCl pH 7.6 at 25 °C, 2.5 mM MgCl_2_ and 0.5 mM CaCl_2_, supplied with the enzyme) and 4 U of DNase I (NEB) and incubated at 37 °C for 60 min. The enzyme was heat-deactivated at 75 °C for 10 min and then immediately cooled on ice. All samples were purified using size-exclusion columns (Micro Bio-Spin P-6 gel columns, Biorad).

### RNA 5′-polyphosphatase treatment of uncapped RNA transcripts

To obtain monophosphate RNA, uncapped RNA was treated with 20 U of 5′-polyphosphatase (Lucigen) in a solution of 1× buffer (supplied with the enzyme) in a total volume of 25 μl for 1 h at 37 °C. Samples were purified using size-exclusion columns (Micro Bio-Spin P-6 gel columns, Biorad).

### Radiolabeling of RNA markers

The RNA markers having a sequence complementary to that of template TC and template CT (starting at the −1 and +1 position) and gTT (starting at the +1 position) were purchased from Eurofins genomics. The radiolabeling was performed in a volume of 20 μl using 0.6 μl of T4 polynucleotide kinase (NEB), 2 μl of T4 polynucleotide kinase reaction buffer (70 mM Tris-HCl pH 7.6 at 25 °C, 10 mM MgCl_2_ and 5 mM DTT, supplied with the enzyme), 4.55 μl α-[^32^P]ATP (activity: 9.25 MBq in 25 μl) and 0.75 μM RNA. The reaction mixture was incubated at 30 °C for 30 min and the enzyme was heat-deactivated at 65 °C for 20 min. The samples were purified using size-exclusion columns (Micro Bio-Spin P-6 gel columns, Biorad).

### PAGE analysis of in vitro transcripts

Samples (10 μl) were mixed with 10 μl of 2× RNA loading dye (NEB), incubated at 75 °C for 5 min and then cooled on ice. Samples were loaded onto 12.5% polyacrylamide gels (with or without the addition of acryloylaminophenyl boronic acid (APB)) and electrophoretic separation was performed under denaturing conditions at 600 V for 3 h using 1× TBE as a running buffer. Denaturing PAGE gels were visualized by a Typhoon FLA 9500 imaging system and analyzed with ImageJ 1.53e software.

### IVT for LC–MS analysis

IVT was performed in a volume of 25 μl containing 2 μM template DNA (Supplementary Table 1), 0.6 mM ATP, 0.6 mM GTP, 0.6 mM CTP, 1.6 mM Np_*n*_Ns, 5 mM Tris-HCl pH 7.9, 10 mM KCl, 1 mM MgCl_2_, 0.1 mM DTT and 96 nM *Tt* RNAP. The mixture was incubated for 2 h at 65 °C. All samples were purified using MWCO filters (Sartorius, Vivacon 500, 10-kDa MWCO HY). The filters were prewashed once with 50 µl of the reaction buffer and then the samples diluted with 20 µl of molecular-biology-grade water were added and centrifuged at 13,000*g* for 15 min at room temperature. Samples were then analyzed by hydrophilic interaction liquid chromatography (HILIC) with MS detection.

### LC–MS analysis of IVT products

RNA products were diluted tenfold with 50 mM ammonium acetate (pH 7.0). For the analysis, a high-performance LC instrument (Acquity H-class, Waters) equipped with an Xbridge Premier BEH amide column (2.5 µm and 4.6 mm × 150 mm; Waters) was used. The column temperature was 35 °C. Mobile phase A contained 20 mM ammonium acetate (Fisher) in a 90:10 v/v mixture of acetonitrile (Optima, Fisher) with ultrapure water (18.2 MΩ cm; Purelab Chorus system, Elga). Mobile phase B contained 20 mM ammonium acetate in ultrapure water. The autosampler was kept at 10 °C. The injection volume was 5 µl. The gradient of separation is shown in Supplementary Table 9. MS detection was performed on a Xevo G2-XS quadrupole time-of-flight (Q-TOF) MS instrument (Waters) equipped with an electrospray ionization source with parameters detailed in Supplementary Table 10. Fragmentation spectra of mass selected ions were generated with increased collision energy (30 eV). LC–MS data were acquired and analyzed with MassLynx version 4.2 software and graphs were prepared with GraphPad Prism 10.

### TC plasmid preparation

The plasmid pRLG7558 containing p770 promoter driving the *veg* RNA expression and ribosomal RNA B (*rrnB*) under the P1 promoter^36^ was a gift from the L. Krasny laboratory. In this plasmid, we substituted the *veg* promoter with our TC promoter sequence below using the EcoRI and HindIII restriction sites. The inserts were generated by annealing the following 5′-monophosphorylated oligonucleotides: forward: (5′-AATTCTCTTGACATAATCCATATGGTTGGGTATAATGGGAG**AG**-3′); reverse: (5′-AGCT**CT**CTCCCATTATACCCAACCATATGGATTATGTCAAGAG-3′)

The −1 and +1 positions in both strands are in bold.

The resulting constructs were verified by DNA sequencing. Plasmid DNA was purified using the PureLink HiPure plasmid midiprep kit (ThermoFisher Scientific), further extracted using phenol–chloroform purification and dissolved in pure water.

### IVT with the TC plasmid

IVT was performed in a reaction volume of 25 μl containing 10 ng μl^−1^ TC plasmid (Supplementary Table 1), 1.6 mM Np_*n*_Ns (Ap_3_A, Ap_4_A, Ap_3_G, Ap_4_G, Gp_3_G and Gp_4_G; Jena Bioscience) and 1 μl of RNase Inhibitor (NEB), in the presence of reaction buffer containing 50 mM Tris-HCl (pH 7.9), 100 mM KCl, 10 mM MgCl_2_, 1 mM DTT, 5 μg ml^−1^ BSA and 5% (v/v) glycerol. For reactions with *Tt* RNAP, 0.15 μM σ^A^ subunit and 0.05 μM *Tt* RNAP were added. For the reaction with *E*. *coli* RNAP, the supplied 1× reaction buffer (NEB) and 1 μl of *E. coli* RNAP holoenzyme (NEB) were used instead. A separated nucleotide mixture was prepared containing 0.6 mM ATP, 0.6 mM GTP, 0.6 mM UTP, 0.4 mM CTP and 0.3 μl of α-[^32^P]CTP (activity: 9.25 MBq in 25 μl). Reaction mixtures without NTPs were preincubated for 10 min at 65 °C (*Tt* RNAP) or at 37 °C (*E*. *coli* RNAP), followed by addition of the NTP mix. Complete IVT reactions were then incubated for 2 h at 65 °C (*Tt* RNAP) or at 37 °C (*E*. *coli* RNAP). In negative control reactions, Np_*n*_Ns were replaced with GTP to observe only the +1 starting uncapped RNA. Following incubation, the samples were purified using size-exclusion columns (Micro Bio-Spin P-6 gel columns, Biorad).

### PAGE analysis of IVT products from the TC plasmid

Samples (10 μl) were mixed with 10 μl of 2× RNA loading dye (NEB) and incubated at 90 °C for 5 min and then cooled on ice. Samples were loaded onto 8% polyacrylamide gels (with the addition of APB) and electrophoretic separation was performed under denaturing conditions at 600 V for 4 h using 1× TBE as a running buffer. Denaturing PAGE gels were visualized by a Typhoon FLA 9500 imaging system.

### IVT with the TC plasmid for LC–MS analysis

IVT was performed as described above, with modified enzyme concentrations. Specifically, 0.3 μM σ^A^ subunit and 0.1 μM *Tt* RNAP were used in each 25-µl reaction. Following incubation, RNA samples were purified using RNA mini Quick Spin columns (Merck), eluting in 15 µl before LC–MS analysis.

### Digestion of IVT products from the TC plasmid by RNase A

The RNA samples (15 µl) were mixed with 2.5 µl of ammonium acetate (500 mM, pH 7.5), 2.5 µl of EDTA (1 mM) and 5 µl of RNase A (200 ng µl^−1^, NEB). RNase A specifically degrades single-stranded RNA after C and U residues leaving a phosphate at the 3′ end. The reaction was kept for 30 min at 37 °C. Right after, the reaction was transferred to an HPLC vial and directly measured by ion-pairing reverse-phase chromatography with MS detection.

### LC–MS analysis of IVT products from the TC plasmid

Digested RNA products were separated on Acquity I-class (Waters) equipped with Acquity Premier oligonucleotide BEH C18 column (1.7 µm, 2.1 mm × 50 mm; Waters). The column temperature was 35 °C. Mobile phase A contained 15 mM triethylamine (Fisher) and 400 mM 1,1,1,3,3,3-hexafluoro-2-propanol (HFIP) in ultrapure water (18.2 MΩ cm; Purelab Chorus system, Elga). Mobile phase B contained 15 mM triethylamine and 400 mM HFIP in methanol (Optima, Fisher). The autosampler was kept at 10 °C. The injection volume was 10 µl. The gradient of separation is shown in Supplementary Table 11. MS detection was performed with the same Xevo G2-XS Q-TOF MS instrument. Ionization parameters are detailed in Supplementary Table 12.

### Ap_4_G purification for the cryo-EM study

To prevent contamination of the Ap_4_G standard by pppApG, we treated Ap_4_G and pppApG standard (used as a control, Jena Bioscience) with 1 U of nuclease P1 (NEB) in 1× buffer (50 mM ammonium acetate, pH 5.3) for 30 min at 37 °C followed by treatment with 0.03 U of shrimp alkaline phosphatase (NEB) in 1× rCutSmart buffer (50 mM potassium acetate, 20 mM Tris acetate, 10 mM magnesium acetate and 100 µg ml^−1^ recombinant albumin, pH 7.9 at 25 °C) and incubated for 10 min at 37 °C. The samples were purified using MWCO filters (Sartorius, Vivacon 500, 10-kDa MWCO HY). The filters were washed once with 50 µl of the reaction buffer and then samples, diluted with 20 µl of molecular-biology-grade water, were added and centrifuged at 10,000*g* for 10 min at room temperature. The samples were dried on a Speedvac system and dissolved in 50 μl of 50 mM ammonium acetate, pH 7.0. The filtrate was analyzed by LC–MS using the same method described above.

### Cryo-EM grid preparation

In vitro reconstitution was performed in 30 μl of 50 mM Tris-HCl pH 7.9, 100 mM KCl, 10 mM MgCl_2_, 1 mM DTT, 2.6 μM DNA template, 3.3 mM GTP or 1.6 mM Np_*n*_Ns, 1.6 mM CMPcPP or GMPcPP (Jena Bioscience), 1.3 μM *Tt* RNAP σ^A^ holoenzyme and an additional 1.5 μM σ^A^. Each sample was incubated for ~10 min at 4 °C. Sample aliquots of 3 μl were applied to glow-discharged Quantifoil R2/1 Au 300-mesh grids, immediately blotted for 2 s and plunged into liquid ethane using a Thermo Fisher Scientific Vitrobot Mark IV (4 °C, 100% humidity).

### Cryo-EM data collection

The grids were loaded into a 300-kV Titan Krios (FEI) electron microscope equipped with a Gatan K3 (model 1025) direct electron detector mounted on a Gatan BioQuantum (model 1967) energy filter. Data were collected using Serial EM software^44^ in image shift acquisition mode (3 × 3 holes; 7–8 exposures per hole) at a nominal magnification of ×105,000 with a pixel size of 0.8336 Å per pixel. Videos were collected for 2–2.7 s at a flux of 15–20 electrons per Å^2^ per s, giving a total exposure of around 40–50 electrons per Å^2^. The defocus values ranged from −0.5 to −3.0 μm. In total, 40 frames of each video were saved, except aTT-Ap_4_A, for which 46 frames were saved.

### Cryo-EM image processing

All data processing (Supplementary Figs. 5 and 6 and Supplementary Tables 7 and 8) was performed using the RELION 4.0 software package^45^. Motion correction was performed using the RELION implementation of MotionCor2 (ref. ^46^). Videos were aligned using 7 × 5 patches with dose weighting. Contrast transfer function (CTF) was estimated using CTFFIND4.1 (ref. ^47^) from summed power spectra^48^ for every four electrons per Å^2^. From each dataset, 25 micrographs were randomly selected and a representative set of particles was picked manually. These particles, along with their coordinates, were pooled for the training of a consensus Topaz picking model^49^. Particles were subsequently picked from individual datasets by Topaz using this consensus-trained model.

After initial binning, particles underwent three rounds of two-dimensional classification. In each round, the particles were sorted into 200 classes with an *E*-step of 8 Å and a mask diameter of 240 Å. Only classes with well-defined structural features were retained and subjected to three-dimensional (3D) classification using a reference from the *Tt* RNAP crystal structure (PDB 4Q4Z)^37^. The first 3D classification sorted particles into ten classes with the regularization parameter set to *T* = 4 and an *E*-step of 5 Å. Quantitative analysis of the individual class types is summarized in Supplementary Table 8. Selected classes were aligned into a global 3D refinement. A subsequent 3D classification, using the result of the previous 3D refinement as input along with the corresponding mask, was performed using local searches from 3.7° to 1.8° with the regularization parameter increased to *T* = 8. Particles with poorly defined structural features were removed and the remaining particles were reextracted to native pixel size. Particles were refined globally again and corrected for aberrations, Bayesian-polished and 3D refined. The result of the refinement was used as input for focused 3D classification with masks around the broad core of the RNAP, performed at a local search interval of 0.5°, and the regularization parameter was increased to *T* = 200. Particles with poorly defined structural features for bound DNA were removed and the remaining particles were pooled and 3D refined. For TC-Ap_4_G and aTT-Ap_4_A, another round of focused 3D classification with masks around the RNAP AS was performed without angular searches. Classes were selected on the basis of features of the RNAP AS and pooled for the final 3D refinement. The final cryo-EM density maps were generated by the postprocessing feature in RELION and sharpened or blurred into MTZ format using CCP-EM^50^. The final set consisted of sharpened or blurred MTZ maps with *B* = −200, −100, −50, 0, 50, 100 and 200 Å^2^. The resolution of the cryo-EM density maps (Supplementary Table 7 and Supplementary Fig. 6d) was estimated with the gold-standard Fourier shell correlation cutoff value of 0.143. Reference-based local amplitude scaling was performed by LocScale^51^. The angular orientation distribution of the 3D reconstruction was calculated by cryoEF (version 1.1.0)^52^. Local resolution was calculated within RELION 4.0.

### Cryo-EM model building and refinement

The TC-Ap_3_G model was built as follows. The X-ray structure of the *Tt* RNAP transcription initiation complex (PDB 4Q4Z)^37^ was used as a starting model and docked into the cryo-EM map by Molrep^53^. The model was rebuilt manually in Coot (version 0.9.8.92)^54^ against a blurred MTZ map (*B* = 50 Å^2^) generated in CCP-EM^55^. Model self-restraints were used, as well as base pairing and parallelity restraints for DNA, Ap_3_G and CMPcPP, which were automatically generated by the program libG^56^ running under Refmac (version 5.8.0405)^57^ within the CCP4 Interface (version 8.0.010)^58^ and curated manually. The model was refined in real space^59^ against a postprocessed MRC map in PHENIX (version 1.21-5207)^60^, using self-restraints with the strict rotamer matching option enabled, as well as secondary-structure restraints, including base pairing and parallelity restraints for DNA, Ap_3_G and CMPcPP. The restraints were generated automatically in PHENIX (version 1.21-5207)^60^ and edited manually. In general, parallelity was maintained among adjacent bases of the Np_*n*_Ns, the extending nucleotide analogs and the nonpairing template base adjacent to the distal pairing Np_*n*_N base. Distances and weights for the base-pairing hydrogen bonds were inferred from values used by libG^56^ for canonical DNA base pairing. Ligand geometry restraints for Np_*n*_Ns and nucleotide analogs were generated using the Grade web server (version 2.0.14; Global Phasing). The final refinement round in PHENIX included one cycle of ADP refinement only. The refined models were validated using MolProbity^61^ and the wwPDB database^62^ validation server. The final TC-Ap_3_G model was used as the starting model for the TC-GTP, TC-Ap_4_G, TC-Ap_4_A and aTT-Ap_4_A models, which were built and refined analogously to the above description. In the case of TC-GTP, parallelity was also maintained between the GTP base and the adjacent nonpairing template base. The TC-empty structure was built and refined analogously, using the TC-Ap_4_A template-shifted structure as the starting model.

Because of disorder, residues β′/217–339 from the β′ nonconserved domain were not included in any of the models. Additionally, residues σ/346–414, template-strand nucleotides 37–51 and nontemplate-strand nucleotides 3–17 were excluded from the TC-GTP, TC-Ap_4_G, TC-Ap_4_A, aTT-Ap_4_A and TC-empty models.

The CMPcPP molecule in the TC-Ap_3_G, TC-GTP, TC-Ap_4_G and aTT-Ap_4_A models was present in two conformations, one coordinating a magnesium cation (Mg^B^) and one free of magnesium. The magnesium-coordinated conformation of CMPcPP was accompanied by a nearly closed conformation of the trigger loop, whereas the magnesium-free conformation of CMPcPP was associated with an alternative, unstructured conformation of the trigger loop. We describe the Mg^B^-coordinated conformation with a nearly closed trigger loop in the Results. The TC-Ap_4_A model, in which the template is antiscrunched by one nucleotide, comprises a GMPcPP in a single, Mg^B^-coordinated conformation and the trigger loop is unstructured. In the TC-empty structure, the trigger loop is unstructured.

### Reporting summary

Further information on research design is available in the Nature Portfolio Reporting Summary linked to this article.

## Online content

Any methods, additional references, Nature Portfolio reporting summaries, source data, extended data, supplementary information, acknowledgements, peer review information; details of author contributions and competing interests; and statements of data and code availability are available at 10.1038/s41589-025-02134-5.

## Supplementary information

Supplementary Information Supplementary Tables 1–12, Supplementary Figs. 1–10, Supplementary References and Source Data for Supplementary Information.

Reporting Summary

## Source data

Source Data Fig. 1 and Source Data Extended Data Figs. 3–7 Unprocessed gels.

## Figures and Tables

**Extended Data Table 1:**
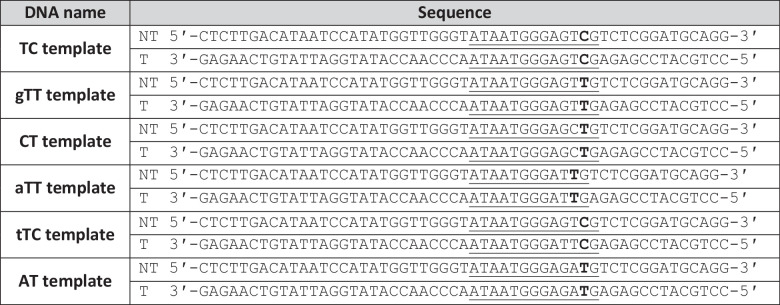
Sequences of template and non-template DNA strands used to construct artificial DNA promoters with pre-formed transcription bubbles

The sequences have various combinations of nucleotides at the −1 and +1 positions (indicated by capital letters in the template names while the −2 position is shown in lower-case). The aTT template is shortened by one nucleotide. NT strand = non-template strand 5′ to 3′; T = template strand 3′ to 5′. The +1 consensus transcription start sites are bold. DNA templates used for transcription and cryo-EM analyses were prepared by annealing NT and T strands with the same name, except for tTC (T strand, Supplementary Table 1), which was annealed with the TC (NT strand). Pre-formed transcription bubbles are underlined.

## Data Availability

LC–MS data are available from Zenodo (10.5281/zenodo.14215049)^63^. Coordinates and maps for *Tt* RNAP de novo transcription initiation complexes were deposited to the Protein Data Bank (PDB) and Electron Microscopy Data Bank (EMDB) under the following accession numbers: TC-Ap_3_G, PDB 9FOG and EMD-50622; TC-Ap_4_G, PDB 9FOK and EMD-50625; TC-Ap_4_A, PDB 9FP3 and EMD-50634; TC-GTP, PDB 9FO6 and EMD-50618; aTT-Ap_4_A, PDB 9FRJ and EMD-50715; TC-empty, PDB 9R75 and EMD-53711. Source data are provided with this paper.
