# Supplementary material for: Molecular insight into 5′ RNA capping with NpnNs by bacterial RNA polymerase
Source: Nat Chem Biol. 2026 Jan 9;22(6):917–24. doi: 10.1038/s41589-025-02134-5 (PMC13226041; doi:10.1038/s41589-025-02134-5)

nature chemical biology

Supplementary information

https://doi.org/10.1038/s41589-025-02134-5

Molecular insight into 5′ RNA capping with Np$_n$Ns by bacterial RNA polymerase

In the format provided by the authors and unedited

Contents:

Supplementary Tables 1-12

Supplementary Figures 1-10

Supplementary References

Source Data Files for SI

Supplementary Tables

Supplementary Table 1: Sequences of all DNA oligonucleotides used for the preparation of all templates, PAGE markers, and the sequence of the TC plasmid. The pre-formed transcription bubbles are underlined. +1 consensus canonical TSS are highlighted in red and bold.

DNA Template	Sequence (5′-3′)	Length
TC (NT strand)	CTCTTGACATAATCCATATGGTTGGGTATAATGGGAGT**C**GTCTCGGATGCAGG	53nt
TC (T strand)	CCTGCATCCGAGAG**C**TGAGGGTAATAACCCAACCATATGGATTATGTCAAGAG	53nt
gTT (NT strand)	CTCTTGACATAATCCATATGGTTGGGTATAATGGGAGT**T**GTCTCGGATGCAGG	53nt
gTT (T strand)	CCTGCATCCGAGAG**T**TGAGGGTAATAACCCAACCATATGGATTATGTCAAGAG	53nt
CT (NT strand)	CTCTTGACATAATCCATATGGTTGGGTATAATGGGAGC**T**GTCTCGGATGCAGG	53nt
CT (T strand)	CCTGCATCCGAGAG**T**CGAGGGTAATAACCCAACCATATGGATTATGTCAAGAG	53nt
aTT (NT strand)	CTCTTGACATAATCCATATGGTTGGGTATAATGGGAT**T**GTCTCGGATGCAGG	52nt
aTT (T strand)	CCTGCATCCGAGAG**T**TAGGGTAATAACCCAACCATATGGATTATGTCAAGAG	52nt
tTC (T strand)	CCTGCATCCGAGAG**C**TTAGGGTAATAACCCAACCATATGGATTATGTCAAGAG	53nt
AT (NT strand)	CTCTTGACATAATCCATATGGTTGGGTATAATGGGAGA**T**GTCTCGGATGCAGG	53nt
AT (T strand)	CCTGCATCCGAGAG**T**AGAGGGTAATAACCCAACCATATGGATTATGTCAAGAG	53nt
RNA Markers	Sequence (5′-3′)	
15-mer (used for template TC)	GCUCUCGGAUGCAGG	15nt
16-mer TC (used for template TC)	AGCUCUCGGAUGCAGG	16nt
15-mer gTT (used for template gTT and CT)	ACUCUCGGAUGCAGG	15nt
16-mer CT (used for template CT)	GACUCUCGGAUGCAGG	16nt
TC plasmid		
5′-CTCTTGACATAATCCATATGGTTGGGTATAATGGGAGA**G**AGCTTGGGTCCCACCTGAC<br>CCCATGCCGAACTCAGAAGTGAAACGCCGTAGCGCCGATGGTAGTGTGGGGTCTCCCCATGCGAGAGTA<br>GGGAACTGCCAGGCATCAAATAAAACGAAAGGCTCAGTCGAAAGACTGGGCCTTTCGTTTTATCTGTTG<br>TTTGTCGGTGAACGCTCTCCTGAGTAGGACAAATCCGCCGGGAGCGGATTTGAACGTTGCGAAGCAACG<br>GCCCGGAGGGTGGCGGGCAGGACGCCCGCCATAAACTGCCAGGCATCAAATTAAGCAGAAGGCCATCCT<br>GACGGATGGCCTTTTTGCGTTTCTACAAACTCTTCCTGTCGTCATATCTACAAGCCATCCCCCCACAGA<br>TACGGTAAACTAGCCTCGTTTTTGCATCAGGAAAGCAGCTATGAACCACTCCTTAAAACCCTGGAACAC<br>ATTTGGCATTGATCATAATGCTCAGCACATTGTATGTGCCGAAGACGAACAACAATTACTCAATGCCTG<br>GCAGTATGCAACCGCAGAAGGACAACCCGTTCTTATTCTGGGTGAAGGAAGTAATGTACTTTTTCTGGA<br>GGACTATCGCGGCACGGTGATCATCAACCGGATCAAAGGTATCGAAATTCATGATGAACCTGATGCGTG<br>GTATTTACATGTAGGAGCCGGAGAAAACTGGCATCGTCTGGTAAAATACACTTTGCAGGAAGGTATGCC<br>TGGTCTGGAAAATCTGGCATTAATTCCTGGTTGTGTCGGCTCATCACCTATCCAGAATATTGGTGCTTA<br>TGGCGTAGAATTACAGCGAGTTTGCGCTTATGTTGATTCTGTTGAACTGGCGACAGGCAAGCAAGTGCG<br>CTTAAGGGGTCTGACGCTCAGTGGAACGAAAACTCACGTTAAGGATTTTGGTCATGAGATTATCAAAA		

AGGATCTTCACCTAGATCCTTTTAAATTAAAAATGAAGTTTTAAATCAATCTAAAGTATATATGAGTAA
ACTTGGTCTGACAGTTACCAATGCTTAATCAGTGAGGCACCTATCTCAGCGATCTGTCTATTTCGTTCA
TCCATAGTTGCCTGACTCCCCGTCGTGTAGATAACTACGATACGGGAGGGCTTACCATCTGGCCCCAGT
GCTGCAATGATACCGCGAGACCCACGCTCACCGGCTCCAGATTTATCAGCAATAAACCAGCCAGCCGGA
AGGGCCGAGCGCAGAAGTGGTCCAAGCTAGCTTAACTAACTAACAGCTTACTCCCCATCCCCCCGAAAG
ATTTTTTTAACTATAAACGCTGATGGAAGCGTTTATGCGGAAGAGGTAAAGCCCTTCCCGAGTAACAAA
AAAACAACAGCATAAATAACCCCGCTCTTACACATTCCAGCCCTGAAAAAGGGCATCAAATTAAACCAC
ACCTATGGTGTATGCATTTATTTGCATACATTCAATCAATTGTTATCTAAGGAAATACTTACATATGGT
TCGTGCAAACAAACGCAACGAGGCTCTACGAATCGAGAGTGCGTTGCTTAACAAAATCGCAATGCTTGG
AACTGAGAAGACAGCGGAAGCTGTGGGCGTTGATAAGTCGCAGATCAGCAGGTGGAAGAGGGACTGGAT
TCCAAAGTTCTCAATGCTGCTTGCTGTTCTTGAATGGGGGGTCGTTGGGCAATAAGGGCTGCACGCGCA
CTTTTATCCGCCTCTGCTGCGCTCCGCCACCGTACGTAAATTTATGGTTGGTTATGAAATGCTGGCAGA
GACCCAGCGAGACCTGACCGCAGAACAGGCAGCAGAGCGTTTGCGCGCAGTCAGCGATATCCATTTTCG
CGAATCCGGAGTGTAAGAAATGAGTCTGAAAGAAAAAACACAATCTCTGTTTGCCAACGCATTTGGCTA
CCCTGCCACTCACACCATTCAGGCGCCTGGCCGCGTGAATTTGATTGGTGAACACACCGACTACAACGA
CGGTTTCGTTCTGCCCTGCGCGATTGATTATCAAACCGTGATCAGTTGTGCACCACGCGATGACCGTAA
AGTTCGCGTGATGGCAGCCGATTATGAAAATCAGCTCGACGAGTTTTCCCTCGATGCGCCCATTGTCGC
ACATGAAAACTATCAATGGGCTAACTACGTTCGTGGCGTGGTGAAACATCTGCAACTGCGTAACAACAG
CTTCGGCGGCGTGGACATGGTGATCAGCGGCAATGTGCCGCAGGGTGCCGGGTTAAGTTCTTCCGCTTC
ACTGGAAGTCGCGGTCGGAACCGTATTGCAGCAGCTTTATCATCTGCCGCTGGACGGCGCACAAATCGC
GCTTAACGGTCAGGAAGCAGAAAACCAGTTTGTAGGCTGTAACTGCGGGATCATGGATCAGCTAATTTC
CGCGCTCGGCAAGAAAGATCATGCCTTGCTGATCGATTGCCGCTCACTGGGGACCAAAGCAGTTTCCAT
GCCCAAAGGTGTGGCTGTCGTCATCATCAACAGTAACTTCAAACGTACCCTGGTTGGCAGCGAATACAA
CACCCGTCGTGAACAGTGCGAAACCGGTGCGCGTTTCTTCCAGCAGCCAGCCCTGCGTGATGTCACCAT
TGAAGAGTTCAACGCTGTTGCGCATGAACTGGACCCGATCGTGGCAAAACGCGTGCGTCATATACTGAC
TGAAAACGCCCGCACCGTTGAAGCTGCCAGCGCGCTGGAGCAAGGCGACCTGAAACGTATGGGCGAGTT
GATGGCGGAGTCTCATGCCTCTATGCGCGATGATTTCGAAATCACCGTGCCGCAAATTGACACTCTGGT
AGAAATCGTCAAAGCTGTGATTGGCGACAAAGGTGGCGTACGCATGACCGGCGGCGGATTTGGCGGCTG
TATCGTCGCGCTGATCCCGGAAGAGCTGGTGCCTGCCGTACAGCAAGCTGTCGCTGAACAATATGAAGC
AAAAACAGGTATTAAAGAGACTTTTTACGTTTGTAAACCATCACAAGGAGCAGGACAGTGCTGAACGAA
ACTCCCGCACTGGCACCCGATGGTCAGCCGTACCGACTGTTCTGCCTCGCGCGTTTCGGTGATGACGGT
GAAAACCTCTGACACATGCAGCTCCCGGAGACGGTCACAGCTTGTCTGTAAGCGGATGCCGGGAGCAGA
CAAGCCCGTCAGGGCGCGTCAGCGGGTGTTGGCGGGTGTCGGGGCGCAGCCATGACCCAGTCACGTAGC
GATAGCGGAGTGTATACTGGCTTAACTATGCGGCATCAGAGCAGATTGTACTGAGAGTGCACCATATGC
GGTGTGAAATACCGCACAGATGCGTAAGGAGAAAATACCGCATCAGGCGCTCTTCCGCTTCCTCGCTCA
CTGACTCGCTGCGCTCGGTCGTTCGGCTGCGGCGAGCGGTATCAGCTCACTCAAAGGCGGTAATACGGT
TATCCACAGAATCAGGGGATAACGCAGGAAAGAACATGTGAGCAAAAGGCCAGCAAAAGGCCAGGAACC
GTAAAAAGGCCGCGTTGCTGGCGTTTTTCCATAGGCTCCGCCCCCCTGACGAGCATCACAAAAATCGAC
GCTCAAGTCAGAGGTGGCGAAACCCGACAGGACTATAAAGATACCAGGCGTTTCCCCCTGGAAGCTCCC
TCGTGCGCTCTCCTGTTCCGACCCTGCCGCTTACCGGATACCTGTCCGCCTTTCTCCCTTCGGGAAGCG
TGGCGCTTTCTCATAGCTCACGCTGTAGGTATCTCAGTTCGGTGTAGGTCGTTCGCTCCAAGCTGGGCT
GTGTGCACGAACCCCCCGTTCAGCCCGACCGCTGCGCCTTATCCGGTAACTATCGTCTTGAGTCCAACC
CGGTAAGACACGACTTATCGCCACTGGCAGCAGCCACTGGTAACAGGATTAGCAGAGCGAGGTATGTAG
GCGGTGCTACAGAGTTCTTGAAGTGGTGGCCTAACTACGGCTACACTAGAAGGACAGTATTTGGTATCT
GCGCTCTGCTGAAGCCAGTTACCTTCGGAAAAAGAGTTGGTAGCTCTTGATCCGGCAAACAAACCACCG
CTGGTAGCGGTGGTTTTTTTGTTTGCAAGCAGCAGATTACGCGCAGAAAAAAAGGATCTCAAGAAGATC
CTTTGATCTTTTCTACGGGGTCTGACGCTCAGTGGAACGAAAACTCACGTTAAGGGATTTTGGTCATGA
GATTATCAAAAAGGATCTTCACCTAGATCCTTTTAAATTAAAAATGAAGTTTTAAATCAATCTAAAGTA

```
TATATGAGTAAACTTGGTCTGACAGTTACCAATGCTTAATCAGTGAGGCACCTATCTCAGCGATCTGTC
TATTTCGTTCATCCATAGTTGCCTGACTCCCCGTCGTGTAGATAACTACGATACGGGAGGGCTTACCAT
CTGGCCCCAGTGCTGCAATGATACCGCGAGACCCACGCTCACCGGCTCCAGATTTATCAGCAATAAACC
AGCCAGCCGGAAGGGCCGAGCGCAGAAGTGGTCCTGCAACTTTATCCGCCTCCATCCAGTCTATTAATT
GTTGCCGGGAAGCTAGAGTAAGTAGTTCGCCAGTTAATAGTTTGCGCAACGTTGTTGCCATTGCTGCAG
GCATCGTGGTGTCACGCTCGTCGTTTGGTATGGCTTCATTCAGCTCCGGTTCCCAACGATCAAGGCGAG
TTACATGATCCCCCATGTTGTGCAAAAAAGCGGTTAGCTCCTTCGGTCCTCCGATCGTTGTCAGAAGTA
AGTTGGCCGCAGTGTTATCACTCATGGTTATGGCAGCACTGCATAATTCTCTTACTGTCATGCCATCCG
TAAGATGCTTTTCTGTGACTGGTGAGTACTCAACCAAGTCATTCTGAGAATAGTGTATGCGGCGACCGA
GTTGCTCTTGCCCGGCGTCAACACGGGATAATACCGCGCCACATAGCAGAACTTTAAAAGTGCTCATCA
TTGGAAAACGTTCTTCGGGGCGAAAACTCTCAAGGATCTTACCGCTGTTGAGATCCAGTTCGATGTAAC
CCACTCGTGCACCCAACTGATCTTCAGCATCTTTTACTTTCACCAGCGTTTCTGGGTGAGCAAAAACAG
GAAGGCAAAATGCCGCAAAAAAGGGAATAAGGGCGACACGGAAATGTTGAATACTCATACTCTTCCTTT
TTCAATATTATTGAAGCATTTATCAGGGTTATTGTCTCATGAGCGGATACATATTTGAATGTATTTAGA
AAAATAAACAAATAGGGGTTCCGCGCACATTTCCCCGAAAAGTGCCACCTGACGTCTAAGAAACCATTA
TTATCATGACATTAACCTATAAAAATAGGCGTATCACGAGGCCCTTTCGTCTTCAAGAATT-3′
```

Supplementary Table 2: Final concentrations of non-canonical initiating nucleotides (NCINs) and canonical initiating nucleotides (iNTPs) in the IVT reaction mixtures.

In each control (CTRL AA, AG and GG) the concentration of ATP and GTP was adjusted in a way that each control contained the same total final concentration of initiating nucleotides.

NCINs or iNTPs	CTRL AA	Ap$_n$A	CTRL AG	Ap$_n$G	CTRL GG	Gp$_n$G
ATP (mM)	2.2	0.6	1.4	0.6	0.6	0.6
GTP (mM)	0.6	0.6	1.4	0.6	2.2	0.6
Np$_n$Ns (mM)	0	1.6	0	1.6	0	1.6
Final concentration (mM)	2.8	2.8	2.8	2.8	2.8	2.8

Supplementary Table 3: RNA products detected by LC-MS with template TC and addition of Np$_n$Ns.

Sample	Name	Sequence	Calculated Mass (m/z)	Experimental Mass (m/z)	Retention time (min)
CTRL AA	(+1)TSS-RNA	GCUCUCGGAUGCAGG	1260.6370	1260.6249	6.70
	(+1)TSS-RNA+3′C	GCUCUCGGAUGCAGGC	1336.8970	1336.8844	6.66
	(-1)TSS-RNA	AGCUCUCGGAUGCAGG	1342.9000	1342.8878	6.65
	(-1)TSS-RNA+3′C	AGCUCUCGGAUGCAGGC	1419.1600	1419.1477	6.65
Ap$_3$A	cap(+1)TSS-RNA	N/A			
	cap(+1)TSS-RNA+3′C	N/A			
	cap(-1)TSS-RNA	Ap$_3$AGCUCUCGGAUGCAGG	1405.1716	1405.1566	6.65
	cap(-1)TSS-RNA+3′C	Ap$_3$AGCUCUCGGAUGCAGGC	1481.4320	N/D	N/D
Ap$_4$A	cap(+1)TSS-RNA	N/A			
	cap(+1)TSS-RNA+3′C	N/A			
	cap(-1)TSS-RNA	Ap$_4$AGCUCUCGGAUGCAGG	1425.1632	1425.1516	6.69
	cap(-1)TSS-RNA+3′C	Ap$_4$AGCUCUCGGAUGCAGGC	1501.4230	1501.4064	6.69
Ap$_3$G	cap(+1)TSS-RNA	Ap$_3$GCUCUCGGAUGCAGG	1322.9084	1322.9014	6.63

	cap(+1)TSS-RNA+3'C	Ap$_3$GCUCUCGGAUGCAGGC	1399.1680	1399.1587	6.59
	cap(-1)TSS-RNA	Gp$_3$AGCUCUCGGAUGCAGG	1409.1700	1409.1588	6.82
	cap(-1)TSS-RNA+3'C	Gp$_3$AGCUCUCGGAUGCAGGC	1485.4300	N/D	N/D
**Ap$_4$G**	cap(+1)TSS-RNA	Ap$_4$GCUCUCGGAUGCAGG	1342.9000	1342.8922	6.69
	cap(+1)TSS-RNA+3'C	Ap$_4$GCUCUCGGAUGCAGGC	1419.1600	1419.1494	6.68
	cap(-1)TSS-RNA	Gp$_4$AGCUCUCGGAUGCAGG	1429.1620	1429.1505	6.88
	cap(-1)TSS-RNA+3'C	Gp$_4$AGCUCUCGGAUGCAGGC	1505.4222	N/D	N/D
**Gp$_3$G**	cap(+1)TSS-RNA	Gp$_3$GCUCUCGGAUGCAGG	1326.9072	1326.8931	6.78
	cap(+1)TSS-RNA+3'C	Gp$_3$GCUCUCGGAUGCAGGC	1403.1676	1403.1488	6.76
	cap(-1)TSS-RNA	N/A			
	cap(-1)TSS-RNA+3'C	N/A			
**Gp$_4$G**	cap(+1)TSS-RNA	Gp$_4$GCUCUCGGAUGCAGG	1346.8982	1346.8846	6.88
	cap(+1)TSS-RNA+3'C	Gp$_4$GCUCUCGGAUGCAGGC	1423.1592	1423.1426	6.87
	cap(-1)TSS-RNA	N/A			
	cap(-1)TSS-RNA+3'C	N/A			

N/A: not applicable, N/D: not detected, the first initiating nucleotide is marked red and an extra C added at the 3′ end is marked blue

Supplementary Table 4: RNA products detected by LC-MS in IVT experiments with the TC plasmid and Np$_n$Ns.

5′ fragments of RNA products after RNase A digestion are shown (only first part of sequence with cap is shown).

Sample	Name	Sequence	Calculated Mass (m/z)	Experimental Mass (m/z)	Retention time (min)
**CTRL**	(+1)TSS-RNA	GAGCp	790.0148	790.0406	2.42
	(-1)TSS-RNA	AGAGCp*	954.5681	954.5657	2.60
**Ap$_3$A**	cap(+1)TSS-RNA	N/A			
	cap(-1)TSS-RNA	Ap$_3$AGAGCp	1079.116	1079.1082	2.57
**Ap$_4$A**	cap(+1)TSS-RNA	N/A			
	cap(-1)TSS-RNA	Ap$_4$AGAGCp	1119.0944	1119.0944	2.71
**Ap$_3$G**	cap(+1)TSS-RNA	Ap$_3$GAGCp	914.5839	914.5836	2.39
	cap(-1)TSS-RNA	Gp$_3$AGAGCp	1087.1087	1087.1095	2.50
**Ap$_4$G**	cap(+1)TSS-RNA	Ap$_4$GAGCp*	954.5681	954.5657	2.60
	cap(-1)TSS-RNA	Gp$_4$AGAGCp	1127.0918	1127.0901	2.71
**Gp$_3$G**	cap(+1)TSS-RNA	Gp$_3$GAGCp	922.5828	922.5804	2.34
	cap(-1)TSS-RNA	N/A			
**Gp$_4$G**	cap(+1)TSS-RNA	Gp$_4$GAGCp	962.5656	962.5643	2.54
	cap(-1)TSS-RNA	N/A			

N/A: not applicable, N/D: not detected, same molecular formula*. The first initiating nucleotide is marked red.

Supplementary Table 5: RNA products detected by LC-MS with template gTT and addition of Np$_n$Ns.

Sample	Name	Sequence	Calculated Mass (m/z)	Experimental Mass (m/z)	Retention time (min)
CTRL AA	(+1)TSS-RNA	ACUCUCGGAUGCAGG	1256.6382	1256.6382	6.70
	(+1)TSS-RNA+3'C	ACUCUCGGAUGCAGGC	1332.8986	1332.8862	6.81
	(-1)TSS-RNA	AACUCUCGGAUGCAGG	1338.9015	1338.8881	6.73
	(-1)TSS-RNA+3'C	AACUCUCGGAUGCAGGC	1415.1617	1415.1471	6.85
Ap_3A	cap(+1)TSS-RNA	Ap_3ACUCUCGGAUGCAGG	1318.9098	1318.8971	6.81
	cap(+1)TSS-RNA+3'C	Ap_3ACUCUCGGAUGCAGGC	1395.1700	1395.1576	6.95
	cap(-1)TSS-RNA	Ap_3AACUCUCGGAUGCAGG	1401.1729	N/D	N/D
	cap(-1)TSS-RNA+3'C	Ap_3AACUCUCGGAUGCAGGC	1477.4332	N/D	N/D
Ap_4A	cap(+1)TSS-RNA	Ap_4ACUCUCGGAUGCAGG	1338.9014	1338.8876	6.88
	cap(+1)TSS-RNA+3'C	Ap_4ACUCUCGGAUGCAGGC	1415.1617	1415.1467	7.00
	cap(-1)TSS-RNA	Ap_4AACUCUCGGAUGCAGG	1421.1644	1421.1490	6.90
	cap(-1)TSS-RNA+3'C	Ap_4AACUCUCGGAUGCAGGC	1497.4248	1497.4067	7.04
Ap_3G	cap(+1)TSS-RNA	Gp_3ACUCUCGGAUGCAGG	1322.9084	1322.8979	7.01
	cap(+1)TSS-RNA+3'C	Gp_3ACUCUCGGAUGCAGGC	1399.1688	1399.1573	7.10
	cap(-1)TSS-RNA	Gp_3AACUCUCGGAUGCAGG	1405.1716	1405.1605	7.04
	cap(-1)TSS-RNA+3'C	Gp_3AACUCUCGGAUGCAGGC	1481.4320	1481.4180	7.16
Ap_4G	cap(+1)TSS-RNA	Gp_4ACUCUCGGAUGCAGG	1342.9000	1342.8900	7.09
	cap(+1)TSS-RNA+3'C	Gp_4ACUCUCGGAUGCAGGC	1419.1604	1419.1500	7.19
	cap(-1)TSS-RNA	Gp_4AACUCUCGGAUGCAGG	1425.1630	1425.1519	7.13
	cap(-1)TSS-RNA+3'C	Gp_4AACUCUCGGAUGCAGGC	1501.4236	1501.4131	7.23
Gp_3G	cap(+1)TSS-RNA	N/A			
	cap(+1)TSS-RNA+3'C	N/A			
	cap(-1)TSS-RNA	N/A			
	cap(-1)TSS-RNA+3'C	N/A			
Gp_4G	cap(+1)TSS-RNA	N/A			
	cap(+1)TSS-RNA+3'C	N/A			
	cap(-1)TSS-RNA	N/A			
	cap(-1)TSS-RNA+3'C	N/A			

N/A: not applicable, N/D: not detected, the first initiating nucleotide is marked red and an extra C added at the 3' end is marked blue.

Supplementary Table 6: RNA products detected by LC-MS with template CT and addition of Np$_n$Ns.

Sample	Name	Sequence	Calculated Mass (m/z)	Experimental Mass (m/z)	Retention time (min)
CTRL AA	(+1)TSS-RNA	ACUCUCGGAUGCAGG	1256.6382	1256.6339	6.73
	(+1)TSS-RNA+3'C	ACUCUCGGAUGCAGGC	1332.8986	1332.8944	6.82
	(-1)TSS-RNA	GACUCUCGGAUGCAGG	1342.9000	1342.8949	6.88
	(-1)TSS-RNA+3'C	GACUCUCGGAUGCAGGC	1419.1604	1419.1543	6.97
Ap_3A	cap(+1)TSS-RNA	Ap_3ACUCUCGGAUGCAGG	1318.9098	1318.8961	6.43
	cap(+1)TSS-RNA+3'C	Ap_3ACUCUCGGAUGCAGGC	1395.1700	1395.1571	6.56
	cap(-1)TSS-RNA	N/A			
	cap(-1)TSS-RNA+3'C	N/A			
Ap_4A	cap(+1)TSS-RNA	Ap_4ACUCUCGGAUGCAGG	1338.9014	1338.8892	6.51
	cap(+1)TSS-RNA+3'C	Ap_4ACUCUCGGAUGCAGGC	1415.1617	1415.1473	6.63
	cap(-1)TSS-RNA	N/A			
	cap(-1)TSS-RNA+3'C	N/A			
Ap_3G	cap(+1)TSS-RNA	Gp_3ACUCUCGGAUGCAGG	1322.9084	1322.8954	6.61
	cap(+1)TSS-RNA+3'C	Gp_3ACUCUCGGAUGCAGGC	1415.1617	N/D	N/D
	cap(-1)TSS-RNA	Ap_3GACUCUCGGAUGCAGG	1405.1716	1405.1581	6.58
	cap(-1)TSS-RNA+3'C	Ap_3GACUCUCGGAUGCAGGC	1481.432	N/D	N/D
Ap_4G	cap(+1)TSS-RNA	Gp_4ACUCUCGGAUGCAGG	1342.9000	1342.8890	6.70
	cap(+1)TSS-RNA+3'C	Gp_4ACUCUCGGAUGCAGGC	1419.1604	1419.1470	6.77
	cap(-1)TSS-RNA	Ap_4GACUCUCGGAUGCAGG	1425.1632	1425.1500	6.68
	cap(-1)TSS-RNA+3'C	Ap_4GACUCUCGGAUGCAGGC	1501.4236	1501.4103	6.75
Gp_3G	cap(+1)TSS-RNA	N/A			
	cap(+1)TSS-RNA+3'C	N/A			
	cap(-1)TSS-RNA	Gp_3GACUCUCGGAUGCAGG	1409.1704	1409.1586	6.82
	cap(-1)TSS-RNA+3'C	Gp_3GACUCUCGGAUGCAGGC	1485.4307	1485.4169	6.90
Gp_4G	cap(+1)TSS-RNA	N/A			
	cap(+1)TSS-RNA+3'C	N/A			
	cap(-1)TSS-RNA	Gp_4GACUCUCGGAUGCAGG	1429.1620	1429.1503	6.87
	cap(-1)TSS-RNA+3'C	Gp_4GACUCUCGGAUGCAGGC	1505.4222	1505.4067	6.94

N/A: not applicable, N/D: not detected, the first initiating nucleotide is marked red and an extra C added at the 3' end is marked blue.

Supplementary Table 7: Cryo-EM structure determination and validation statistics.

**Name of structure**	**TC-Ap$_3$G**	**TC-Ap$_4$G**	**TC-Ap$_4$A**	**TC-GTP**	**aTT-Ap$_4$A**	**TC-empty**
**PDB ID**	**9FOG**	**9FOK**	**9FP3**	**9FO6**	**9FRJ**	**9R75**
**EMDB ID**	**EMD-50622**	**EMD-50625**	**EMD-50634**	**EMD-50618**	**EMD-50715**	**EMD-53711**
**Data collection and processing**						
Microscope	Titan Krios	Titan Krios	Titan Krios	Titan Krios	Titan Krios	Titan Krios
Voltage (kV)	300	300	300	300	300	300
Camera	Gatan K3 BioQuantum	Gatan K3 BioQuantum	Gatan K3 BioQuantum	Gatan K3 BioQuantum	Gatan K3 BioQuantum	Gatan K3 BioQuantum
Magnification (x)	105,000	105,000	105,000	105,000	105,000	105,000
Nominal defocus range (negative µm)	0.5-3.0	0.7-2.1	0.8-2.4	0.9-2.4	1.2-2.6	0.9-2.4
Exposure time (s)	2.5	2.1	3	2.7	2.5	2.7
Electron exposure ($e^-/Å^2$)	50	40	50	50	50	50
Number of frames collected (no.)	40	40	46	40	40	40
Number of frames processed (no.)	25	25	25	25	25	25
Pixel size (Å)	0.8336	0.8336	0.8336	0.8336	0.8336	0.8336
Micrographs (no.)	19,372	64,451	45,250	29,943	45,785	29,943
Total particle images (no.)	6,104,447	25,794,738	7,291,435	13,198,319	16,418,707	13,198,319
**Refinement**						
Particles per class (no.)	181,619	96,736	232,335	164,165	76,802	82,750
Map resolution (Å), 0.143 FSC	2.51	2.74	2.42	2.76	2.66	3.80
Map sharpening B factor ($Å^2$)	-34.78	-55.01	-35.35	-52.36	-35.81	-133.832
Map versus model cross-correlation	0.89	0.87	0.88	0.90	0.85	0.87
Map versus model cross-correlation for ligands	0.58	0.75	0.46	0.71	0.69	0.80
**Model composition**						
Non-hydrogen atoms	28,716	27,370	27,294	27,489	27,579	27,174
Protein residues	3,348	3,268	3,262	3,270	3,276	3,262
Nucleotide residues	97	66	66	67	72	66
Chains	9	9	9	9	10	9
Ligands	9	9	8	9	9	4
**B factors ($Å^2$)** *min/max/mean*						
Protein	9.78/217.68/56.44	5.30/164.06/64.87	8.22/148.71/54.60	15.17/167.84/64.20	6.97/154.07/64.09	16.40/214.62/74.89
Nucleotide	47.21/273.95/175.56	31.74/220.04/133.88	40.29/247.19/141.07	63.40/276.29/161.17	19.12/254.52/142.24	65.84/316.37/181.71
Ligand	43.95/113.76/77.14	31.47/131.70/49.47	53.81/330.11/155.68	47.76/112.02/66.98	24.74/113.45/41.63	28.51/165.53/87.79
**R.m.s. deviations**						
Bond lengths (Å)	0.004	0.003	0.003	0.005	0.003	0.005
Bond angles (°)	0.594	0.598	0.566	0.600	0.687	1.054
**Validation**						
MolProbity score	1.35	1.40	1.38	1.31	1.47	1.48
All-atom clashscore	6.12	7.25	6.12	5.48	8.05	8.93
Rotamer outliers (%)	0.42	0.54	0.29	0.61	0.64	0
**Ramachandran plot**						
Favored (%)	97.95	98.24	97.81	97.93	97.87	98.02
Allowed (%)	2.05	1.76	2.19	2.07	2.13	1.98
Outliers (%)	0	0	0	0	0	0

Supplementary Table 8: Particles selection chart for the cryo-EM single particle analysis data processing workflow

AS: active site

	TC-Ap_3G	TC-Ap_4G	TC-Ap_4A	TC-GTP	aTT-Ap_4A
Micrographs (no.)	19,372	64,451	45,250	29,943	45,785
Total particle images (no.)	6,104,447	25,794,738	7,291,435	13,198,319	16,418,707
2D Classified particles (no.)	2,971,744	12,295,872	4,928,184	6,155,512	6,129,919
**Global 3D Classification particles I (no.):**					
DNA holoenzyme	999,263	1,957,157	1,044,879	632,827	2,512,248
Apoenzyme	1,310,827	4,289,595	3,412,238	3,221,027	2,734,320
Low-resolution	661,654	6,049,120	471,067	2,301,658	883,351
**3D Classification particles II (no.):**					
Occupied AS	280,496	812,210	310,088	370,381	900,837
Unoccupied AS	510,024	252,041	488,002	190,766	266,541
Apoenzyme	125,324	513,542	0	0	1,207,157
Low-resolution	83,419	379,364	246,789	71,680	137,713
**Broad core focused 3D Classification (no.):**					
Occupied AS	181,619	308,220	232,335	164,165	154,119
Unoccupied AS	98,877	503,990	77,739	206,216	746,332
**AS focused 3D Classification (no.):**					
Occupied AS	-	96,736	-	-	76,802
Low-resolution AS	-	12,442	-	-	29,029
Unoccupied AS	-	199,042	-	-	48,288

Supplementary Table 9: HPLC gradient of separation for LC-MS analysis.

HILIC mode. Mobile phase A: 20 mM ammonium acetate in 90:10 v/v mixture of acetonitrile and ultrapure water, mobile phase B: 20 mM ammonium acetate in ultrapure water.

Time (min)	Flow (mL/min)	Mobile phase A (%)	Mobile phase B (%)	Curve
0	1	70	30	Initial
12	1	70	30	4
14	1	40	60	6
14.1	1	40	60	6
19.9	1	70	30	6
20	0.1	70	30	6

Curve 4 = concave shape of gradient curve; Curve 6 = linear shape of gradient curve (For mobile phase B)

Supplementary Table 10: Ionization parameters for LC-MS analysis.

**Parameters (HILIC mode)**	
Ion Mode	Negative
Mass Range (m/z)	300 - 3000
Capillary Voltage (V)	2200
Sampling Cone (V)	40
Source Offset (V)	40
Source Temperature (°C)	150
Desolvation Temperature (°C)	500
Cone Gas (L/h)	50
Desolvation Gas (L/h)	900
Collision Energy (eV)	6

Supplementary Table 11*:* HPLC gradient of separation for LC-MS analysis.

Ion-pairing mode. Mobile phase A: 15 mM triethylamine and 400 mM HFIP in ultrapure water, mobile phase B: 15 mM triethylamine and 400 mM HFIP in methanol.

**Time (min)**	**Flow (mL/min)**	**Mobile phase A (%)**	**Mobile phase B (%)**	**Curve**
0	0.5	98	2	Initial
1	0.5	98	2	4
4	0.5	75	25	4
4.5	0.5	10	90	6
6	0.5	10	90	6
6.1	0.5	98	2	6
9.9	0.5	98	2	6
10	0.05	98	2	6

Curve 4 = concave shape of gradient curve; Curve 6 = linear shape of gradient curve (For mobile phase B)

Supplementary Table 12: Ionization parameters for LC-MS analysis.

Parameters (Ion-pairing mode)	
Ion Mode	Negative
Mass Range (m/z)	200 - 1200
Capillary Voltage (V)	2000
Sampling Cone (V)	55
Source Offset (V)	40
Source Temperature (°C)	140
Desolvation Temperature (°C)	500
Cone Gas (L/h)	50
Desolvation Gas (L/h)	900
Collision Energy (eV)	6

Supplementary Figures

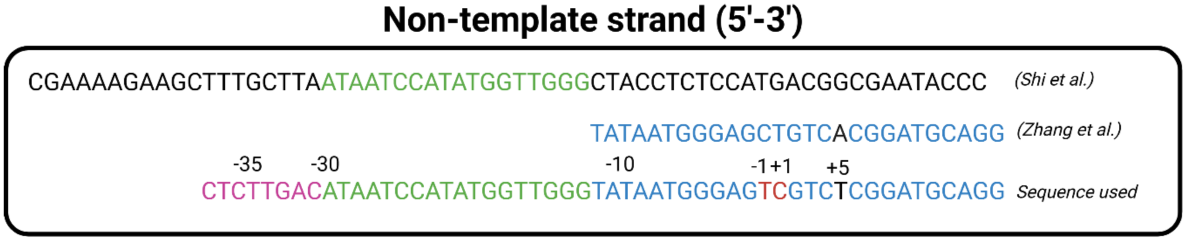

Supplementary Figure 1: DNA promoter design.

The DNA promoter was designed using different sources: the sequence from positions -12 to +15, highlighted in blue, was taken from Zhang *et al.*[1] where the adenine at position +5 was substituted with thymine (black). The sequence from positions -30 to -13, highlighted in green, was used in the study by Shi *et al.*[2]. The sequence from positions -38 to -31, highlighted in magenta, was predicted based on the known consensus sequence of *Thermus thermophilus* promoters. The positions -1 and +1 were substituted to thymine and cytidine, respectively, labeled in red. The final obtained sequence was used to create the reference template named TC. All other variants used in this study (gTT, CT, and AT) were derived from this TC sequence by introducing specific mutations at the +1 and -1 positions. Template aTT was created from the template gTT by a deletion in position -2. Created in BioRender. Serianni, V. (2025) https://BioRender.com/gcnts5s

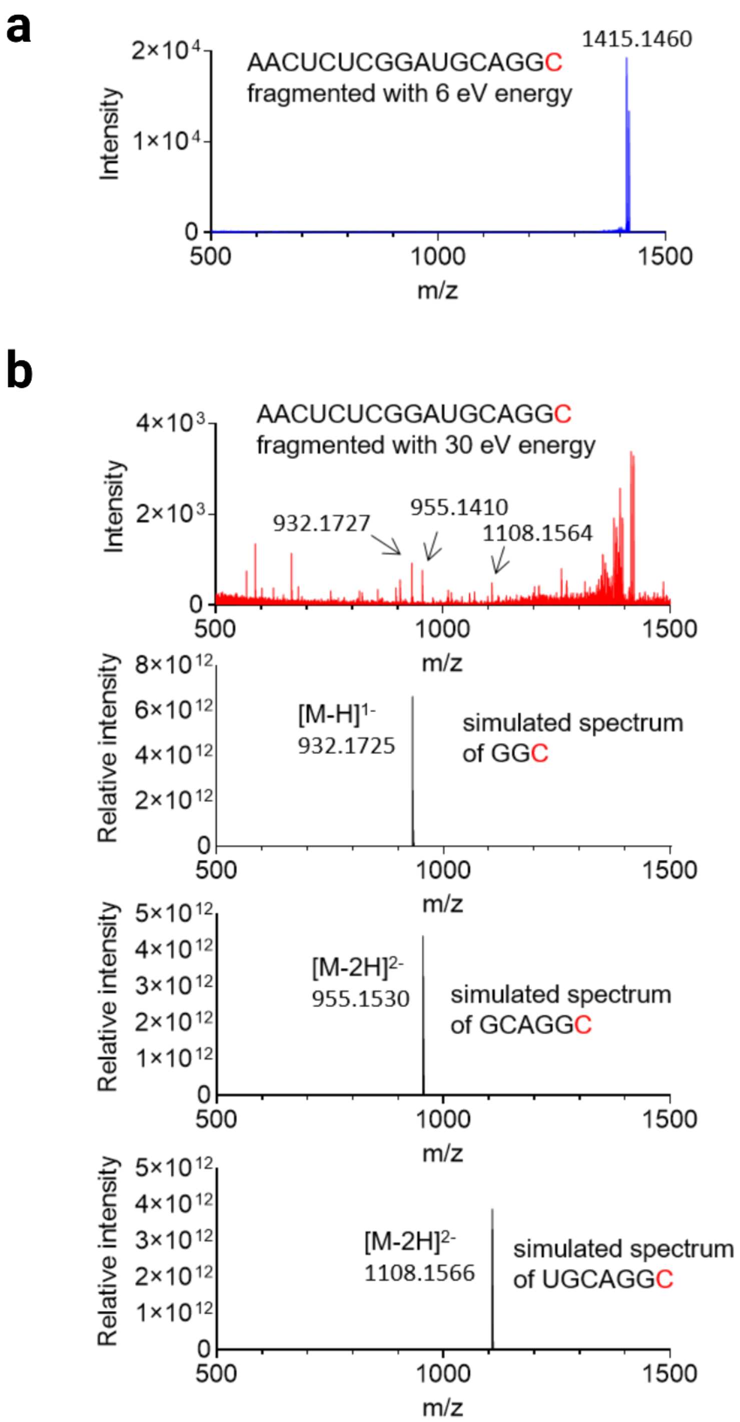

Supplementary Figure 2: LC-MS analysis of a control IVT product.

a, Mass spectrum of a control IVT product with the template gTT with m/z 1415.146 acquired with low fragmentation energy. **b**, Mass spectrum of a control IVT product with the gTT template with m/z 1415.146 acquired with high fragmentation energy compared to simulated spectra of predicted fragments indicating the presence of a C at the 3′ end. Created in BioRender. Serianni, V. (2025) https://BioRender.com/6g4e20s.

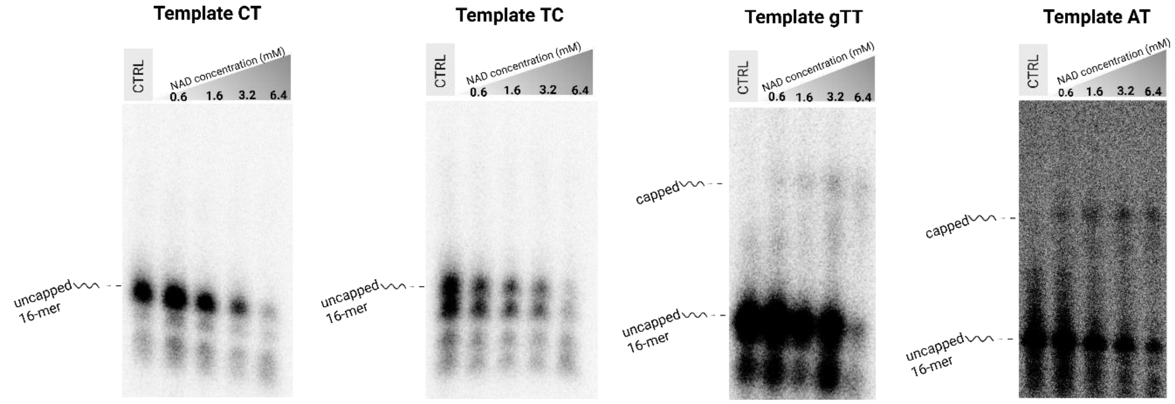

Supplementary Figure 3: PAGE analysis (with APB) of RNA products from IVT experiments using NAD and four different templates (CT, TC, gTT, AT).

We tested three different templates (CT, TC and gTT) at various concentrations of NAD (from 0.6 mM to 6.4 mM). In the cases of the templates CT and TC, we did not obtain any NAD-RNA. We observed only traces of NAD-RNA using the template gTT. Because the reported X-ray structure of NAD capping was obtained with a template having an A at position −1 and a T at position +1[3], we designed a template AT accordingly (Supplementary Table 1). However, the amount of NAD-capped RNA was similar as in the experiment with the template gTT. These experiments confirmed that NAD is indeed a much less potent NCIN than NTPs and Np$_n$Ns. Each CTRL experiment is an IVT with regular NTPs and without added NAD and serves as a molecular weight marker for 16 nt (see Fig. 1e, Extended Data Fig. 4e, 6d). The IVTs were performed in duplicates. Created in BioRender. Serianni, V. (2025) https://BioRender.com/1yugbxd.

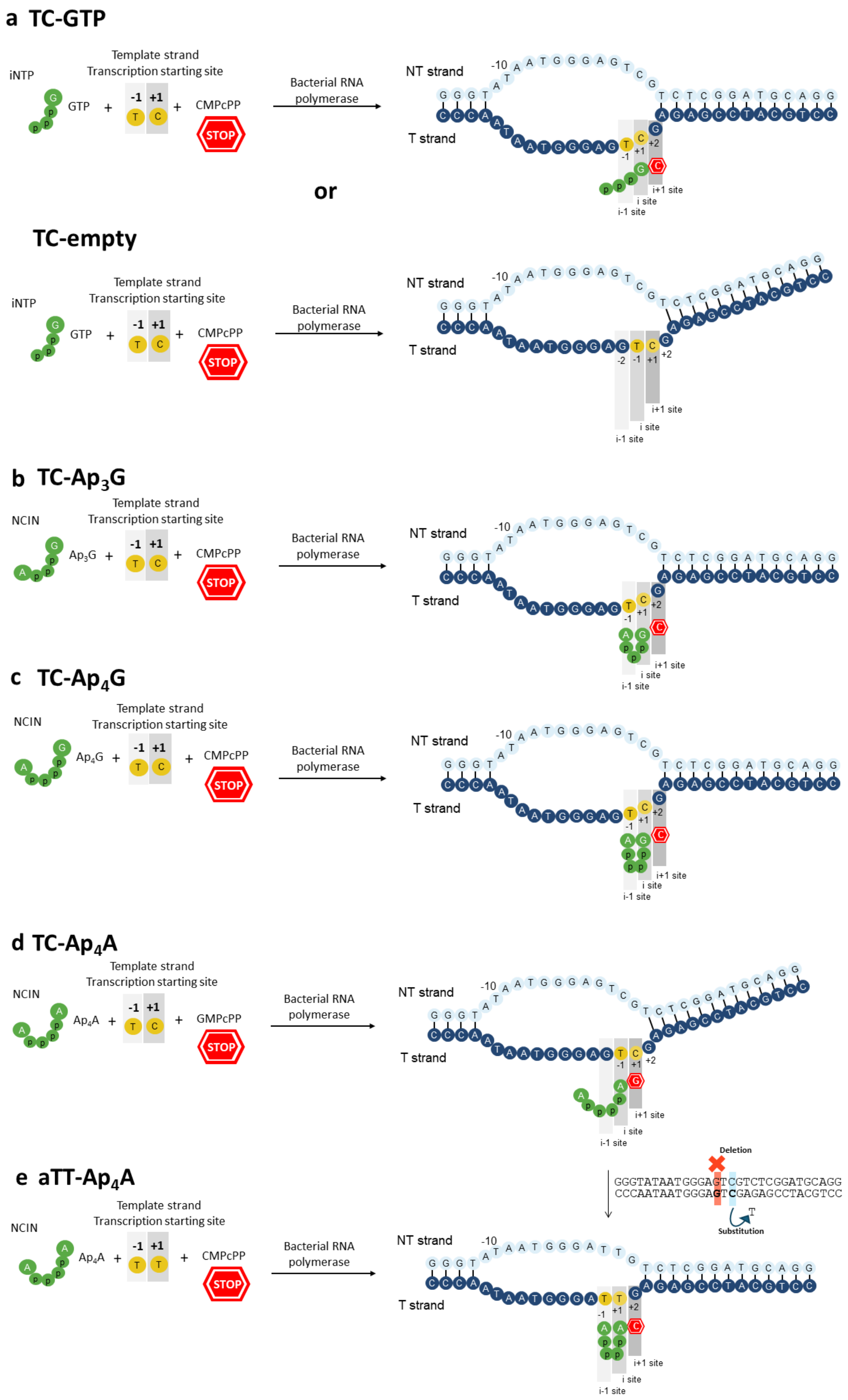

Supplementary Figure 4: Design of the stalled complexes for cryo-EM studies.

a, Design of stalled complex of template TC (−1T+1C), GTP (green) and the non-hydrolyzable CTP analog CMPcPP (red stop sign). In the cryo-EM analysis, we observed two types of structures, with bound nucleotides (TC-GTP) and empty active site (TC-empty). When the active site is unoccupied, the template strand is anti-scrunched by one nucleotide in respect to when the active site is occupied by GTP and CMPcPP.

b, Design of stalled complex of template TC, NCIN Ap_3G (green) and the non-hydrolyzable CTP analog CMPcPP (red stop sign). Template TC motif is aligned with the i −1 and i site, respectively, to allow base paring of both Ap_3G bases with the template strand.

c, Design of stalled complex of template TC, NCIN Ap_4G and the non-hydrolyzable CTP analog CMPcPP. The −1T+1C motif in the template is aligned with the i −1 and i site, respectively, to allow base paring of both Ap_4G bases with the template strand.

d, Design of stalled complex of template TC, NCIN Ap_4A and the non-hydrolyzable GTP analog GMPcPP. The template strand is anti-scrunched (highlighted by straightening of the template strand in the transcription bubble) by one nucleotide with respect to the RNAP i site. Template −1T is aligned with the i site to allow base paring of the A base from Ap_4A with the template strand.

e, Design of stalled complex of template aTT, NCIN Ap_4A and the non-hydrolyzable CTP analog CMPcPP. The aTT template has a substitution in position +1 (highlighted in light blue) and a deletion in position −2 (highlighted in red), so the transcription bubble is one nucleotide shorter than in the TC template (highlighted by straightening of both template strands in the transcription bubble). This enables to align the −1T+1T motif in the template with the i −1 and i site, respectively, to allow base paring of both Ap_4A bases with the template strand.

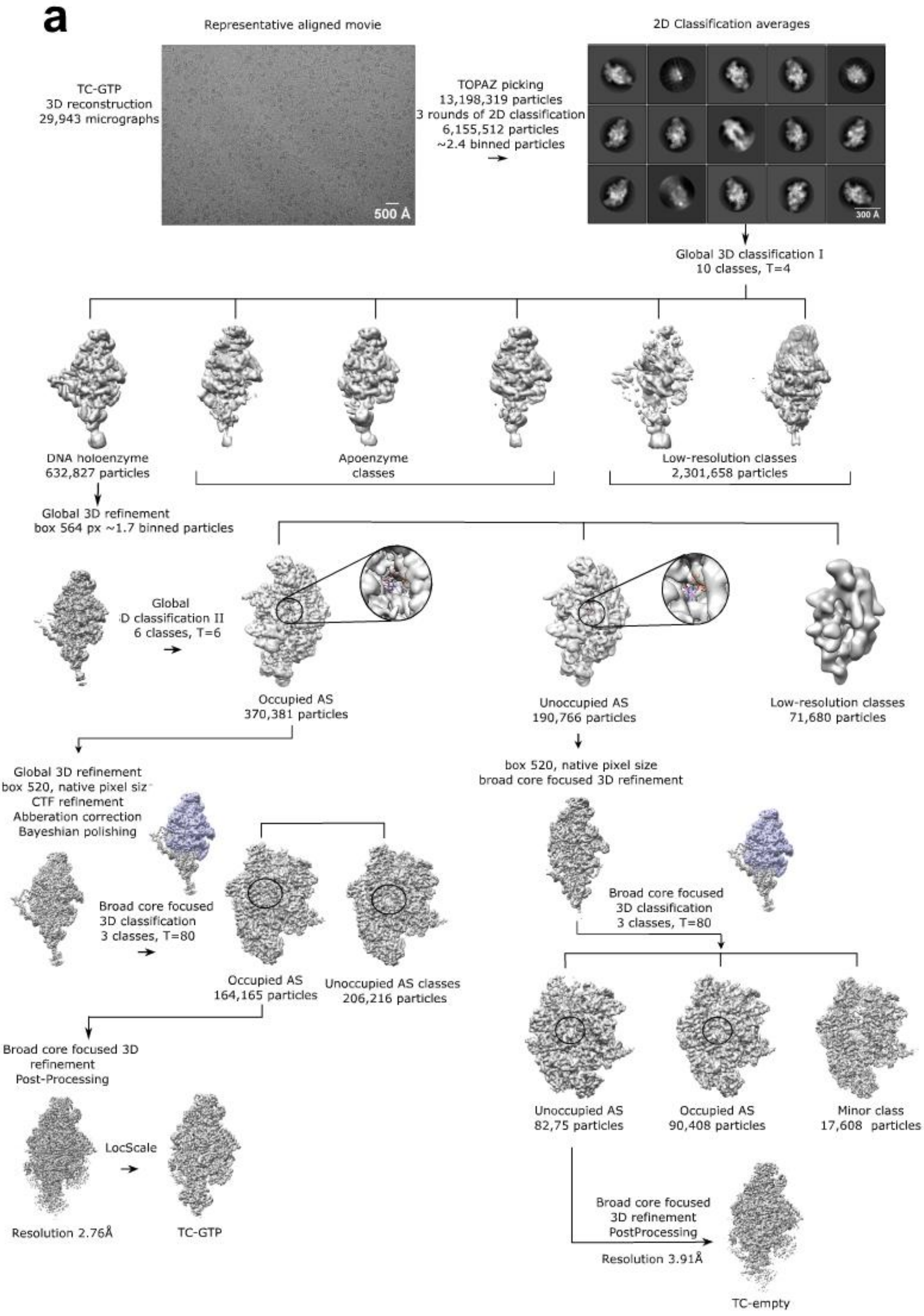
a
Representative aligned movie
2D Classification averages
TC-GTP
3D reconstruction
29,943 micrographs
500 Å
TOPAZ picking
13,198,319 particles
3 rounds of 2D classification
6,155,512 particles
~2.4 binned particles
300 Å
Global 3D classification I
10 classes, T=4
DNA holoenzyme
632,827 particles
Apoenzyme
classes
Low-resolution classes
2,301,658 particles
Global 3D refinement
box 564 px ~1.7 binned particles
Global
D classification II
6 classes, T=6
Occupied AS
370,381 particles
Unoccupied AS
190,766 particles
Low-resolution classes
71,680 particles
Global 3D refinement
box 520, native pixel siz
CTF refinement
Abberation correction
Bayeshian polishing
Broad core focused
3D classification
3 classes, T=80
Occupied AS
164,165 particles
Unoccupied AS classes
206,216 particles
Broad core focused 3D
refinement
Post-Processing
LocScale
Resolution 2.76Å
TC-GTP
box 520, native pixel size
broad core focused 3D refinement
Broad core focused
3D classification
3 classes, T=80
Unoccupied AS
82,75 particles
Occupied AS
90,408 particles
Minor class
17,608 particles
Broad core focused
3D refinement
PostProcessing
Resolution 3.91Å
TC-empty

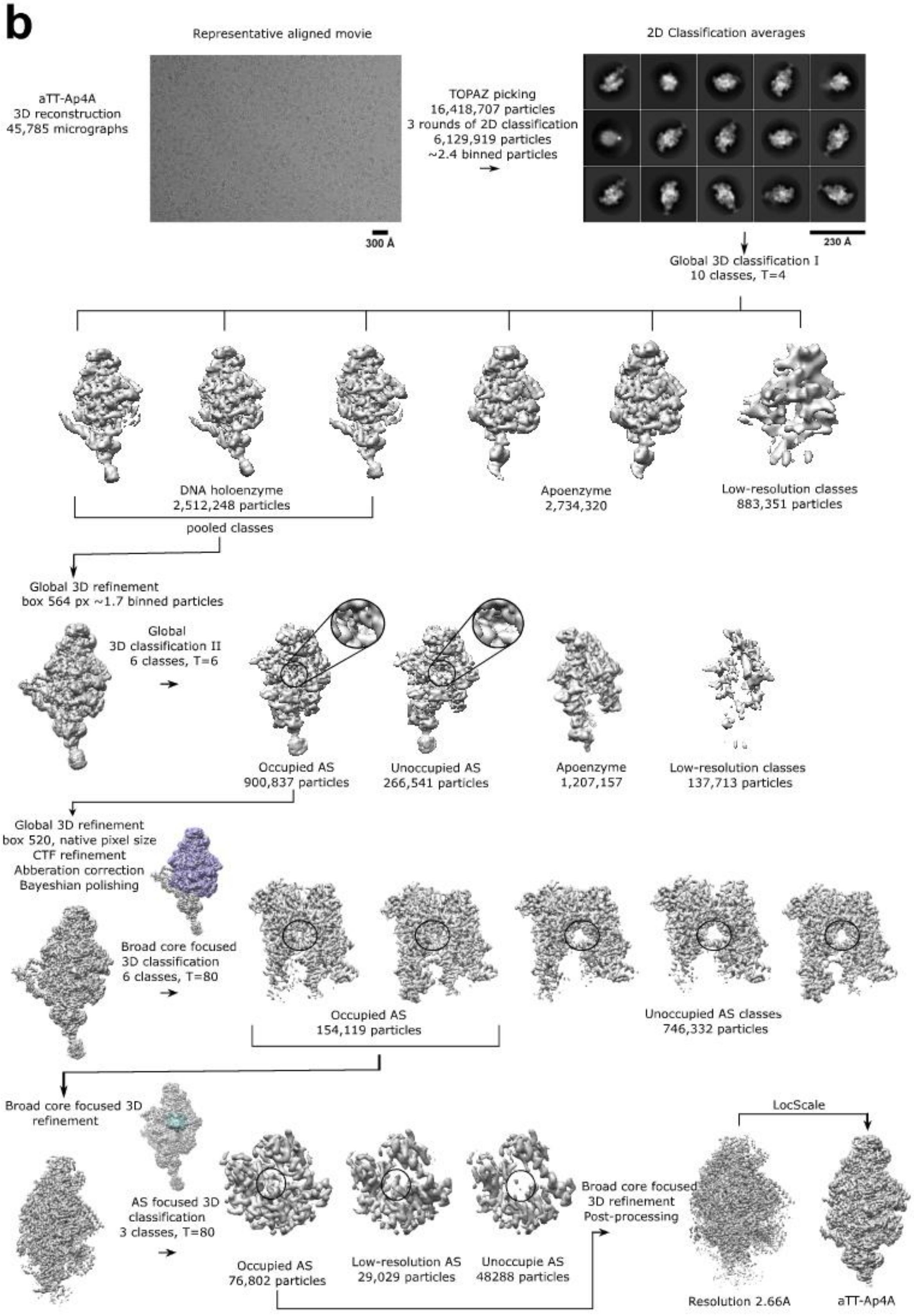
b
Representative aligned movie
2D Classification averages
aTT-Ap4A
3D reconstruction
45,785 micrographs
TOPAZ picking
16,418,707 particles
3 rounds of 2D classification
6,129,919 particles
~2.4 binned particles
300 Å
230 Å
Global 3D classification I
10 classes, T=4
DNA holoenzyme
2,512,248 particles
Apoenzyme
2,734,320
Low-resolution classes
883,351 particles
pooled classes
Global 3D refinement
box 564 px ~1.7 binned particles
Global
3D classification II
6 classes, T=6
Occupied AS
900,837 particles
Unoccupied AS
266,541 particles
Apoenzyme
1,207,157
Low-resolution classes
137,713 particles
Global 3D refinement
box 520, native pixel size
CTF refinement
Abberation correction
Bayeshian polishing
Broad core focused
3D classification
6 classes, T=80
Occupied AS
154,119 particles
Unoccupied AS classes
746,332 particles
Broad core focused 3D
refinement
AS focused 3D
classification
3 classes, T=80
Occupied AS
76,802 particles
Low-resolution AS
29,029 particles
Unoccupie AS
48288 particles
Broad core focused
3D refinement
Post-processing
LocScale
Resolution 2.66A
aTT-Ap4A

Supplementary Figure 5: Cryo-EM data processing workflow for the *Tt* RNAP *de novo* transcription initiation complexes.

Cryo-EM single particle analysis general data processing workflow is shown for the TC-GTP/empty (**a**) and aTT-Ap$_4$A (**b**) example datasets. The upper part shows representative micrographs (number of micrographs for all datasets is stated Supplementary Table 13) of the complexes in free standing ice after MotionCor2 correction at a defocus of ~2.5 µm, together with 2D-class averages. The lower part shows a summary of the cryo-EM 3D classification and refinement scheme together with representative focused classification masks. AS: active site.

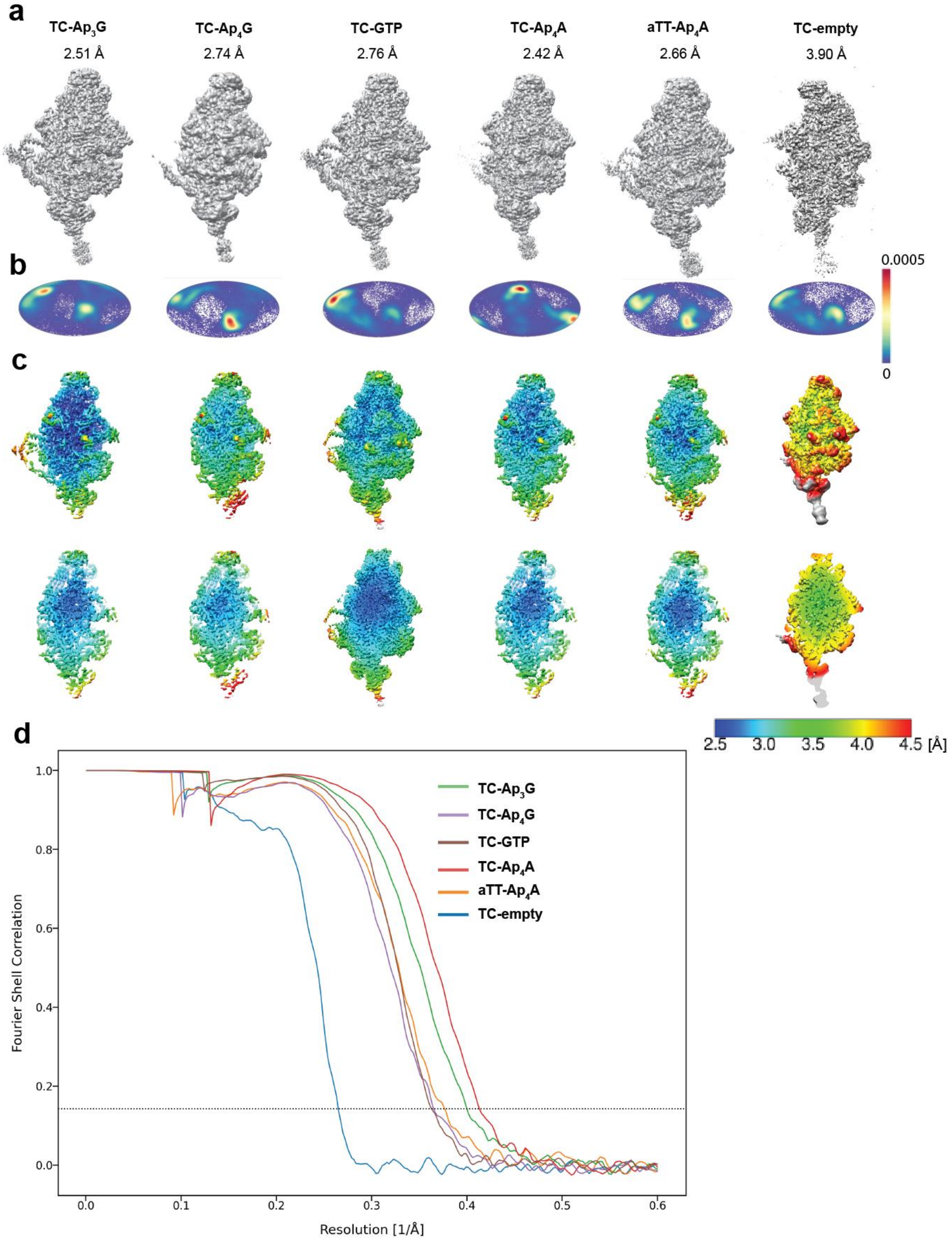

Supplementary Figure 6: Final 3D reconstructions of the *Tt* RNAP *de novo* transcription initiation complexes listed in Supplementary Table 7.

a, Final 3D reconstructions of the *Tt* RNAP *de novo* transcription initiation complexes listed in Supplementary Table 7 are shown together with (**b**) angular distributions of particle projections of the individual complexes on a globe-like plane. Every point represents a particle orientation and the color scale represents the normalized density of views around this point. The color scale ranges from 0 (low, blue) to 0.0005 (high, red).

c, Surface (**top**) and slice (**bottom**) representation of local resolution distribution. The maps are colored according to the local resolution calculated within the RELION software package. Local resolution range is indicated in the color bar.

d, The plot of the FSC between two independently refined half-maps shows the overall resolution of the two maps, as indicated by the gold standard FSC 0.143 cut-off criterion.

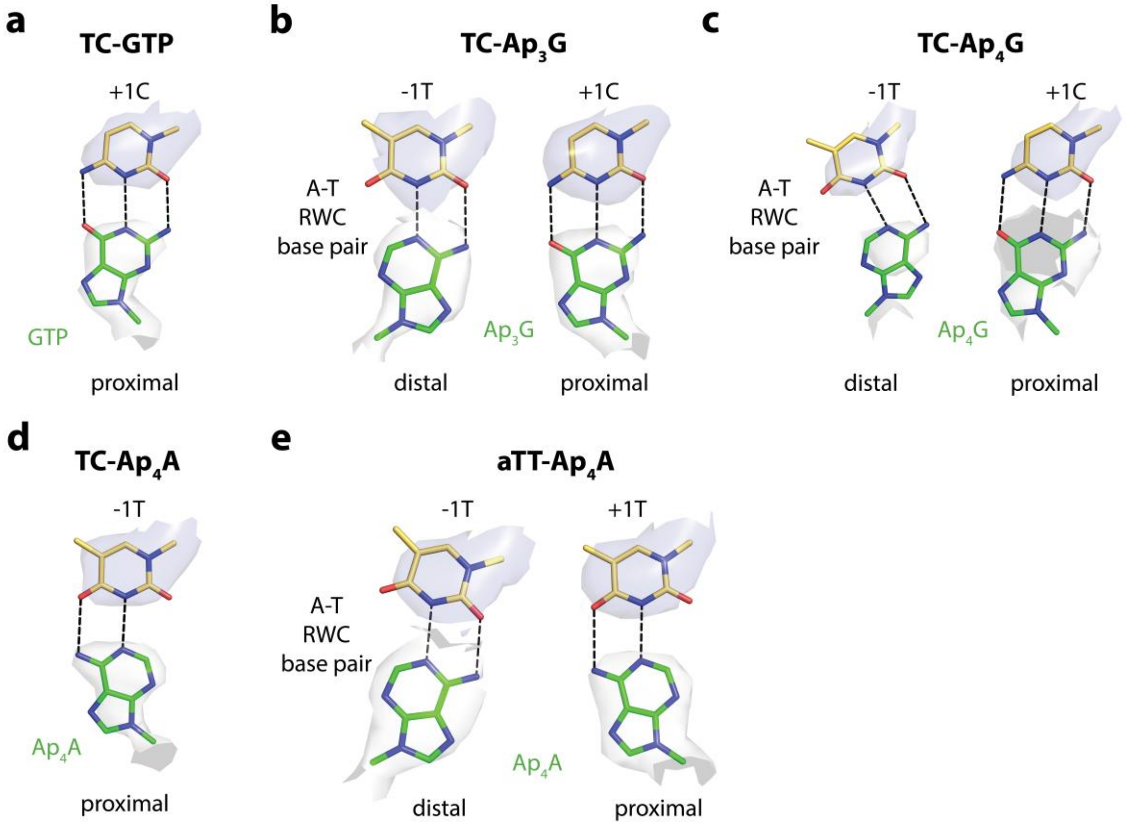

Supplementary Figure 7: Cryo-EM densities for the template-NCIN base pairs.

a, TC-GTP: cryo-EM density (mtz, blurring B = 50 Å^2) for the pairing bases of GTP (Pymol map contour level 4, transparent grey) and template (Pymol map contour level 4, transparent blue).

b, TC-Ap$_3$G: cryo-EM density (mtz, blurring B = 50 Å^2) for the pairing bases of Ap$_3$G (Pymol map contour level 0.8, transparent grey) and template (Pymol map contour level 3, transparent blue).

c, TC-Ap$_4$G: cryo-EM density (mtz, blurring B = 50 Å^2) for the pairing bases of Ap$_4$G (Pymol map contour level 4, transparent grey) and template (Pymol map contour level 8, transparent blue).

d, TC-Ap$_4$A: cryo-EM density (mtz, blurring B = 50 Å^2) for the pairing bases of Ap$_4$A (Pymol map contour level 0.6, transparent grey) and template (Pymol map contour level 5, transparent blue).

e, TT-Ap$_4$A: cryo-EM density (mtz, blurring B = 50 Å^2) for the pairing bases of Ap$_4$A (Pymol map contour level 5, transparent grey) and template (Pymol map contour level 10, transparent blue).

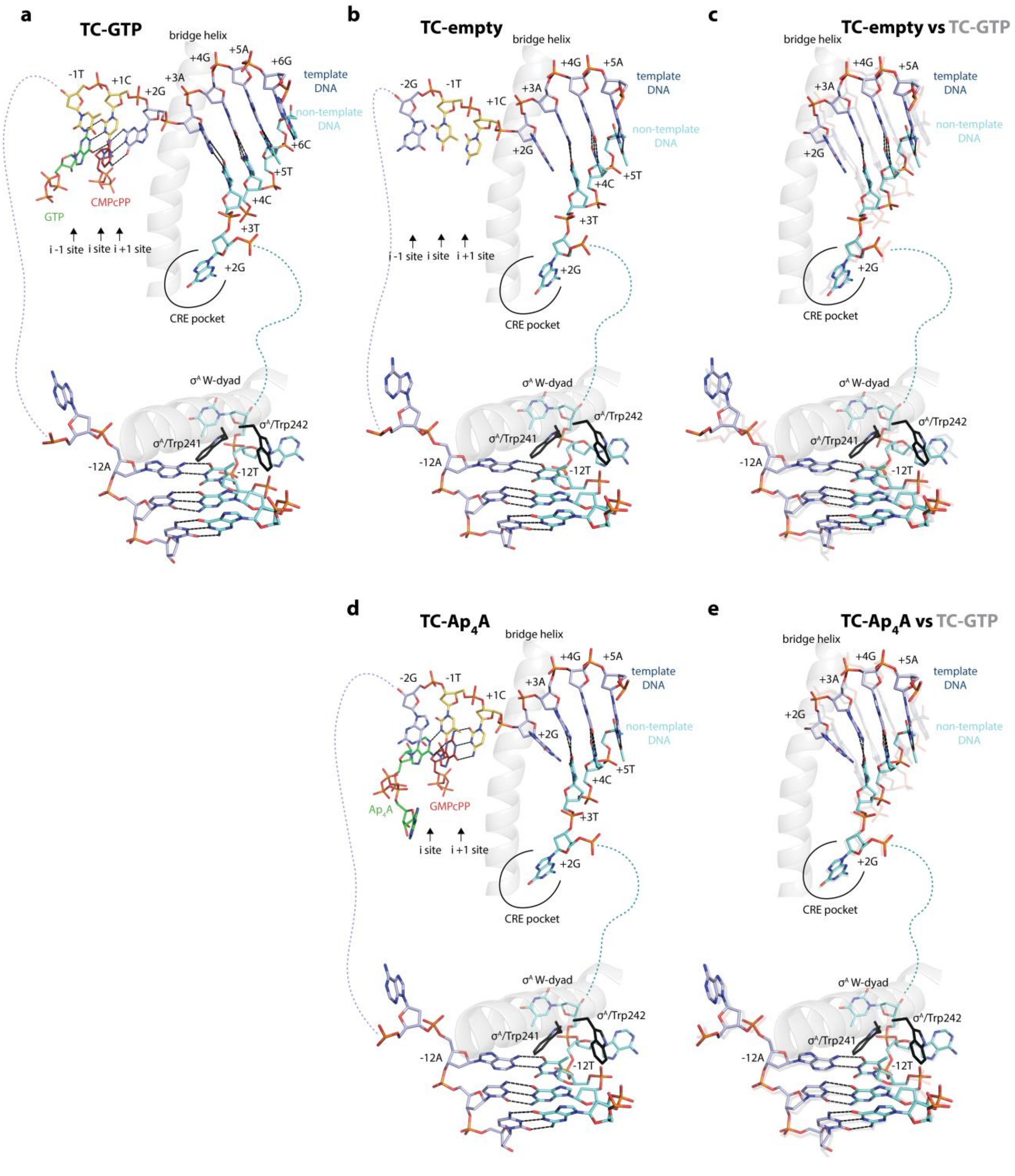

Supplementary Figure 8: Comparison of the regular and anti-scrunched template strand.

a, Regular template strand in TC-GTP. The color code of individual features corresponds to Figure 1b and Supplementary Figure 4a. **Top**: a view of the region around the +1 site and dwDNA. +2G from the non-template strand occupies the CRE pocket. **Bottom**: a view of the upstream DNA (upDNA) duplex. Upstream DNA duplex starting at position -12 is canonically positioned by the σ^A W-dyad.

b, Anti-scrunched template strand in TC-empty. The color code of individual features corresponds to Figure 1b. **Top**: a view of the region around the +1 site and dwDNA. Template strand +2G is shifted over the bridge helix towards the dwDNA duplex. The non-template strand is not shifted in register and +2G occupies the CRE pocket. **Bottom**: a view of the upDNA, canonically positioned by the σ^A W-dyad.

c, Superposition of the +1 region and dwDNA (**top**, aligned by β residues 397-700) and the upDNA (**bottom**, aligned by σ^A residues 195-250) of TC-empty (in color) and TC-GTP (transparent grey). The bridge helix and the σ^A W-dyad from TC-GTP is shown. The dwDNA duplex in TC-empty is shifted and slightly tilted to accommodate the bulged template +2G, while the upDNA is almost identical.

d, Anti-scrunched template strand in TC-Ap_4A. The color code of individual features corresponds to Figure 1b and 3a. **Top**: a view of the region around the +1 site and dwDNA. Template strand +2G is shifted over the bridge helix towards the dwDNA duplex. The non-template strand is not shifted in register and +2G occupies the CRE pocket. **Bottom**: a view of the upDNA, canonically positioned by the σ^A W-dyad.

e, Superposition of the +1 region and dwDNA (**top**, aligned by β residues 397-700) and the upDNA (**bottom**, aligned by σ^A residues 195-250) of TC-Ap_4A (in color) and TC-GTP (transparent grey). The bridge helix and the σ^A W-dyad from TC-GTP is shown. The dwDNA duplex in TC-Ap_4A is shifted and slightly tilted to accommodate the bulged template +2G, while the upDNA is almost identical.

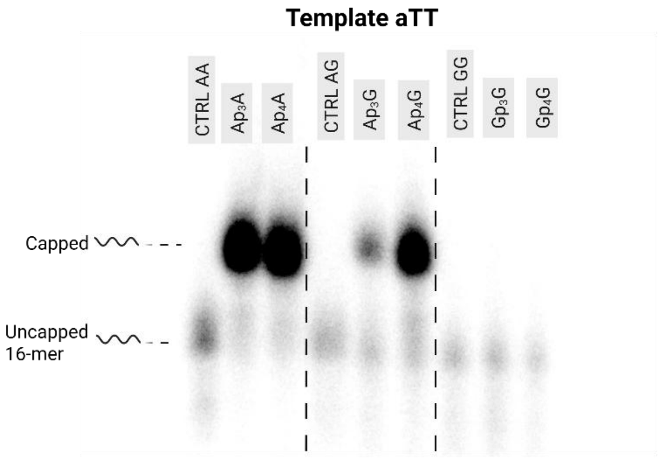

Supplementary Figure 9: PAGE analysis (with APB) of RNA products from IVT experiments with the aTT template and Np_nNs.

This PAGE analysis was performed (in single replicate) as a control before the cryo-EM experiment to verify detectable production of capped RNA. Each control, designed according to Supplementary Table 2, shows the migration of uncapped RNA and serves as a molecular weight marker for 16 nt. The addition of $Ap_{3\text{-}4}N$ resulted in the production of capped RNA, whereas the addition of $Gp_{3\text{-}4}G$ did not lead to any capped RNA production. Created in BioRender. Serianni, V. (2025) https://BioRender.com/gb3iluj.

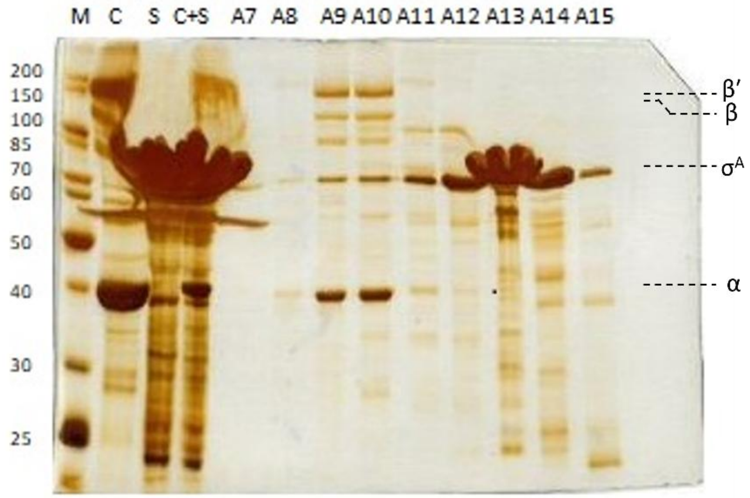

Supplementary Figure 10: SDS-PAGE analysis of *Tt* RNAP after size exclusion chromatography. M=protein ladder (molecular weight in kDa), C=RNAP core, S=σ^A, C+S= mixture of σ^A and RNAP core and A7-A15=size exclusion chromatography fractions of the holoenzyme. C, S, C+S lanes are not interpretable due to sigma overload. ω subunit (11.5 kDa) ran out of the gel. Silver staining is not proportional to the amount of protein in various bands and was used to emphasize impurities. The purification and SDS-PAGE analysis was performed three times.

Supplementary References

1 Zhang, Y. *et al.* Structural Basis of Transcription Initiation. *Science* **338**, 1076-1080 (2012).

2 Shi, J. *et al.* Structural basis of Mfd-dependent transcription termination. *Nucleic Acids Research* **48**, 11762-11772 (2020).

3 Bird, J. G. *et al.* The mechanism of RNA 5′ capping with NAD+, NADH and desphospho-CoA. *Nature* **535**, 444 (2016).

Source Data Files for SI

Uncropped gel of Supplementary Figure 3

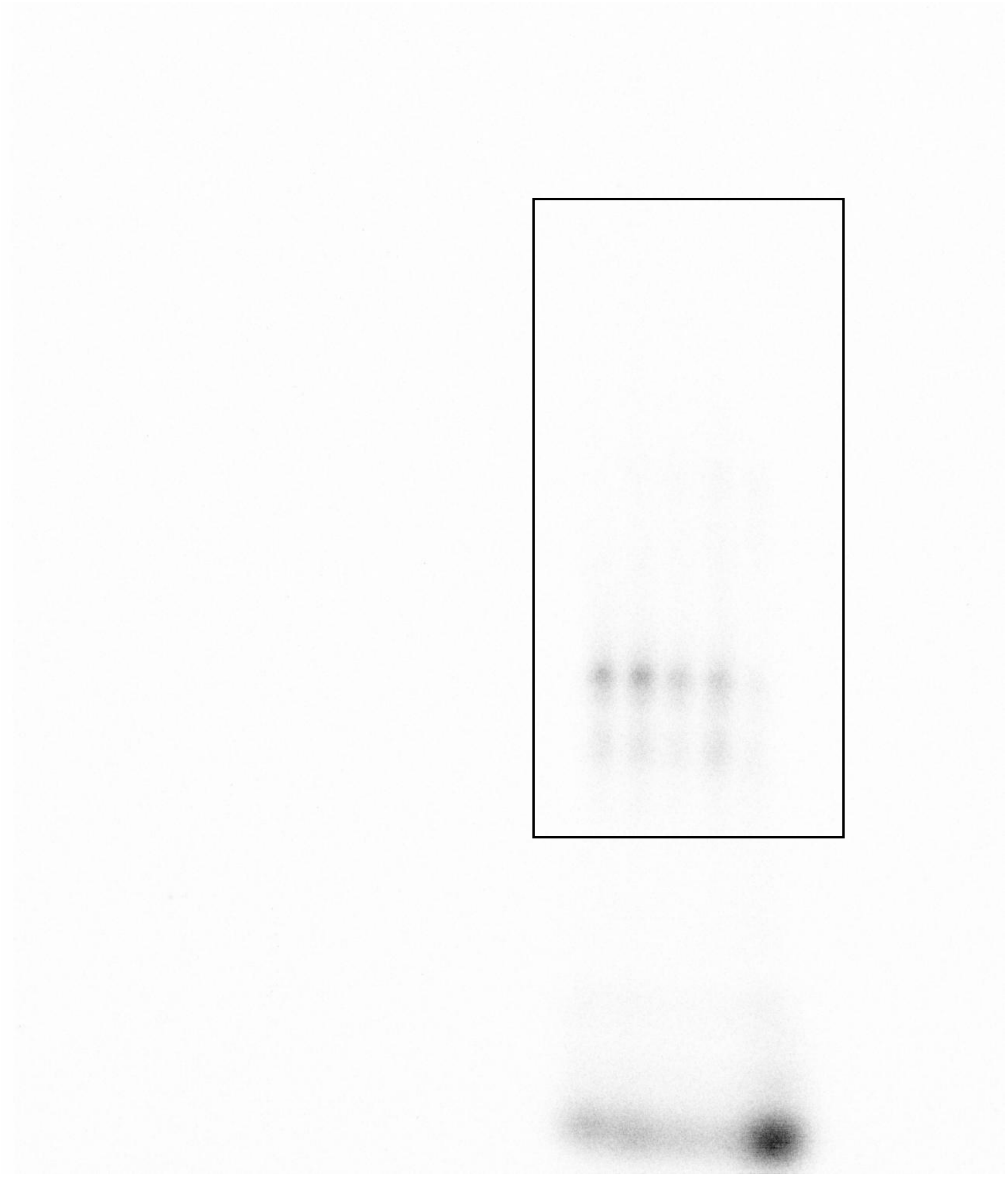

Uncropped gel of Supplementary Figure 3

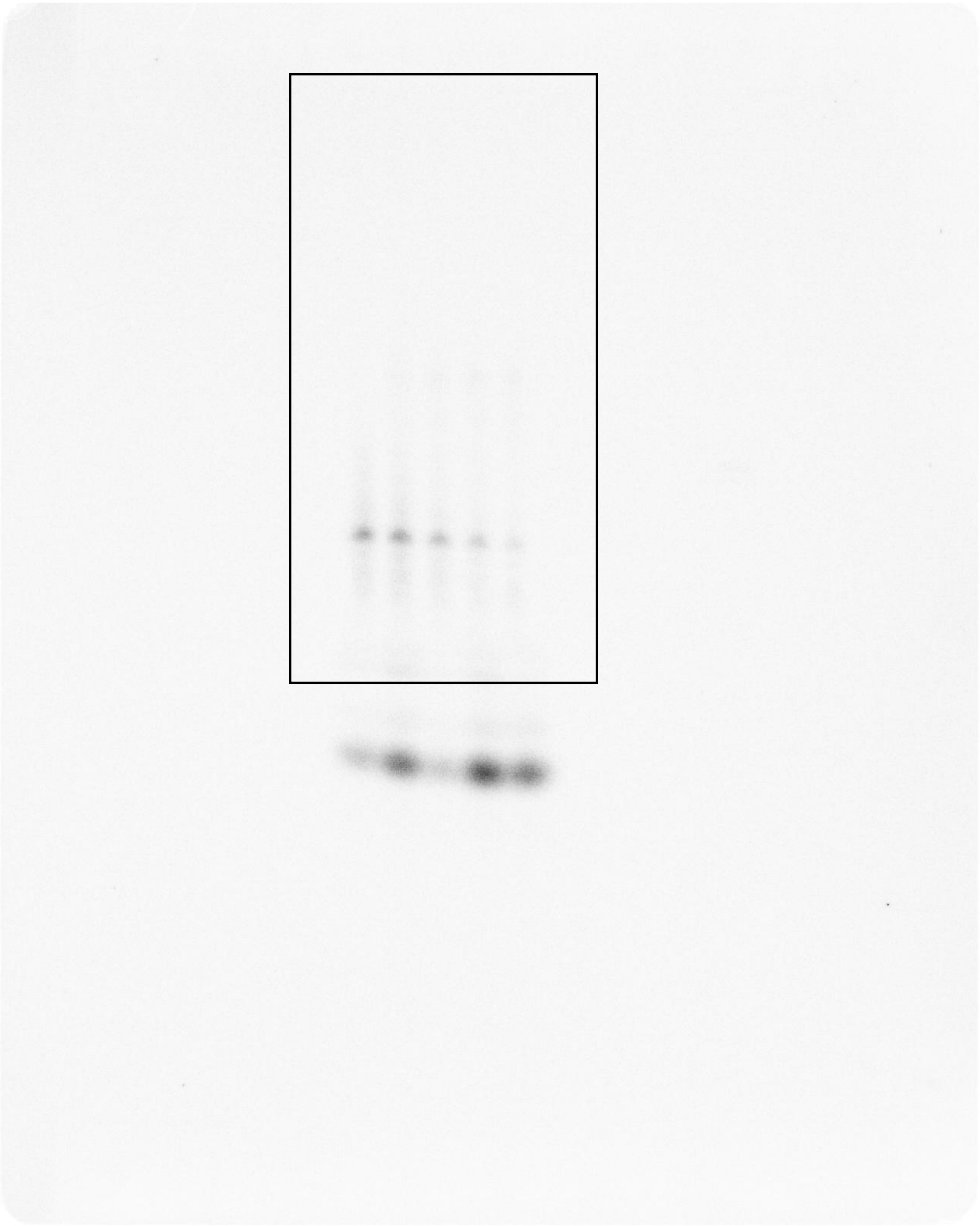

Uncropped gel of Supplementary Figure 3

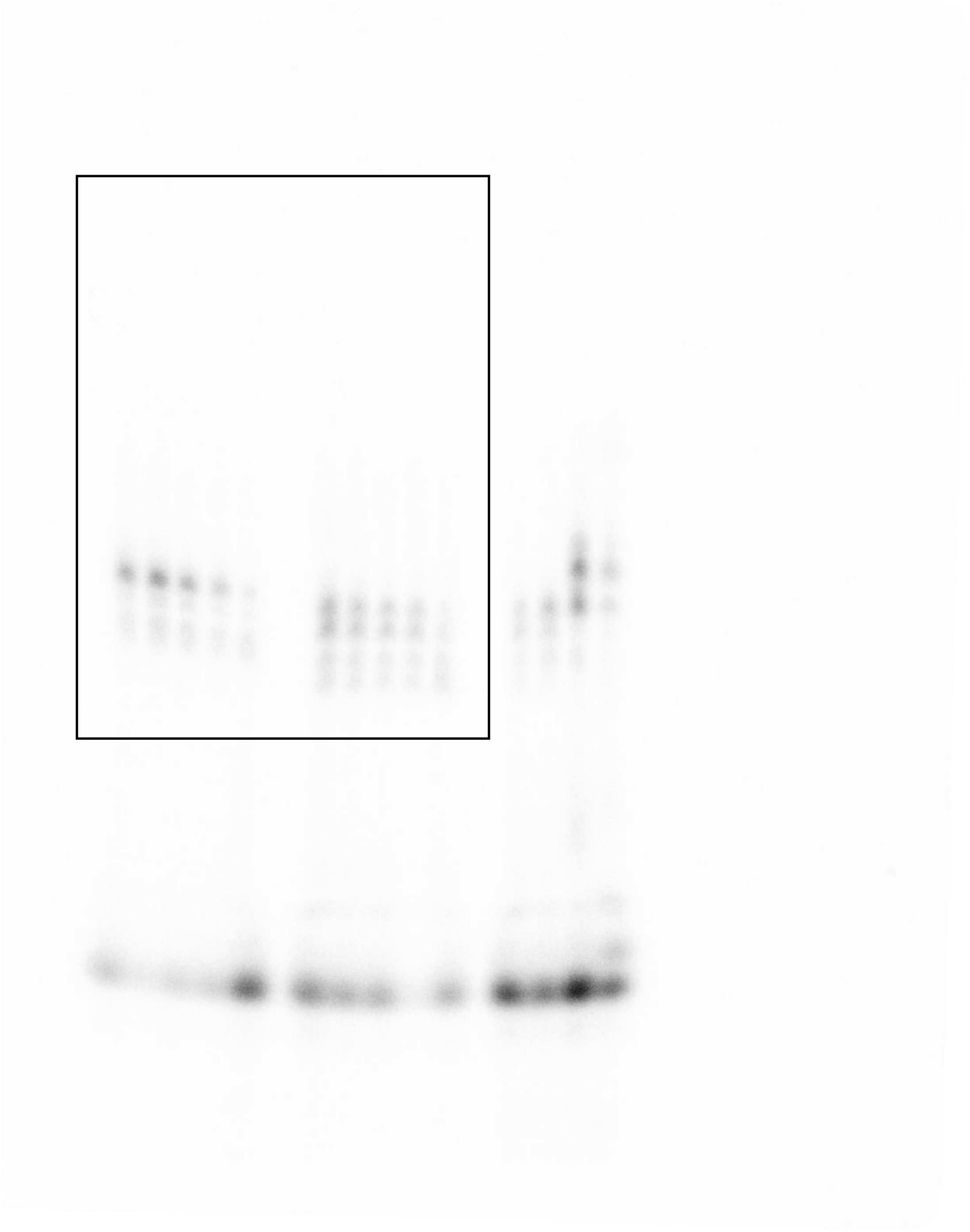

Uncropped gel of Supplementary Figure 9

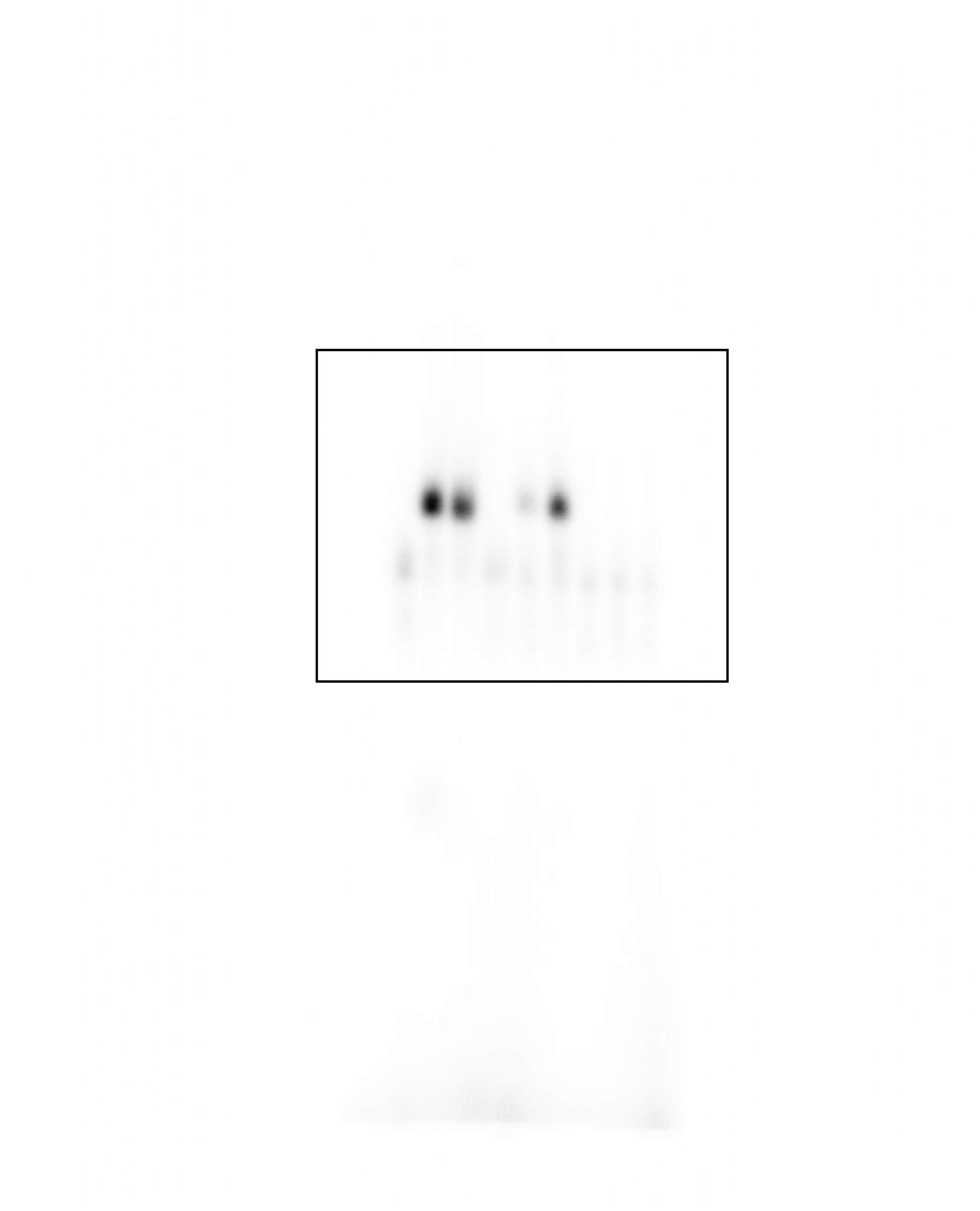

Supplement: Supplementary file 1 — Supplementary Tables 1–12, Supplementary Figs. 1–10, Supplementary References and Source Data for Supplementary Information. [file 41589_2025_2134_MOESM1_ESM.pdf]
