# Supplementary material for: Molecular insight into 5′ RNA capping with NpnNs by bacterial RNA polymerase
Source: Nat Chem Biol. 2026 Jan 9;22(6):917–24. doi: 10.1038/s41589-025-02134-5 (PMC13226041; doi:10.1038/s41589-025-02134-5)
Supplement: Supplementary file 2 — Reporting Summary [file 41589_2025_2134_MOESM2_ESM.pdf]

nature portfolio

Corresponding author(s): Tomáš Kouba, Hana Cahova

Last updated by author(s): Dec 8, 2025

Reporting Summary

Nature Portfolio wishes to improve the reproducibility of the work that we publish. This form provides structure for consistency and transparency in reporting. For further information on Nature Portfolio policies, see our Editorial Policies and the Editorial Policy Checklist.

Statistics

For all statistical analyses, confirm that the following items are present in the figure legend, table legend, main text, or Methods section.

n/a	Confirmed	
☐	☒	The exact sample size (*n*) for each experimental group/condition, given as a discrete number and unit of measurement
☐	☒	A statement on whether measurements were taken from distinct samples or whether the same sample was measured repeatedly
☒	☐	The statistical test(s) used AND whether they are one- or two-sided<br>*Only common tests should be described solely by name; describe more complex techniques in the Methods section.*
☒	☐	A description of all covariates tested
☒	☐	A description of any assumptions or corrections, such as tests of normality and adjustment for multiple comparisons
☐	☒	A full description of the statistical parameters including central tendency (e.g. means) or other basic estimates (e.g. regression coefficient) AND variation (e.g. standard deviation) or associated estimates of uncertainty (e.g. confidence intervals)
☒	☐	For null hypothesis testing, the test statistic (e.g. *F*, *t*, *r*) with confidence intervals, effect sizes, degrees of freedom and *P* value noted<br>*Give P values as exact values whenever suitable.*
☒	☐	For Bayesian analysis, information on the choice of priors and Markov chain Monte Carlo settings
☒	☐	For hierarchical and complex designs, identification of the appropriate level for tests and full reporting of outcomes
☒	☐	Estimates of effect sizes (e.g. Cohen's *d*, Pearson's *r*), indicating how they were calculated

Our web collection on statistics for biologists contains articles on many of the points above.

Software and code

Policy information about availability of computer code

Data collection	Titan Krios (FEI) + Serial EM 4.1, LC-MS: Acquity H-class + Xevo G2-XS QTof mass spectrometer (Waters), Typhoon FLA 9500
Data analysis	MotionCor2 v1.3.1, CTFFIND4.1, TOPAZ v0.2.5, RELION 4.0, CCP-EM 1.5, LocScale 0.1, cryoEF v1.1.0, Molprep 11.7.02, Coot 0.9.8.92, CCP4 Interface 8.0.010, libG under Refmac 5.8.0405, Phenix 1.21-5207, Grade Web Server 2.0.14, MolProbity Server 4.5.2, GraphPad Prism 10, MassLynx V4.2, ImageJ 1.53e

For manuscripts utilizing custom algorithms or software that are central to the research but not yet described in published literature, software must be made available to editors and reviewers. We strongly encourage code deposition in a community repository (e.g. GitHub). See the Nature Portfolio guidelines for submitting code & software for further information.

Data

Policy information about availability of data

All manuscripts must include a data availability statement. This statement should provide the following information, where applicable:

- Accession codes, unique identifiers, or web links for publicly available datasets
- A description of any restrictions on data availability
- For clinical datasets or third party data, please ensure that the statement adheres to our policy

All data generated in this study are available within the article and supplementary information. Source data are provided with this paper paper in Source data File. LC-MS data are available at 10.5281/zenodo.14215050. Co-ordinates and maps for Thermus thermophilus RNA polymerase de novo transcription initiation

complexes have been deposited in the wwPDB and EMDB databases under the following accession numbers: TC-Ap3G - 9FOG and EMD-50622, TC-Ap4G - 9FOK and EMD-50625, TC-Ap4A - 9FP3 and EMD-50634, TC-GTP - 9FO6 and EMD-50618, aTT-Ap4A - 9FRJ and EMD-50715, TC-empty - 9R75 and EMD-53711.

Research involving human participants, their data, or biological material

Policy information about studies with human participants or human data. See also policy information about sex, gender (identity/presentation), and sexual orientation and race, ethnicity and racism.

Reporting on sex and gender	Not relevant, since no experiments on humans were performed in this study.
Reporting on race, ethnicity, or other socially relevant groupings	Not relevant, since no experiments on humans were performed in this study.
Population characteristics	Not relevant, since no experiments on humans were performed in this study.
Recruitment	Not relevant, since no experiments on humans were performed in this study.
Ethics oversight	Not relevant, since no experiments on humans were performed in this study.

Note that full information on the approval of the study protocol must also be provided in the manuscript.

Field-specific reporting

Please select the one below that is the best fit for your research. If you are not sure, read the appropriate sections before making your selection.

☒ Life sciences ☐ Behavioural & social sciences ☐ Ecological, evolutionary & environmental sciences

For a reference copy of the document with all sections, see nature.com/documents/nr-reporting-summary-flat.pdf

Life sciences study design

All studies must disclose on these points even when the disclosure is negative.

Sample size	For biochemical analyses, sample sizes of at least of 3 were chosen (experimental replicates).
Data exclusions	No data were excluded.
Replication	The number of times each experiment was replicated is indicated in the figure captions.
Randomization	not applicable
Blinding	not applicable

Reporting for specific materials, systems and methods

We require information from authors about some types of materials, experimental systems and methods used in many studies. Here, indicate whether each material, system or method listed is relevant to your study. If you are not sure if a list item applies to your research, read the appropriate section before selecting a response.

Materials & experimental systems

n/a	Involved in the study	
☒	☐	Antibodies
☒	☐	Eukaryotic cell lines
☒	☐	Palaeontology and archaeology
☒	☐	Animals and other organisms
☒	☐	Clinical data
☒	☐	Dual use research of concern
☒	☐	Plants

Methods

n/a	Involved in the study	
☒	☐	ChIP-seq
☒	☐	Flow cytometry
☒	☐	MRI-based neuroimaging

Plants

Seed stocks	Not relevant, as no experiments on plants were performed in this study
Novel plant genotypes	Not relevant, as no experiments on plants were performed in this study
Authentication	Not relevant, as no experiments on plants were performed in this study
