# Supplementary material for: Molecular insight into 5′ RNA capping with NpnNs by bacterial RNA polymerase
Source: Nat Chem Biol. 2026 Jan 9;22(6):917–24. doi: 10.1038/s41589-025-02134-5 (PMC13226041; doi:10.1038/s41589-025-02134-5)

Source Data Files for main text and Extended Data Figures

Uncropped gel of Figure 1d and Extended Data Figure 2

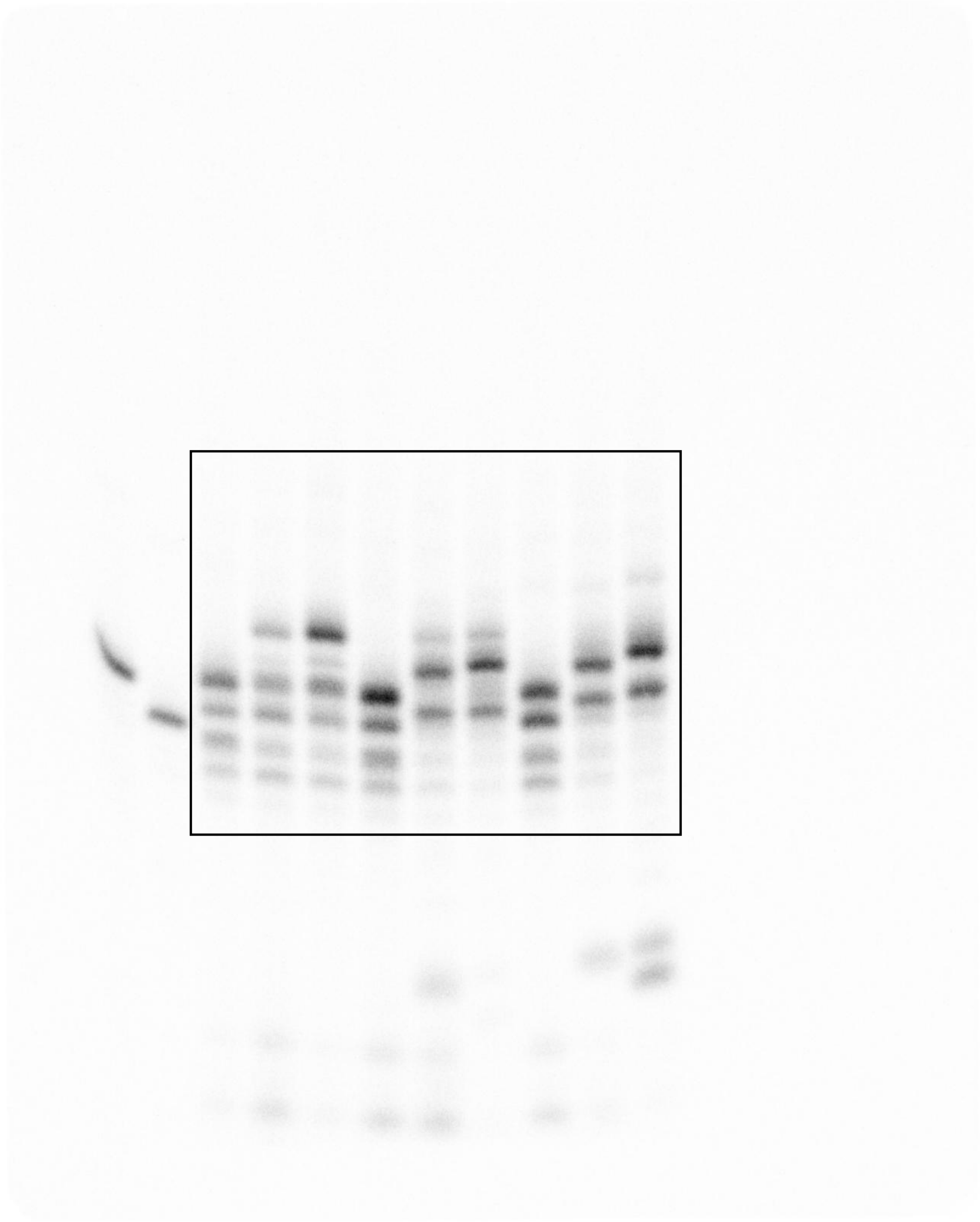

Uncropped gel of Figure 1e

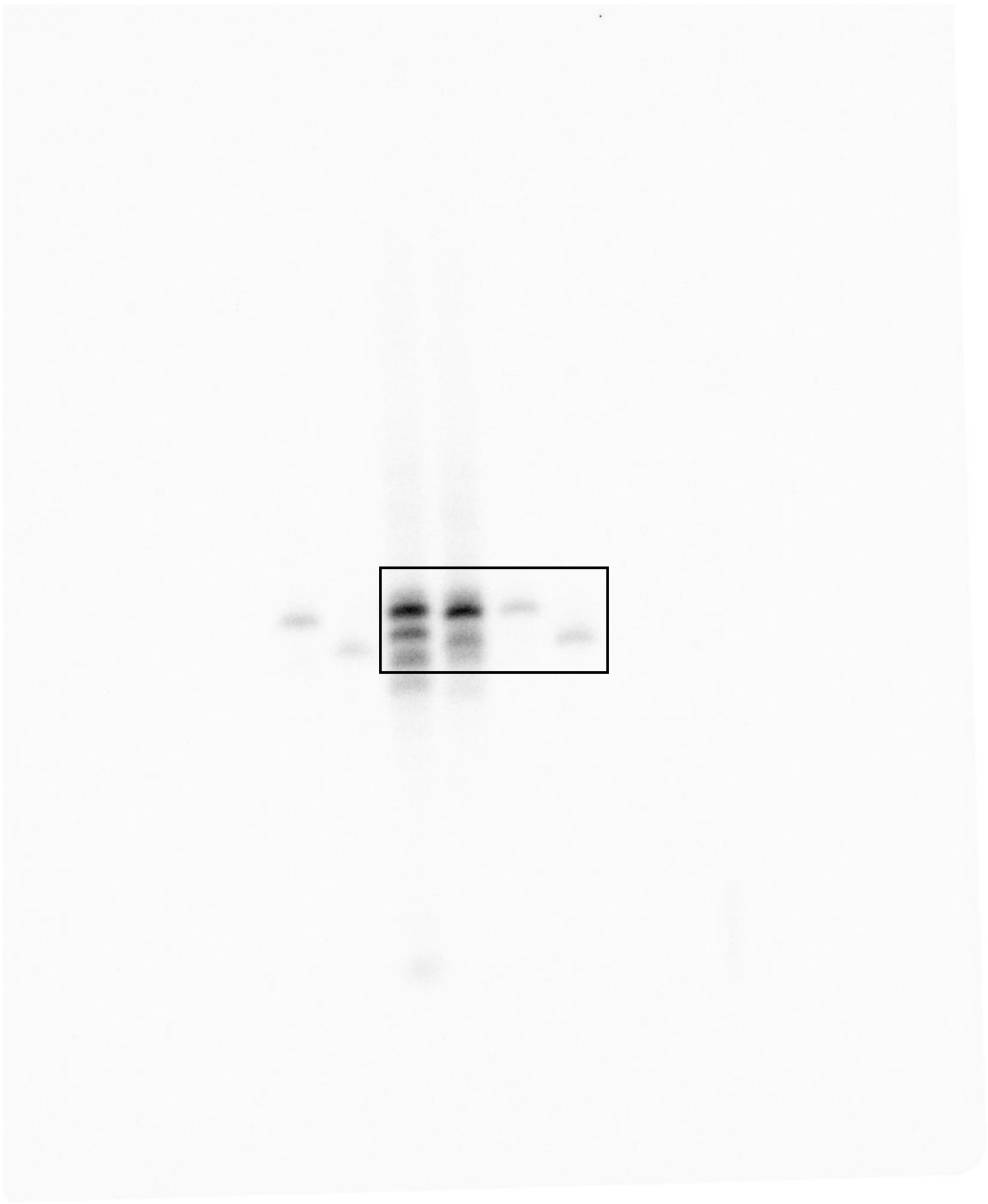

Uncropped gel of Extended Data Figure 3

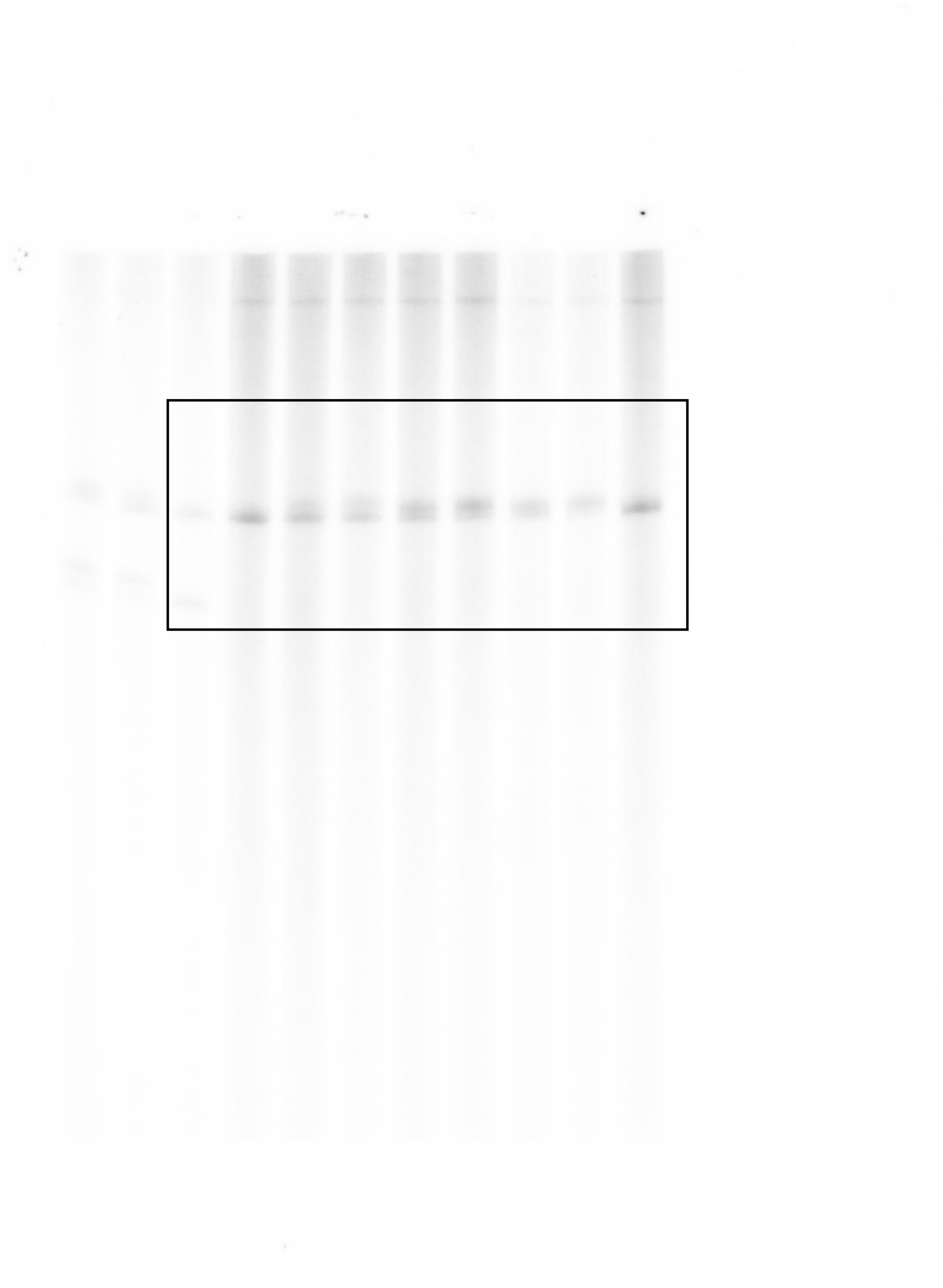

Uncropped gel of Extended Data Figure 1c and Extended Data Figure 5

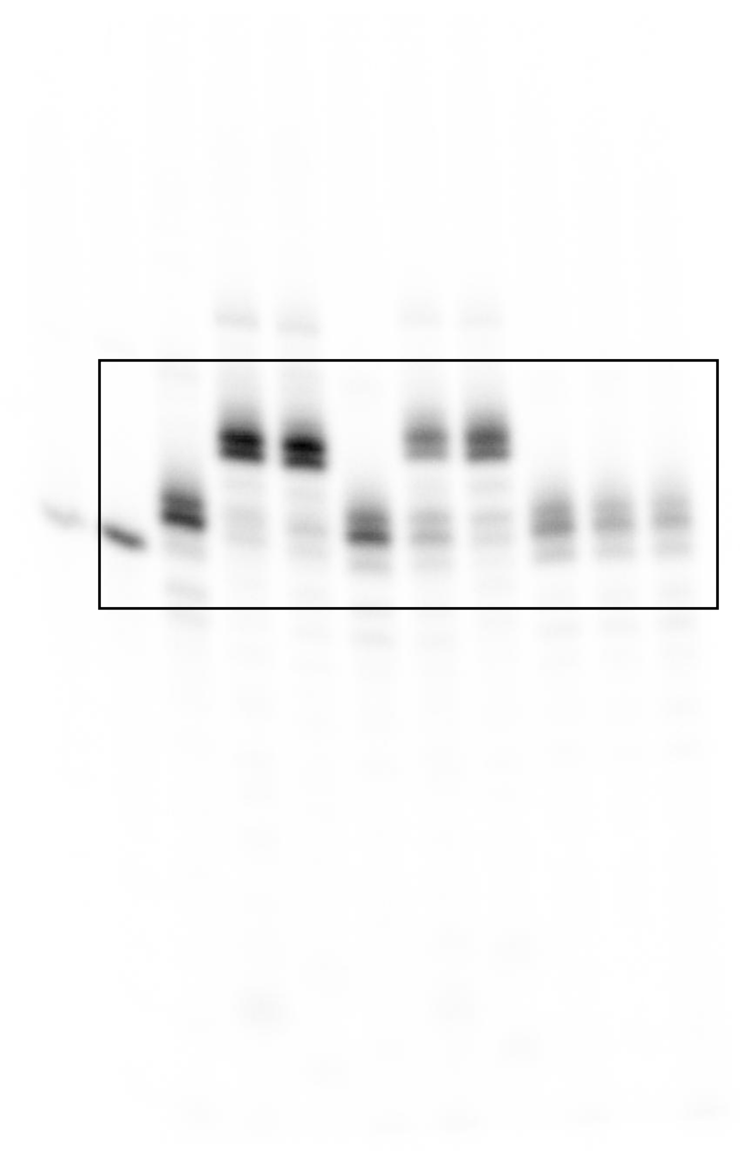

Uncropped gel of Extended Data Figure 2d

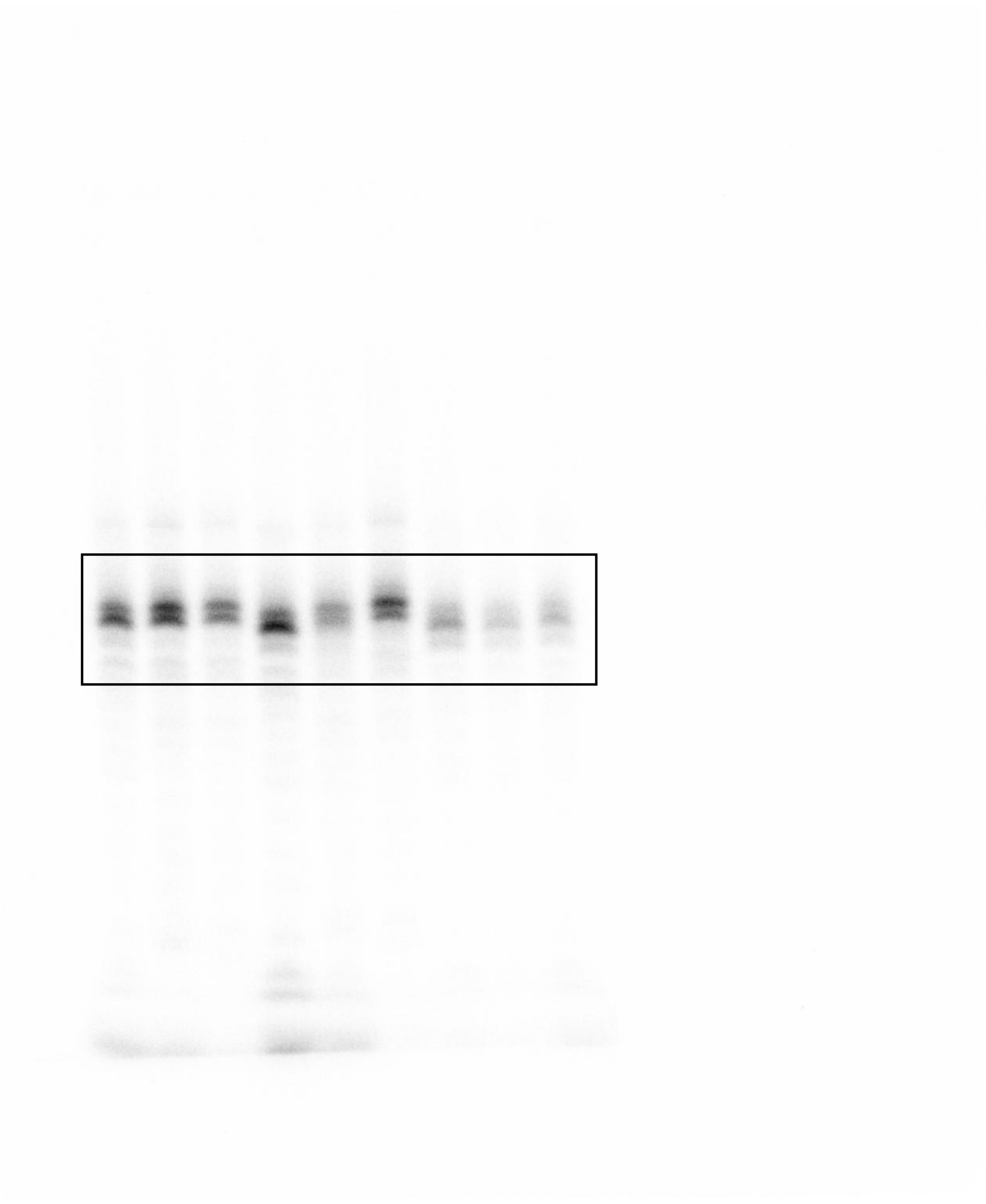

Uncropped gel Extended Data Figure 3e

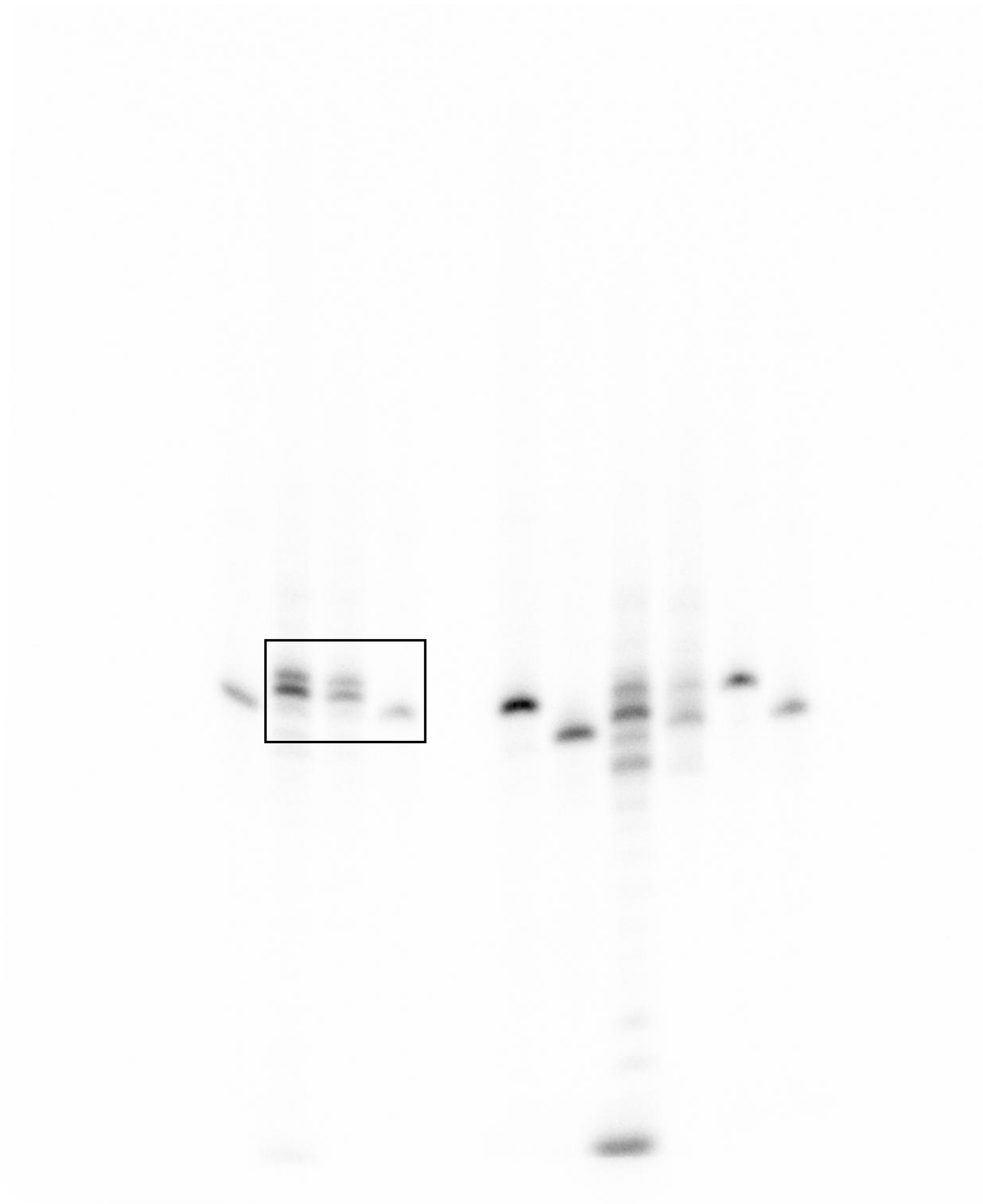

Uncropped gel of Extended Data Figure 6c and Extended Data Figure 7

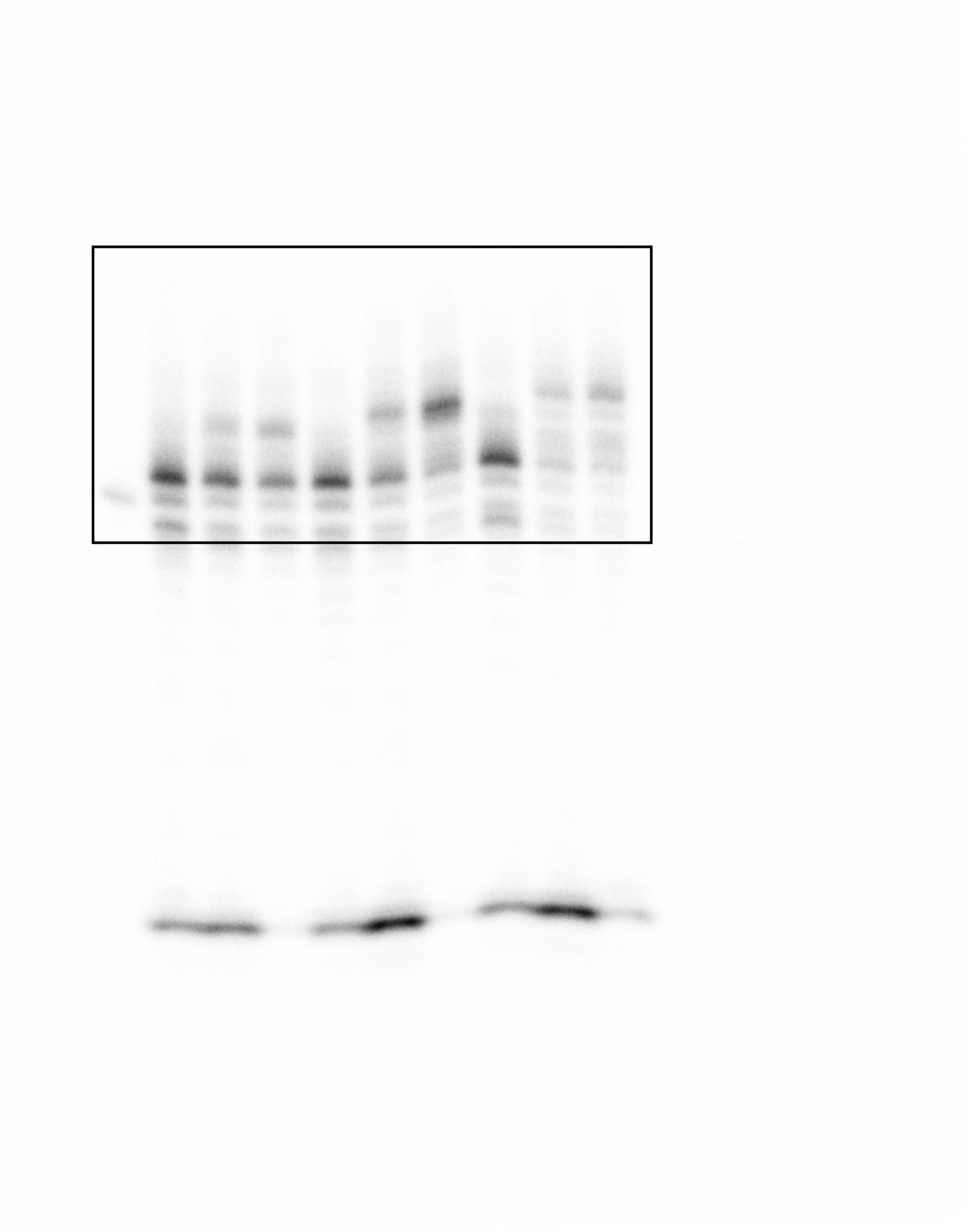

Uncropped gel of Extended Data Figure 6d

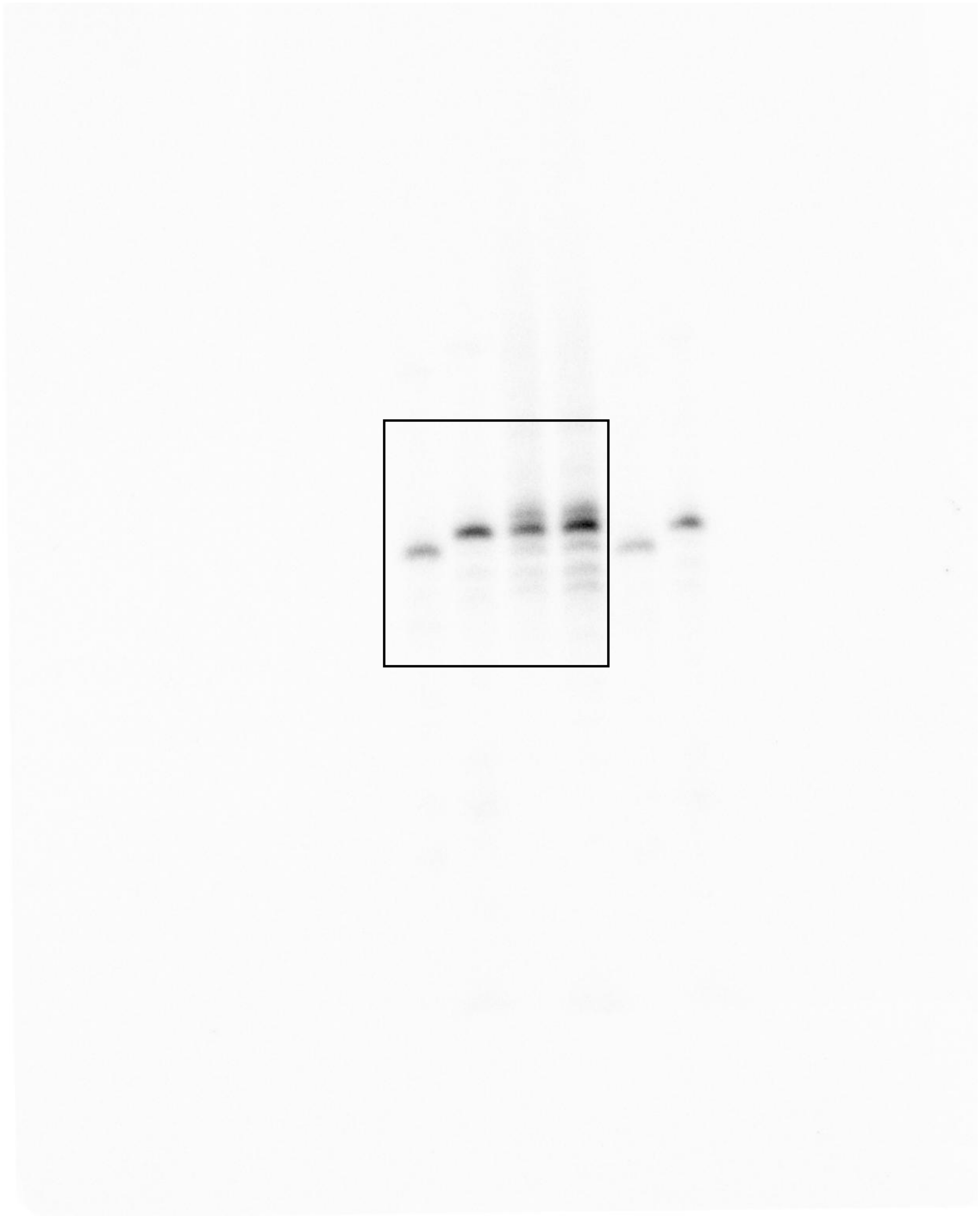

Supplement: Supplementary file 3 — Unprocessed gels. [file 41589_2025_2134_MOESM3_ESM.pdf]
